# Development of an effective illness severity measure and assessment of the impact of perceived illness severity on formal careseeking for fatal illnesses of neonates and infants in six sub-Saharan Africa countries and Pakistan

**DOI:** 10.1371/journal.pgph.0006455

**Published:** 2026-05-22

**Authors:** Henry D. Kalter, Jamie Perin, Zulfiqar A. Bhutta, Inuwa B. Jalingo, Paul R. Libite, Abdou Maina, Tiope Mleme, Mlemba A. Kamwe, Ivalda Macicame, Robert E. Black

**Affiliations:** 1 Institute for International Programs, Department of International Health, Johns Hopkins Bloomberg School of Public Health, Baltimore, Maryland, United States of America; 2 Center for Child and Community Health Research, Department of Pediatrics, Johns Hopkins School of Medicine, Baltimore, Maryland, United States of America; 3 Institute for Global Health & Development, Aga Khan University, Karachi, Pakistan; 4 SickKids Centre for Global Child Health, Hospital for Sick Children, Toronto, Ontario, Canada; 5 Census Department, National Population Commission, Abuja, Nigeria; 6 Institut National de la Statistique, Yaoundé, Cameroun; 7 Institut National de la Statistique, Niamey, Niger; 8 National Statistical Office, Zomba, Malawi; 9 National Bureau of Statistics, Dodoma, United Republic of Tanzania; 10 Instituto Nacional de Saúde, Maputo, Moçambique; Swiss Tropical and Public Health Institute: Schweizerisches Tropen- und Public Health-Institut, SWITZERLAND

## Abstract

Early careseeking for sick children can make the difference between life and death. Verbal autopsy (VA) studies of the cause of death typically ask about severe symptoms such as seizures and possibly mild or moderate symptoms such as rash, but without examining the relationship between caregivers’ perception of illness severity and appropriate careseeking. Verbal and social autopsy (VASA) is a newer method that builds on VA by also examining social factors related to death. From seven VASA studies conducted in Africa and Asia we developed a 2-sign method based on activity level and feeding behavior and a multiple sign method of identifying mild, moderate and severe illness of neonates and 1–11-month-olds. We then examined the relationship of caregivers’ perception of their child’s condition at illness onset and several covariates to seeking formal health care during the fatal illness. The 2-sign and multiple sign methods effectively distinguished mild, moderate, and severe illnesses, respectively, of neonates and 1–11-month-olds. Careseeking was almost uniformly decreased for severely ill neonates (8.4%-41.8% vs mild: 15.0%-66.7% and moderate: 30.5%-68.5%, p = 0.12- < 0.001), but multivariate analysis revealed that older age in all six African countries (AOR 1.11 [95% CI 1.02, 1.21], p = 0.02 to 1.10 [1.04, 1.16], p < 0.001) and moderate illness in three (4.83 [1.06, 21.96], p = 0.04 to 4.35 [1.59, 11.93], p = 0.005) were associated with careseeking, while severe illness was no longer significant. Similar to neonates, older age in three of five countries (1.26 [1.01, 1.58], p = 0.046 to 1.10 [1.03, 1.16], p = 0.003) and moderate illness in one (2.24 [1.17, 4.30], p = 0.016) were drivers of careseeking for 1–11-month-olds. Careseeking was increased in some countries for infectious illnesses but not for intrapartum- or prematurity-related conditions. Child mortality studies should assess severity level and caregivers’ response at various illness stages. Because older infants have more specific illness signs, the 2-sign method should be used only for neonates. Behavior change messages encouraging careseeking for moderate illness signs should be developed. The 2-sign method can serve as a practical tool for this purpose for illnesses of neonates. Effective interventions may require overcoming local barriers to careseeking and bringing delivery and newborn care closer to communities to prevent and treat early onset neonatal illnesses.

## Introduction

Caregivers’ timely seeking of appropriate health care for their children’s potentially fatal illnesses can make the difference between life and death. Study of the interplay of recognition of illness signs, perception of disease severity, and responsive careseeking might address this issue and provide fruitful insights into the development of effective interventions. Verbal autopsy (VA) is an interview-based methodology used for several years to determine cause of death in low resource settings where many or most child deaths occur outside of medical care. However, VA questionnaires typically have focused purely on identifying illness signs and symptoms, including severe symptoms such as severe cough and inability to suckle, and possibly mild or moderate symptoms such as rash and fever, but without any reference to careseeking and minimal inquiry about the timing of symptom occurrence. Only recently has the most prominent VA instrument included an open-ended narrative section that asks about respondent recognition of ‘symptoms’ and careseeking for the fatal illness [1].

A methodology that builds on and extends VA, called verbal and social autopsy (VASA), has addressed this issue by examining the timing of careseeking behavior relative to caregivers’ perception of the severity of their child’s illness [2–4]. A scale across severity levels, especially one based on signs and symptoms easily recognized by and of concern to child caregivers [5,6] and shown to have objective validity, such as activity level and feeding behavior [7–9], would be a boon to studies that aim to assess the contribution of illness perception and delayed careseeking to child mortality.

Such a tool could also contribute to the development of behavior change messages based on illness signs that should prompt careseeking before severe illness develops [10]. Some researchers have found that caregivers mainly respond to severe symptoms [11,12] or even fail to perceive the severity of their child’s illness [11] and that this leads to delayed careseeking and lessened chance for survival once reaching a health care provider [11,12]. However, others have found that mothers do respond to less severe symptoms [13], indicating the potential utility of such a tool.

The current paper develops sign-based methods of identifying mild, moderate, and severe conditions of the most vulnerable children, including neonates and 1–11-month-old infants, at the onset of a fatal illness. It then uses these methods to assess the relationship of caregivers’ perception of their child’s illness severity level to other variables affecting child mortality and the contribution of these factors to seeking formal health care for their sick neonates and infants.

## Materials and methods

This study was a secondary analysis of data from five national VASA studies conducted in Niger [2], Tanzania [3], Nigeria [14], Mozambique [15] and Pakistan, and three- and two-district VASA studies, respectively, in Cameroon (Doume, Nguelemendouka and Abong-Mbang districts) [16] and Malawi (Balaka and Salima districts) [17]. The Mozambique data were collected as part of a prospective national sample registration system with VASA, while the data for all other countries are from cross-sectional retrospective studies of deaths identified by a household survey (Niger, Tanzania, Nigeria, Malawi, Pakistan) or census (Cameroon). The years of the deaths/ conduct of VASA interviews (mean, median years interview recall period) in each country were: Cameroon 2006–10/ 2012 (3.2, 3), Nigeria 2008–13/ 2014 (3.7, 4), Malawi 2008–12/ 2013 (3.4, 3), Niger 2007–10/ 2012 (3.3, 3), Tanzania 2010–15/ 2017 (4.2, 4), Mozambique 2018–20/ 2018–20 (0.2, 0), and Pakistan 2015–19/ 2018–19 (1.5, 1). In five countries 13% to 42% of interviews were conducted more than four years after the death, while in Pakistan and Mozambique, respectively, 97% were conducted in less than four years and 100% in less than two years. The deceased child’s mother was the respondent for 77%, 96%, 87%, 94%, 93%, 86% and 91% of VASA interviews, respectively, in Cameroon, Nigeria, Malawi, Niger, Tanzania, Mozambique and Pakistan.

The VASA questionnaire used in all the African countries except Mozambique combined the Population Health Metrics Research Consortium (PHMRC) VA questionnaire [18] with the original Child Health Epidemiology Reference Group (CHERG) SA questionnaire [19]; while the VASA instrument in Pakistan and Mozambique incorporated the 2016 WHO VA questionnaire and an updated version of the CHERG SA questionnaire [20]. Survey weights were applied to the data from Malawi, Niger, Nigeria, Tanzania and Mozambique and all analyses were conducted such as to account for the multi-stage sampling designs of the platform surveys. Weights were not needed in Pakistan and neither weights nor correcting for the sampling design were required in Cameroon, where the deaths were identified through a population census of the study districts.

### Illness severity classification

We developed three methods using VA-identified signs and symptoms to classify illness severity during various stages of neonates’ and infants’ fatal illnesses; and compared the performance of the first method with the second and third methods in distinguishing mild, moderate and severe illness when first noted by the child’s caregiver (method 1) or on illness day-1 (methods 2 and 3). This comparison consisted of cross tabulating the method 1 findings in each country with those of methods 2 and 3, which utilize many more illness signs that method 1, and by examining the mean number of methods 2 and 3 urgent referral/severe illness signs corresponding with the method 1 mild, moderate, and severe illness classifications.

The first method consists of a simple 2-sign severity score based on reported activity level and feeding behavior (Panel 1). Caregivers were asked about these signs when they first noted the child was ill (defined as at onset), when it was decided to seek formal health care for the illness, and when the child left the first health provider alive. As a sensitivity test of whether the method is capable of identifying severe illness, hypothesizing that neonates who died from early onset illnesses such as perinatal asphyxia and very low birth weight would be more severely ill at onset, we compared the 2-sign severity scores of all neonates to those who died at age 0 or 1 day.

**Table:** 

**Panel 1. 2-sign method of classifying illness severity**				
**Individual**	**Levels and assigned scores**			
**Parameters**	**Normal = 1**	**Medium = 2**	**Abnormal = 3**	
Feeding	Normally	Poorly	Not at all	
Activity	Normal	Less active	Not moving	
**Combined scores and final analysis ranks**				
**Illness level**	**Signs**		**Score**	**Rank**
Mild	2 normal or 1 normal and 1 medium		2 3	1 1
Moderate	1 normal and 1 abnormal or 2 medium		4 4	2 2
Severe	1 medium and 1 abnormal or 2 abnormal		5 6	3 3

The second method examines for the presence of one or more of 15 neonatal and 13 child WHO/UNICEF Integrated Management of Childhood Illness (IMCI) signs of illness and severe illness requiring referral [21,22]; and the third method adds 18 neonatal signs and 16 child signs found only in the VASA formats (Panel 2). Caregivers were asked on which illness day, with the day of illness onset defined as day-1, each reported sign was first noted. For both methods 2 and 3, the absence of all the signs is classified as a mild illness, the presence only of other illness sign(s) identifies a moderate illness, and any urgent referral/severe illness sign(s) indicates a severe illness. Comparisons of the methods were not possible in Pakistan and Mozambique because the VASA questionnaire utilized in those countries did not collect the illness day of onset for each reported illness sign and symptom.

**Table:** 

**Panel 2. Integrated Management of Childhood Illness (IMCI) signs and Verbal and Social Autopsy (VASA) additional illness signs**	
Neonates (0–27 days old)*	
**IMCI signs requiring urgent referral**	**VASA additional signs of severe illness**
Not able to feed No movement at all Spasms or convulsions Fast breathing and age at death = 0–6 days Chest indrawing Fever Cold to touch Not able to suckle normally on first day of life Stopped being able to suckle normally Yellow skin or eyes and age at death = 0 days	Not able to breathe immediately after birth First cried >5 minutes after birth or never cried Stopped being able to cry Not able to open mouth when stopped suckling Ulcers/pits Areas of skin turned black Bleeding from anywhere Vomits everything Grunting Bulging fontanelle Umbilical cord stump redness extending to skin Unresponsive/unconscious
**IMCI other illness signs** ^**€**^	**VASA additional other illness signs**
Feeding poorly Pus drainage from umbilical cord stump Redness of umbilical cord stump Skin bumps containing pus or a single large area with pus Fast breathing and age at death = 7–27 days	Lethargic Bruises/signs of injury at birth Difficult breathing at birth First cried within 5 minutes after birth (did not cry immediately) 4 or more loose/watery stools on worst day Difficult breathing
Infants (1–11 months old)*	
IMCI signs requiring urgent referral**	**VASA additional signs of severe illness**
Not able to feed Convulsions Unconscious Stridor Stiff neck Limbs became very thin Swollen legs or feet	Severe cough Vomiting after coughing Grunting Wheezing Bulging fontanelle (in <18-month-olds) Skin flaked off in patches Hair changed to reddish or yellowish color Bleeding from anywhere Areas of skin turned black Injury (road traffic, fall, drowning, poisoning, bite/sting of venomous animal, burn, violence, other injury)
**IMCI other illness signs** ^**β**^	**VASA additional other illness signs**
Feeding poorly Fever Visible blood in loose or liquid stools Chest indrawing Fast breathing Pallor	3 or more loose/watery stools on the worst day Cough for 7 or more days Difficult breathing Blisters with clear fluid Swelling in the armpits Whitish rash in the mouth/on the tongue

*The actual IMCI age categories are ‘up to 2 months’ and ‘2 months up to 5 years;’ The current study utilizes the IMCI ‘up to 2 months’ signs to characterize neonatal illnesses, and the ‘2 months up to 5 years’ signs for 1–11-month-old illnesses; ^€^IMCI sign ‘Movement only when stimulated’ has no direct equivalent in the VASA questionnaire so is not included here. VASA’s ‘lethargic’ is similar in meaning and is included as a VASA additional other illness sign. **IMCI signs ’Severe persistent diarrhea with dehydration’ and ‘Diarrhea with severe dehydration’ cannot be identified using the VASA questionnaire due to missing questions for dehydration; ^β^IMCI signs ‘Diarrhea with some dehydration’ and’Persistent diarrhea without dehydration’ cannot be identified using the VASA questionnaire due to missing questions for dehydration

### Illness recognition and careseeking

The better-performing method in each age group was then used to assess caregivers’ perception of the child’s condition when illness that started in the community, i.e., for which the child’s caregiver could respond, was first noted or on illness day-1; and to examine its univariate associations with the child’s age at death and first seeking formal health care, defined as care provided by a health professional (doctor, nurse, trained health worker) in a hospital, health center, health post or private office, or by a trained community health worker, nurse or midwife. (Table A in S1 Appendix shows the percentage of neonatal and 1–11-month-old deaths whose illness started in the community, as opposed to illness that started in the delivery facility.) Multivariable logistic regression models were developed to examine factors associated with seeking formal health care (yes, no). Possible predictor variables included illness severity at onset or on illness day-1 (mild, moderate, severe), age at death (neonates: days; 1–11-month-olds: months), hours to reach the usually visited health facility in an emergency, the child’s mother’s age (years), the child’s mother’s completed years of formal schooling, and cause of death as determined by the Expert Algorithm (EAVA) analysis method [23] (neonates: severe infection, intrapartum related events [IPRE = birth asphyxia or injury] or prematurity, all other causes [reference value]; 1–11-month-olds: severe febrile infection, all other causes [reference value]). Neonatal severe infection included meningitis, pneumonia, and sepsis; 1–11-month-old severe febrile infection included AIDS, measles, meningitis, pertussis, pneumonia, malaria, hemorrhagic fever, and fever with rash, convulsions, or unconsciousness. We included diarrhea in the comparison group because it frequently presents without fever and formal careseeking for childhood diarrhea has been found to be strongly associated with fever [24]. SAS version 9.4 [25] was used to conduct all statistical analyses.

All analyses excluded cases missing caregiver’s perception of illness severity, as the primary variable of interest, or the outcome variable formal careseeking. Complete case (CC) multivariable analyses were conducted that also excluded cases missing any of the other predictor variables. Multivariable analyses were also done that substituted imputed mean (IM) values for missing travel time, mother’s age and mother’s schooling. There were no missing values for cause of death and child’s age. Complete case analyses are presented in the main paper for countries with less than 10% loss of data points due to missingness in at least one factor; otherwise, analyses using imputed means are presented [26].

### Ethics statement

Ethical clearance for the VASA study in each country was obtained from the Institutional Review Board of the Johns Hopkins Bloomberg School of Public Health (Cameroon: IRB No: 3868, Nigeria: IRB No: 5163, Malawi: IRB No: 2247, Niger: IRB No: 3274, Tanzania: IRB No: 7820, Mozambique: IRB No: 7867, Pakistan: IRB No: 8688) and the national ethics committee in each country, including the Cameroon National Ethics Committee (AUTORISATION N^O^285/CNE/SE/2011), the National Health Research Ethics Committee of the Nigeria Federal Ministry of Health (Approval Number NHREC/01/01/2007-30/10/2013), the Malawi National Health Sciences Research Committee (Approval number 927), the National Consultative Ethics Committee of the Niger Ministry of Health (DELIBERATION N0 014/2011/CCNE), the Tanzania National Institute for Medical Research (NIMR/HQ/R.8a/Vol.IX/2550) and the Ministry of Health of Zanzibar (PROTOCOL NO. ZAMREC/0001/JULY/17), the Mozambique National Health Bioethics Committee (REF 608/CNBS/17), and in Pakistan, the National Bioethics Committee (Ref: No.4-87/NBC-304/18/1026) and the Aga Khan University Ethics Review Committee (2018-0444-461). All respondents provided informed consent, either oral or written, depending on the country’s requirement and anticipated literacy of the respondents, before the VASA interview was conducted. Ethical review for the current analysis was not sought because all data are from the previously approved studies and were either de-identified early in the conduct of those studies or personal identifiers were encrypted at the time of data collection and indiscernible by the past and present studies’ authors. The data were accessed for the current analysis from February 11, 2023 – February 22, 2025.

## Results

### Study population

The full sample sizes of neonates in the seven countries ranged from 164 in Cameroon to 2,088 in Pakistan, with those whose illness started at home ranging from 115 in Tanzania to 1,381 in Pakistan. The number of 1–11-month-olds ranged from 158 in Tanzania to 691 in Nigeria (Table A in S1 Appendix).

Tables 1 a and 1b present some demographic characteristics of the study population. Two-thirds to 85% of neonatal deaths were of early neonates, while infant deaths averaged about six months of age. Table 4 provides additional information on neonates’ and 1–11-month-olds’ age relative to their illness severity at onset. Males predominated among neonatal deaths, but infant deaths were nearly equally distributed among males and females.

**Table 1:** a. Selected demographic characteristics of neonates in seven study countries. b. Selected demographic characteristics of 1-11-month-olds in five study countries.

Characteristic	Cameroon	Nigeria	Malawi	Niger	Tanzania	Mozambique	Pakistan
	N (%)	N (%)	N (%)	N (%)	N (%)	N (%)	N (%)	
Child’s age (days)							
0-6	116 (70.7)	535 (74.0)	224 (70.0)	311 (68.7)	195 (85.5)	302 (74.9)	1,629 (78.0)
7-27	48 (29.3)	188 (26.0)	96 (30.0)	142 (31.3)	33 (14.5)	101 (25.1)	459 (22.0)
μ (SE) days	4.6 (0.7)	4.5 (0.2)	5.1 (0.4)	6.1 (0.3)	3.1 (0.4)	4.5 (0.3)	4.3 (0.1)
Sex							
Male	96 (58.5)	421 (58.2)	183 (57.2)	264 (58.3)	136 (59.6)	210 (54.8)	1282 (61.4)
Female	68 (41.5)	301 (41.6)	137 (42.8)	189 (41.7)	92 (40.4)	173 (45.2)	806 (38.6)
Don’t know	0 (0)	1 (0.1)	0 (0)	0 (0)	0 (0)	20 (5.0)	0 (0)
Birthplace							
Home	92 (56.1)	418 (57.8)	81 (25.3)	313 (69.1)	53 (23.2)	163 (40.4)	619 (29.6)
Hospital	52 (31.7)	172 (23.8)	119 (37.2)	26 (5.7)	109 (47.8)	107 (26.6)	1,165 (55.8)
Other facility	12 (7.3)	78 (10.8)	96 (30.0)	102 (22.5)	51 (22.4)	103 (25.6)	283 (13.6)
Other	8 (4.9)	55 (7.6)	24 (7.5)	12 (2.6)	15 (6.6)	30 (7.4)	18 (0.9)
Don’t know	0 (0)	0 (0)	0 (0)	0 (0)	0 (0)	0 (0)	3 (0.1)
Place of death							
Home	93 (56.7)	478 (66.1)	122 (38.1)	349 (77.0)	68 (29.8)	221 (54.8)	799 (38.3)
Hospital	50 (30.5)	140 (19.4)	119 (37.2)	29 (6.4)	109 (47.8)	123 (30.5)	1,154 (55.3)
Other facility	5 (3.0)	55 (7.6)	55 (17.2)	51 (11.3)	35 (15.4)	32 (7.9)	48 (2.3)
Other	16 (9.8)	49 (6.8)	24 (7.5)	24 (5.3)	16 (7.0)	27 (6.7)	86 (4.1)
Don’t know	0 (0)	1 (0.1)	0 (0)	0 (0)	0 (0)	0 (0)	1 (0.05)
Mother’s age							
10-Dec	6 (3.7)	0 (0)	0 (0)	0 (0)	0 (0)	0 (0)	0 (0)
13-19	61 (37.2)	150 (20.7)	83 (25.9)	96 (21.2)	52 (22.8)	127 (31.5)	276 (13.2)
20-34	75 (45.7)	464 (64.2)	196 (61.3)	275 (60.7)	133 (58.3)	228 (56.6)	1,537 (73.6)
35+	17 (10.4)	106 (14.7)	34 (10.6)	63 (13.9)	41 (18.0)	45 (11.2)	246 (11.8)
Missing	5 (3.0)	3 (0.4)	7 (2.2)	19 (4.2)	2 (0.9)	3 (0.7)	29 (1.4)
μ (SE) years	22.7 (0.6)	26.5 (0.3)	24.5 (0.4)	25.5 (0.3)	26.2 (0.5)	24.7 (0.4)	26.5 (0.1)
Mother’s education							
None	4 (2.4)	350 (48.4)	46 (14.4)	382 (84.3)	30 (13.2)	7 (1.7)	2 (0.1)
Primary	108 (65.9)	168 (23.2)	181 (56.6)	49 (10.8)	39 (17.1)	198 (49.1)	329 (15.8)
Secondary	50 (30.5)	162 (22.4)	91 (28.4)	14 (3.1)	151 (66.2)	90 (22.3)	358 (17.1)
Higher	2 (1.2)	28 (3.9)	1 (0.3)	1 (0.2)	8 (3.5)	0 (0)	72 (3.4)
Missing	0 (0)	15 (2.1)	1 (0.3)	7 (1.5)	0 (0)	108 (26.8)	1,327 (63.6)
HH* electricity							
Yes	42 (25.6)	315 (43.6)	11 (3.4)	39 (8.6)	49 (21.5)	82 (20.3)	1,983 (95.0)
No	122 (74.4)	408 (56.4)	309 (96.6)	414 (91.4)	179 (78.5)	321 (79.7)	105 (5.0)
HH* floor material							
Mud/clay	132 (80.5)	246 (34.0)	290 (90.6)	416 (91.8)	134 (58.8)	320 (79.4)	898 (43.0)
W/C/T**	32 (19.5)	442 (61.1)	29 (9.1)	36 (7.9)	94 (41.2)	83 (20.6)	1,159 (55.0)
Other	0 (0)	35 (4.8)	1 (0.3)	1 (0.2)	0 (0)	0 (0)	30 (1.4)
Don’t know	0 (0)	0 (0)	0 (0)	0 (0)	0 (0)	0 (0)	1 (0.05)
Child’s age (months)							
1-2	33 (12.6)	159 (23.0)	69 (20.6)	60 (22.3)	41 (25.9)		
3-6	96 (36.8)	228 (33.0)	115 (34.3)	91 (33.8)	50 (31.6)		
7-11	132 (50.6)	304 (44.0)	151 (45.1)	118 (43.9)	67 (42.4)		
μ (SE) months	6.5 (0.2)	5.8 (0.1)	5.8 (0.2)	5.6 (0.2)	5.5 (0.3)		
Sex							
Male	136 (52.1)	350 (50.7)	166 (49.6)	137 (50.9)	81 (51.3)		
Female	125 (47.9)	341 (49.3)	169 (50.4)	132 (49.1)	77 (48.7)		
Birthplace							
Home	180 (69.0)	431 (62.4)	54 (16.1)	195 (72.5)	50 (31.6)		
Hospital	45 (17.2)	156 (22.6)	153 (45.7)	7 (2.6)	58 (36.7)		
Other facility	33 (12.6)	79 (11.4)	106 (31.6)	62 (23.0)	44 (27.8)		
Other	3 (1.1)	25 (3.6)	22 (6.6)	5 (1.9)	6 (3.8)		
Place of death							
Home	146 (55.9)	484 (70.0)	155 (46.3)	215 (79.9)	65 (41.1)		
Hospital	51 (19.5)	127 (18.4)	119 (35.5)	18 (6.7)	53 (33.5)		
Other facility	19 (7.3)	30 (4.3)	28 (8.4)	20 (7.4)	13 (8.2)		
Other	45 (17.2)	49 (7.1)	33 (9.9)	15 (5.6)	27 (17.1)		
Don’t know	0 (0)	1 (0.1)	0 (0)	1 (0.4)	0 (0)		
Mother’s age							
10-Dec	2 (0.8)	1 (0.1)	0 (0)	0 (0)	0 (0)		
13-19	85 (32.6)	103 (14.9)	54 (16.1)	37 (13.8)	28 (17.7)		
20-34	138 (52.9)	463 (67.0)	217 (64.8)	174 (64.7)	90 (57.0)		
35+	29 (11.2)	120 (17.4)	48 (14.3)	37 (13.8)	37 (23.4)		
Missing	7 (2.7)	4 (0.6)	16 (4.8)	21 (7.8)	3 (1.9)		
μ (SE) years	23.9 (0.5)	27.1 (0.3)	26.8 (0.4)	26.9 (0.5)	27.6 (0.6)		
Mother’s education							
None	4 (1.5)	337 (48.8)	76 (22.7)	233 (86.6)	30 (19.0)		
Primary	190 (72.8)	164 (23.7)	184 (54.9)	23 (8.6)	24 (15.2)		
Secondary	63 (24.1)	129 (18.7)	72 (21.5)	7 (2.6)	104 (65.8)		
Higher	0 (0)	40 (5.8)	2 (0.6)	0 (0)	0 (0)		
Missing	4 (1.5)	21 (3.0)	1 (0.3)	6 (2.2)	0 (0)		
HH* electricity							
Yes	69 (26.4)	305 (44.1)	3 (0.9)	24 (8.9)	26 (16.5)		
No	192 (73.6)	386 (55.9)	332 (99.1)	245 (91.1)	132 (83.5)		
HH* floor material							
Mud/clay	218 (83.5)	241 (34.9)	295 (88.1)	244 (90.7)	108 (68.4)		
W/C/T**	43 (16.5)	422 (61.1)	40 (11.9)	25 (9.3)	50 (31.6)		
Other	0 (0)	28 (4.1)	0 (0)	0 (0)	0 (0)		

*Household, **Wood/cement/tiles

Home births and deaths were common, most markedly in Nigeria and Niger, with nearly 80% of deaths in Niger occurring at home. This appeared to correlate with a lack of maternal education, with nearly half to about 85% of mothers in these countries having received no schooling. However, there did not seem to be an association with mother’s young age, as these two countries had the lowest proportions of teenage mothers except for mothers of neonates in Pakistan. Table 5 shows mothers’ mean ages and years of schooling stratified by formal careseeking for their child’s illness.

The high percentages of households in all countries but Pakistan without electricity and with mud or clay floors suggests the extreme poverty of the study households. However, Nigeria, with the second highest level of home deaths, had the highest level of electricity and lowest of mud or clay floors among all the African countries, while also having the second highest level of no maternal education.

### Illness severity classification

Tables 2 and 3 show the association between the 2-sign classification of illness severity at onset and the IMCI and extended IMCI-VASA classifications on illness day-1 for neonatal and 1–11-month-old deaths, respectively. It can be seen in Table 2 that both the IMCI and IMCI-VASA methods classified 82% to 96% of fatal neonatal illnesses as severe on illness day-1; whereas the 2-sign method classified 38% to 61% of all illnesses as severe, 19% to 53% as moderate, and 9% to 24% as mildly ill. Weighted Cohen’s Kappa statistic confirmed very low agreement between the 2-sign and corresponding IMCI and IMCI-VASA categories, with Kappas of 0.42 and 0.16 for Tanzania and ranging from 0.01 to 0.31 for the other four countries (Table B in S1 Appendix). Also, among neonates with a severe illness by the IMCI and IMCI-VASA methods, Table 2 reveals a uniformly upward trend in the mean number, respectively, of IMCI urgent referral and IMCI-VASA severe illness signs for neonates with an illness classified by the 2-sign method as mild, moderate, and severe.

**Table 2:** Association of VASA 2-sign illness severity score with IMCI illness signs requiring treatment and urgent referral and IMCI illness signs plus additional VASA illness signs, and the mean number of IMCI and IMCI-VASA illness signs that led to urgent referral and severe illness assessments, for neonates whose fatal illness started in the community.

Country	VASA 2-sign severity at illness onset	IMCI day-1 illness severity				IMCI-VASA day-1 illness signs				Total N (%)*
		Mild illness N (%)*	Moderate illness N (%)*	Urgent referral		Mild illness N (%)*	Moderate illness N (%)*	Severe illness		
				N (%)*	μ # of signs			N (%)*	μ # of signs	
**Cameroon**	**Mild**	6 (100)	1 (10)	11 (8.9)	1.364	0	2 (40.0)	16 (11.9)	1.813	18 (13.0)
	**Moderate**	0	9 (90)	27 (22.0)	2.111	0	3 (60.0)	33 (24.6)	3.121	36 (25.9)
	**Severe**	0	0	85 (69.1)	3.306	0	0	85 (63.4)	5.012	85 (61.1)
	Total**	6 (4.3)	10 (7.2)	123 (88.5)		0	5 (3.6)	134 (96.4)		139 (100)
**Nigeria**	**Mild**	29 (100)	3 (4.7)	17 (3.7)	1.455	23 (100)	6 (13.1)	20 (4.1)	1.592	48 (8.8)
	**Moderate**	0	57 (95.3)	232 (50.6)	2.378	0	39 (86.9)	250 (52.2)	2.923	289 (52.9)
	**Severe**	0	0	209 (45.7)	3.451	0	0	209 (43.6)	5.015	209 (38.3)
	Total**	29 (5.3)	60 (11.0)	457 (83.7)		23 (4.2)	44 (8.1)	479 (87.7)		546 (100)
**Malawi**	**Mild**	11 (100)	2 (9.2)	29 (17.6)	1.647	5 (100)	2 (12.5)	36 (19.8)	1.668	43 (21.0)
	**Moderate**	0	24 (90.8)	56 (33.4)	2.046	0	14 (87.5)	65 (35.5)	2.492	79 (38.9)
	**Severe**	0	0	82 (49.1)	3.403	0	0	82 (44.7)	3.403	82 (40.1)
	Total**	11 (5.4)	26 (12.8)	167 (81.7)		5 (2.2)	16 (8.0)	183 (89.7)		204 (100)
**Niger**	**Mild**	21 (100)	2 (9.9)	39 (10.3)	2.264	6 (100)	4 (26.1)	51 (13.0)	2.877	61 (14.7)
	**Moderate**	0	14 (90.1)	109 (28.7)	2.618	0	10 (73.9)	113 (28.5)	3.567	123 (29.6)
	**Severe**	0	0	232 (61.0)	3.340	0	0	232 (58.5)	4.800	232 (55.7)
	Total**	21 (5.0)	16 (3.8)	379 (91.3)		6 (1.4)	14 (3.4)	396 (95.2)		416 (100)
**Tanzania**	**Mild**	15 (100)	1 (22.4)	11 (11.4)	2.179	7 (100)	2 (38.9)	18 (17.5)	2.508	26 (23.5)
	**Moderate**	0	3 (77.6)	18 (19.3)	2.300	0	2 (61.1)	19 (18.8)	3.496	21 (19.1)
	**Severe**	0	0	65 (69.3)	3.571	0	0	65 (63.8)	5.197	65 (57.4)
	Total**	15 (13.1)	4 (4.0)	93 (82.9)		7 (6.4)	4 (3.5)	101 (90.1)		112 (100)

*column percent; **row percent

**Table 3:** Association of VASA 2-sign illness severity score with IMCI illness signs requiring treatment and urgent referral and IMCI illness signs plus additional VASA illness signs, and the mean number of IMCI and IMCI-VASA illness signs that led to urgent referral and severe illness assessments, for 1-11-month-olds whose fatal illness started in the community.

Country	VASA 2-sign severity at illness onset	IMCI day-1 illness severity				IMCI-VASA day-1 illness signs				Total N (%)*
		Mild illness N (%)*	Moderate illness N (%)*	Urgent referral		Mild illness N (%)*	Moderate illness N (%)*	Severe illness		
				N (%)*	μ # of signs			N (%)*	μ # of signs	
**Cameroon**	**Mild**	81 (94.2)	21 (19.1)	6 (9.2)	1.667	48 (94.1)	34 (32.4)	26 (24.8)	1.192	108 (41.4)
	**Moderate**	5 (5.8)	85 (77.3)	14 (21.5)	1.286	3 (5.9)	67 (63.8)	34 (32.4)	1.382	104 (39.9)
	**Severe**	0	4 (3.6)	45 (69.2)	1.422	0	4 (3.8)	45 (42.9)	1.614	49 (18.8)
	Total**	86 (33.0)	110 (42.1)	65 (24.9)		51 (19.5)	105 (40.2)	105 (40.2)		261 (100)
**Nigeria**	**Mild**	84 (98.1)	11 (2.3)	5 (4.7)	1.414	65 (97.6)	18 (4.0)	18 (10.0)	1.295	100 (14.6)
	**Moderate**	2 (1.9)	469 (95.4)	39 (34.0)	1.143	2 (2.4)	423 (94.6)	84 (47.9)	1.301	509 (73.8)
	**Severe**	0	11 (2.2)	70 (61.2)	1.232	0	6 (1.4)	74 (42.1)	1.477	80 (11.7)
	Total**	85 (12.3)	491 (71.2)	114 (16.5)		67 (9.7)	447 (64.8)	176 (25.5)		690^β^ (100)
**Malawi**	**Mild**	90 (97.9)	19 (13.0)	24 (23.8)	1.205	63 (98.8)	38 (28.4)	32 (23.1)	1.662	132 (39.5)
	**Moderate**	2 (2.1)	120 (83.6)	19 (19.1)	1.249	1 (1.2)	94 (70.3)	46 (33.2)	1.392	141 (42.0)
	**Severe**	0	5 (3.4)	57 (57.0)	1.449	0	2 (1.3)	60 (43.7)	1.986	62 (17.9)
	Total**	92 (27.5)	143 (42.8)	100 (29.8)		63 (18.9)	134 (40.1)	137 (41.0)		335 (100)
**Niger**	**Mild**	36 (93.2)	16 (12.6)	21 (20.7)	1.107	21 (91.2)	20 (17.1)	33 (25.3)	1.279	74 (27.4)
	**Moderate**	3 (6.8)	100 (79.2)	13 (12.5)	1.092	2 (8.8)	87 (74.0)	27 (20.8)	1.138	115 (42.9)
	**Severe**	0	10 (8.2)	69 (66.9)	1.425	0	10 (8.9)	69 (53.9)	1.727	80 (29.7)
	Total**	39 (14.5)	126 (46.9)	104 (38.6)		23 (8.6)	117 (43.5)	129 (47.9)		269 (100)
**Tanzania**	**Mild**	51 (98.2)	10 (16.1)	12 (30.0)	1.100	28 (96.9)	15 (27.3)	30 (40.5)	1.520	73 (46.6)
	**Moderate**	1 (1.8)	52 (80.3)	5 (12.4)	1.663	1 (3.1)	35 (68.1)	22 (28.8)	1.407	58 (36.9)
	**Severe**	0	2 (3.7)	24 (57.6)	1.544	0	2 (4.6)	24 (30.7)	2.249	26 (16.5)
	Total**	52 (32.8)	65 (41.2)	41 (26.0)		29 (18.5)	53 (33.4)	76 (48.1)		157^β^ (100)

*column percent; **row percent; ^β^1 missing due to missing values for feeding behavior and activity level

The sensitivity test of the 2-sign method was conducted among the 45%, 33%, 32%, 25%, and 54% of all neonates who died on day-0 or day-1 of life, respectively, in Cameroon, Nigeria, Malawi, Niger, and Tanzania. While 61%, 38%, 40%, 56%, and 57% of all neonates in these countries were classified by the 2-sign method as being severely ill at onset (Table 2), 84%, 71%, 69%, 75%, and 81% of neonates that died on day-0 or day-1 were classified as having a severe illness.

Table 3 shows that the IMCI and IMCI-VASA illness signs classified more illnesses of 1–11-month-olds on illness day-1 as mild (9% to 33%) or moderate (33% to 71%) than they did for neonatal illnesses (Table 2). Other than for Tanzania, with Kappas of 0.29 and 0.37, there was moderate to strong agreement of from 0.43 to 0.78 between the 2-sign and corresponding IMCI and IMCI-VASA categories (Table B in S1 Appendix). The 2-sign method also did not as clearly or uniformly separate IMCI and IMCI-VASA severe illnesses characterized by fewer and more signs of IMCI urgent referral and IMCI-VASA severe illness as it did with neonatal illnesses.

We examined caregivers’ response to recognition of neonates’ moderate and severe illnesses based on the 2-sign method of classifying illness severity, and the response to 1–11-month-old infants’ illnesses based on the IMCI-VASA classification method. The IMCI-VASA method was selected over the IMCI alone method because it includes additional signs whose presence indicate a moderate or severe illness, and it identified more illnesses as severe at onset in all five countries. Due to the above-noted inability of the VASA questionnaire utilized in Mozambique and Pakistan to implement the IMCI-VASA classification method, caregivers’ response to illness severity in those two countries was examined only for illnesses of neonates.

### Utility of the illness-severity score for examining careseeking

**Fig 1:**
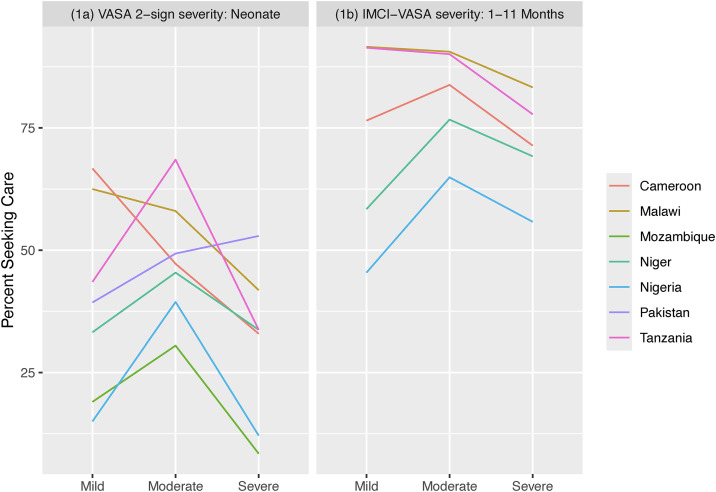
Percent of neonates (1a) and 1–11-month-olds (1b) with a mild, moderate and severe illness at onset or on illness day-1, respectively, by the VASA 2-sign and IMCI-VASA illness severity methods, who sought formal health care.

**Fig 2:**
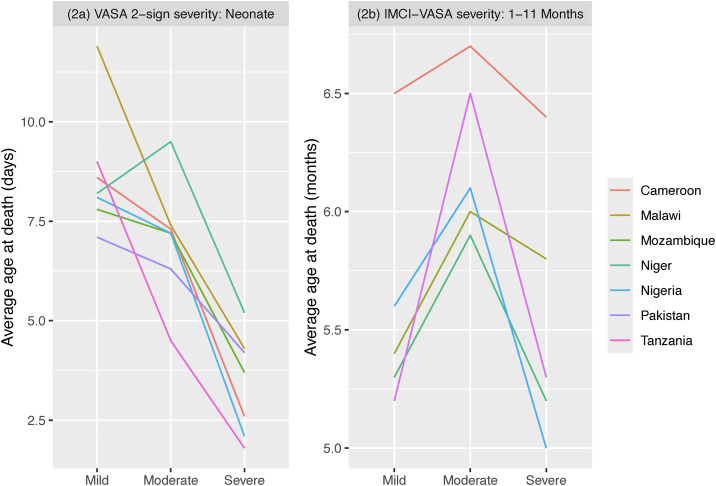
The average age at death of neonates (2a) and 1–11-month-olds (2b) with a mild, moderate and severe illness at onset or on illness day-1, respectively, by the VASA 2-sign and IMCI-VASA illness severity methods.

Table 4 shows a significant negative association between illness severity and formal careseeking for neonates with a fatal illness in two of the six African countries, and an apparent negative or near-negative association in three other countries that is punctuated by greater careseeking for moderate than either mild or severe illness (Fig 1a). There was also a negative association between age and illness severity in these five countries. In Pakistan the opposite relationship was found between formal careseeking and illness severity—there was a significant positive association; while, just as in the African countries, illness severity increased with decreasing age at death (Table 4 and Fig 2a).

**Table 4:** Association of perceived severity at illness onset or illness day-1 with age at death and formal careseeking for fatal illnesses, respectively, of neonates and 1-11-month-olds that started in the community^Ω.^

	Neonates							1-11-month-olds						
Country	VASA 2-sign severity at illness onset	Total N (%)*	Age at death μ days (SE)	F^€^ P-value	Did not seek formal care^£^ N (%)**	Sought formal care^£^ N (%)**	X^2^ P-value	IMCI-VASA day-1 illness signs	Total N (%)*	Age at death μ months (SE)	F^€^ P-value	Did not seek formal care^£^ N (%)**	Sought formal care^£^ N (%)**	X^2^ P-value
**Cameroon**	**Mild**	18 (13.0)	8.6 (1.2)	18.40 <0.001	6 (33.3)	12 (66.7)	7.76^β^ 0.021	**Mild**	51 (19.5)	6.5 (0.4)	0.28 0.754	12 (23.5)	39 (76.5)	4.63^β^ 0.099
	**Moderate**	36 (25.9)	7.3 (1.1)		19 (52.8)	17 (47.2)		**Moderate**	105 (40.2)	6.7 (0.3)		17 (16.2)	88 (83.8)	
	**Severe**	85 (61.1)	2.6 (0.5)		57 (67.1)	28 (32.9)		**Severe**	105 (40.2)	6.4 (0.3)		30 (28.6)	75 (71.4)	
	**Total**	139 (100)	4.6 (0.5)		82 (59.0)	57 (41.0)		**Total**	261 (100)	6.5 (0.2)		59 (22.6)	202 (77.4)	
**Nigeria**	**Mild**	48 (8.8)	8.1 (0.8)	62.51 <0.001	41 (85.0)	7 (15.0)	43.27 <0.001	**Mild**	68 (9.8)	5.6 (0.5)	2.70 0.068	37 (54.6)	31 (45.4)	9.50 0.009
	**Moderate**	289 (52.9)	7.2 (0.3)		175 (60.6)	114 (39.4)		**Moderate**	447 (64.7)	6.1 (0.2)		157 (35.1)	290 (64.9)	
	**Severe**	209 (38.3)	2.1 (0.3)		184 (87.9)	25 (12.1)		**Severe**	176 (25.5)	5.0 (0.3)		78 (44.2)	98 (55.8)	
	**Total**	546 (100)	5.3 (0.3)		400 (73.2)	146 (26.8)		**Total**	691 (100)	5.7 (0.1)		272 (39.4)	419 (60.6)	
**Malawi**	**Mild**	43 (21.0)	11.9 (1.3)	15.61 <0.001	16 (37.5)	27 (62.5)	6.31 0.043	**Mild**	63 (18.9)	5.4 (0.4)	1.30 0.275	5 (8.4)	58 (91.6)	4.02 0.134
	**Moderate**	79 (38.9)	7.4 (0.9)		33 (42.0)	46 (58.0)		**Moderate**	134 (40.1)	6.0 (0.3)		13 (9.4)	122 (90.6)	
	**Severe**	82 (40.1)	4.3 (0.8)		48 (58.2)	34 (41.8)		**Severe**	137 (41.0)	5.8 (0.3)		23 (16.7)	114 (83.3)	
	**Total**	204 (100)	7.1 (0.6)		97 (47.6)	107 (52.4)		**Total**	335 (100)	5.8 (0.2)		41 (12.2)	294 (87.8)	
**Niger**	**Mild**	61 (14.7)	8.2 (0.9)	17.74 <0.001	41 (66.8)	20 (33.2)	4.21 0.122	**Mild**	23 (8.6)	5.3 (0.7)	0.46 0.634	10 (41.6)	13 (58.4)	2.62 0.270
	**Moderate**	123 (29.6)	9.5 (0.9)		67 (54 6)	56 (45.4)		**Moderate**	117 (43.5)	5.9 (0.3)		27 (23.3)	90 (76.7)	
	**Severe**	232 (55.7)	5.2 (0.5)		154 (66.3)	78 (33.7)		**Severe**	129 (47.9)	5.2 (0.3)		40 (30.8)	89 (69.2)	
	**Total**	416 (100)	6.9 (0.4)		262 (62.9)	154 (37.1)		**Total**	269 (100)	5.5 (0.2)		77 (28.5)	192 (71.5)	
**Tanzania**	**Mild**	26 (23.5)	9.0 (1.6)	11.51 <0.001	15 (56.5)	11 (43.5)	5.76 0.056	**Mild**	30 (18.8)	5.2 (0.7)	2.23 0.111	3 (8.6)	27 (91.4)	5.62 0.060
	**Moderate**	21 (19.1)	4.5 (1.0)		7 (31.5)	15 (68.5)		**Moderate**	53 (33.3)	6.5 (0.4)		5 (9.9)	47 (90.1)	
	**Severe**	65 (57.4)	1.8 (0.4)		43 (66.3)	22 (33.7)		**Severe**	76 (47.9)	5.3 (0.5)		17 (22.2)	59 (77.8)	
	**Total**	112 (100)	4.0 (0.6)		64 (57.4)	48 (42.6)		**Total**	158 (100)	5.7 (0.3)		25 (15.5)	133 (84.5)	
**Mozambique**	**Mild**	105 (39.4)	7.8 (0.8)	7.67 0.001	85 (81.0)	20 (19.0)	11.64 0.003							
	**Moderate**	66 (24.7)	7.2 (0.8)		46 (69.5)	20 (30.5)								
	**Severe**	95 (35.8)	3.7 (0.6)		87 (91.6)	8 (8.4)								
	**Total**	265 (100)	6.2 (0.5)		217 (82.0)	48 (18.0)								
**Pakistan**	**Mild**	267 (21.8)	7.1 (0.4)	23.86 <0.001	162 (60.7)	105 (39.3)	14.42 <0.001							
	**Moderate**	298 (24.3)	6.3 (0.4)		151 (50.7)	147 (49.3)								
	**Severe**	662 (54.0)	4.2 (0.2)		312 (47.1)	350 (52.9)								
	**Total**	1227 (100)	5.4 (0.2)		625 (50.9))	602 (49.1)								

^Ω^25, 177, 116, 37, 116, 137, and 861 neonatal deaths started in the delivery hospital, respectively, in Cameroon, Nigeria, Malawi, Niger, Tanzania, Mozambique, and Pakistan; Total Ns for neonates also exclude cases missing data for illness severity or formal careseeking (see Table C in S1 Appendix); *column percent; ^€^Anova; ^£^Did not seek formal care at any time during the illness, and Sought formal care during the illness; **row percent; ^β^Pearson chi-square (all other chi-squares are Rao-Scott)

The association of illness severity with careseeking was less definitive for 1–11-month-olds than for neonates. Only in Tanzania was there a (nearly) significant negative association between illness severity and formal careseeking; while in Nigeria, like the finding for neonates, careseeking was greater for moderately ill than either mildly or severely ill children. This was also true in Cameroon and Niger but not significantly so (Table 4 and Fig 1b). Also, contrary to the finding for neonates, there was no significant association between age and illness severity, though moderately ill children were uniformly somewhat older than either the mildly or severely ill (Table 4 and Fig 2b).

Based on the < 10%/ ≥ 10% missing data points criteria, Table 5 presents the results of multivariable logistic regression IM analyses for Mozambique and Pakistan and CC analyses for all other countries. Tables C-F in S1 Appendix present the percentage of missing values for all variables, and Table G in S1 Appendix provides the CC analyses for Mozambique and Pakistan and IM analyses for all other countries. The Table 5 results are favored due to the low level of missing data in all countries but Mozambique and Pakistan.

**Table 5:** Multivariable logistic regression of factors associated with seeking formal health care for neonates and 1-11-month-olds with a fatal illness^Ω.^

Country Explanatory factors	Neonates (0–27 days)				1-11-month-olds			
	Did not seek formal care^£^ N (%)	Sought formal care^£^ N (%)	p-value*	AOR (95% CI)	Did not seek formal care^£^ N (%)	Sought formal care^£^ N (%)	p-value*	AOR (95% CI)
**Cameroon**	79 (59.4)	54 (40.6)			54 (22.1)	190 (77.9)		
Neonatal cause of death								
All other causes	11 (13.9)	5 (9.3)	--	1.0 (ref^β^)	--	--	--	--
IPRE^€^ or prematurity	46 (58.2)	22 (40.7)	0.731	1.26 (0.34, 4.61)	--	--	--	--
Severe infection	22 (27.9)	27 (50.0)	0.134	2.69 (0.74, 9.84)	--	--	--	--
Infant cause of death								
All other causes	--	--	--	--	21 (38.9)	70 (36.8)	--	1.0 (ref)
Severe febrile infection	--	--	--	--	33 (61.1)	120 (63.2)	0.698	1.14 (0.60, 2.15)
Neonatal age at death Mean days (SE**)	3.04 (0.47)	6.37 (0.86)	0.017	1.11 (1.02, 1.21)	--	--	--	--
Infant age at death Mean months (SE)	--	--	--	--	5.59 (0.43)	6.77 (0.22)	0.024	1.13 (1.02, 1.25)
Illness severity^±^								
Mild	6 (7.6)	11 (20.4)	--	1.0 (ref)	12 (22.2)	38 (20.0)	--	1.0 (ref)
Moderate	19 (24.1)	17 (31.5)	0.288	0.50 (0.14, 1.80)	15 (27.8)	80 (42.1)	0.319	1.55 (0.66, 3.67)
Severe	54 (68.4)	26 (48.2)	0.154	0.40 (0.11, 1.41)	27 (50.0)	72 (37.9)	0.667	0.84 (0.37, 1.88)
Travel to usual facility Mean hours (SE)	0.70 (0.09)	0.58 (0.08)	0.768	0.92 (0.54, 1.59)	0.78 (0.10)	0.81 (0.06)	0.486	1.15 (0.78, 1.70)
Mother’s age Mean years (SE)	22.32 (0.79)	22.09 (1.13)	0.959	1.00 (0.95, 1.05)	24.93 (1.05)	23.72 (0.52)	0.416	0.98 (0.94, 1.02)
Mother’s education Mean years (SE)	5.82 (0.23)	6.07 (0.27)	0.036	1.25 (1.02, 1.54)	5.17 (0.26)	5.41 (0.15)	0.773	1.02 (0.87, 1.20)
**Nigeria**	381 (72.6)	144 (27.4)			254 (38.6)	404 (61.4)		
Neonatal cause of death								
All other causes	101 (26.4)	30 (20.9)	--	1.0 (ref)	--	--	--	--
IPRE or prematurity	107 (28.0)	18 (12.7)	0.827	0.91 (0.38, 2.17)	--	--	--	--
Severe infection	174 (45.6)	95 (66.4)	0.086	1.66 (0.93, 2.96)	--	--	--	--
Infant cause of death								
All other causes	--	--	--	--	86 (33.9)	118 (29.1)	--	1.0 (ref)
Severe febrile infection	--	--	--	--	168 (66.1)	287 (70.9)	0.019	1.60 (1.08, 2.36)
Neonatal age at death Mean days (SE)	4.28 (0.28)	7.60 (0.52)	0.001	1.07 (1.03, 1.11)	--	--	--	--
Infant age at death Mean months (SE)	--	--	--	--	5.20 (0.20)	5.94 (0.17)	0.003	1.10 (1.03, 1.16)
Illness severity^±^								
Mild	40 (10.5)	7 (4.6)	--	1.0 (ref)	35 (13.6)	31 (7.6)	--	1.0 (ref)
Moderate	164 (42.9)	112 (77.8)	0.005	4.35 (1.59, 11.93)	143 (56.3)	278 (68.8)	0.016	2.24 (1.17, 4.30)
Severe	177 (46.0)	25 (17.6)	0.520	1.46 (0.46, 4.60)	76 (30.1)	95 (23.6)	0.200	1.57 (0.79, 3.11)
Travel to usual facility Mean hours (SE)	0.89 (0.05)	0.83 (0.07)	0.528	0.89 (0.61, 1.29)	0.93 (0.05)	0.90 (0.17)	0.672	0.97 (0.86, 1.11)
Mother’s age Mean years (SE)	25.84 (0.39)	26.99 (0.67)	0.231	1.02 (0.99, 1.06)	25.84 (0.49)	27.62 (0.38)	0.045	1.03 (1.00, 1.05)
Mother’s education Mean years (SE)	2.50 (0.22)	4.03 (0.39)	0.004	1.08 (1.03, 1.14)	2.18 (0.22)	5.00 (0.26)	<0.001	1.15 (1.10, 1.21)
**Malawi**	93 (46.8)	105 (53.2)			35 (11.1)	280 (88.9)		
Neonatal cause of death								
All other causes	31 (33.5)	19 (17.8)	--	1.0 (ref)	--	--	--	--
IPRE or prematurity	29 (31.0)	24 (22.4)	0.105	2.20 (0.85, 5.72)	--	--	--	--
Severe infection	33 (35.5)	63 (59.8)	0.017	2.73 (1.20, 6.18)	--	--	--	--
Infant cause of death								
All other causes	--	--	--	--	19 (54.7)	95 (34.0)	--	1.0 (ref)
Severe febrile infection	--	--	--	--	16 (45.3)	184 (66.0)	0.014	2.73 (1.23, 6.06)
Neonatal age at death Mean days (SE)	4.39 (0.64)	9.47 (0.89)	0.001	1.10 (1.04, 1.16)	--	--	--	--
Infant age at death Mean months (SE)	--	--	--	--	5.04 (0.57)	5.92 (0.20)	0.128	1.11 (0.97, 1.27)
Illness severity^±^								
Mild	16 (17.4)	27 (25.4)	0.460	1.0 (ref)	4 (12.8)	55 (19.7)	--	1.0 (ref)
Moderate	33 (35.3)	45 (42.7)	0.771	1.42 (0.56, 3.57)	12 (33.9)	113 (40.5)	0.911	1.08 (0.30, 3.81)
Severe	44 (47.3)	34 (31.8)		0.87 (0.34, 2.24)	19 (53.3)	111 (39.8)	0.426	0.60 (0.17, 2.10)
Travel to usual facility Mean hours (SE)	1.97 (0.16)	1.84 (0.14)	0.832	1.03 (0.81, 1.29)	2.23 (0.25)	1.59 (0.07)	0.001	0.63 (0.48, 0.83)
Mother’s age Mean years (SE)	24.96 (0.82)	24.72 (0.70)	0.879	1.00 (0.95, 1.06)	25.89 (1.16)	26.67 (0.46)	0.432	1.02 (0.97, 1.07)
Mother’s education Mean years (SE)	3.84 (0.32)	4.60 (0.33)	0.085	1.10 (0.99, 1.21)	2.74 (0.45)	4.14 (0.23)	0.011	1.17 (1.04, 1.33)
**Niger**	238 (62.0)	145 (38.0)			69 (28.2)	176 (71.8)		
Neonatal cause of death								
All other causes	51 (21.3)	37 (25.5)	--	1.0 (ref)	--	--	--	--
IPRE or prematurity	76 (31.9)	23 (15.7)	0.051	0.43 (0.18, 1.01)	--	--	--	--
Severe infection	111 (46.7)	85 (58.7)	0.542	0.83 (0.46, 1.51)	--	--	--	--
Infant cause of death								
All other causes	--	--	--	--	19 (28.1)	55 (31.2)	--	1.0 (ref)
Severe febrile infection	--	--	--	--	50 (71.9)	121 (68.8)	0.708	0.89 (0.48, 1.65)
Neonatal age at death Mean days (SE)	5.80 (0.51)	9.10 (0.83)	0.002	1.06 (1.02, 1.10)	--	--	--	--
Infant age at death Mean months (SE)	--	--	--	--	4.63 (0.47)	5.75 (0.29)	0.054	1.12 (0.998, 1.26)
Illness severity^±^								
Mild	38 (15.9)	19 (13.0)	--	1.0 (ref)	10 (13.9)	11 (6.5)	--	1.0 (ref)
Moderate	61 (25.8)	51 (35.3)	0.216	1.60 (0.76, 3.38)	25 (36.5)	86 (48.7)	0.136	2.52 (0.75, 8.51)
Severe	139 (58.4)	75 (51.7)	0.304	1.50 (0.69, 3.26)	34 (49.7)	79 (44.8)	0.196	1.99 (0.70, 5.66)
Travel to usual facility Mean hours (SE)	1.43 (0.12)	1.04 (0.17)	0.270	0.83 (0.60, 1.15)	1.57 (0.19)	0.84 (0.08)	<0.001	0.60 (0.45, 0.81)
Mother’s age Mean years (SE)	26.32 (0.51)	25.62 (0.59)	0.246	0.98 (0.95, 1.01)	26.82 (0.79)	27.36 (0.80)	0.962	1.00 (0.96, 1.04)
Mother’s education Mean years (SE)	0.46 (0.11)	0.71 (0.19)	0.199	1.09 (0.96, 1.24)	0.31 (0.15)	0.95 (0.35)	0.0498	1.18 (1.00, 1.39)
**Tanzania**	64 (57.4)	48 (42.6)			23 (14.6)	133 (85.4)		
Neonatal cause of death								
All other causes	22 (33.9)	10 (21.9)	--	1.0 (ref)	--	--	--	--
IPRE or prematurity	23 (36.2)	11 (22.7)	0.627	1.51 (0.28, 8.33)	--	--	--	--
Severe infection	19 (29.9)	27 (55.4)	0.086	3.39 (0.84, 13.77)	--	--	--	--
Infant cause of death								
All other causes	--	--	--	--	10 (45.7)	43 (32.6)	--	1.0 (ref)
Severe febrile infection	--	--	--	--	12 (54.3)	89 (67.4)	0.294	1.80 (0.60, 5.45)
Neonatal age at death Mean days (SE)	2.89 (0.67)	5.54 (0.99)	0.043	1.09 (1.003, 1.19)	--	--	--	--
Infant age at death Mean months (SE)	--	--	--	--	3.84 (0.70)	5.99 (0.34)	0.046	1.26 (1.01, 1.58)
Illness severity^±^								
Mild	15 (23.2)	11 (24.0)	--	1.0 (ref)	3 (11.4)	27 (20.5)	--	1.0 (ref)
Moderate	7 (10.5)	15 (30.6)	0.042	4.83 (1.06, 21.96)	5 (23.0)	47 (35.1)	0.561	0.66 (0.16, 2.76)
Severe	43 (66.4)	22 (45.4)	0.595	1.45 (0.36, 5.87)	15 (65.6)	59 (44.4)	0.186	0.40 (0.10, 1.58)
Travel to usual facility Mean hours (SE)	0.84 (0.14)	1.57 (0.79)	0.077	1.15 (0.98, 1.35)	0.75 (0.15)	0.78 (0.07)	0.895	0.95 (0.42, 2.13)
Mother’s age Mean years (SE)	27.12 (2.53)	25.28 (1.14)	0.632	0.99 (0.93, 1.05)	25.92 (2.06)	27.72 (0.86)	0.563	1.03 (0.94, 1.12)
Mother’s education Mean years (SE)	5.74 (0.62)	6.39 (0.43)	0.101	1.14 (0.97, 1.33)	4.69 (0.73)	5.82 (0.28)	0.284	1.07 (0.95, 1.20)
**Mozambique**	217 (82.0)	48 (18.0)						
Neonatal cause of death								
All other causes	64 (29.6)	9 (18.5)	--	1.0 (ref)				
IPRE or prematurity	73 (33.8)	14 (29.6)	0.253	1.86 (0.64, 5.37)				
Severe infection	80 (36.6)	25 (51.9)	0.057	2.59 (0.97, 6.89)				
Neonatal age at death Mean days (SE)	5.24 (0.52)	10.55 (1.08)	<0.001	1.10 (1.04, 1.16)				
Illness severity^±^								
Mild	85 (39.0)	20 (41.5)	--	1.0 (ref)				
Moderate	46 (21.0)	20 (41.8)	0.022	3.05 (1.18, 7.92)				
Severe	87 (40.1)	8 (16.7)	0.290	0.57 (0.20, 1.62)				
Travel to usual facility Mean hours (SE)	9.00 (2.57)	3.41 (0.88)	0.273	0.97 (0.92, 1.03)				
Mother’s age Mean years (SE)	25.11 (0.82)	25.57 (1.59)	0.416	1.02 (0.97, 1.09)				
Mother’s education Mean years (SE)	3.79 (0.20)	5.60 (0.39)	<0.001	1.59 (1.32, 1.90)				
**Pakistan**	625 (50.9)	602 (49.1)						
Neonatal cause of death								
All other causes	233 (37.3)	195 (32.4)	--	1.0 (ref)				
IPRE or prematurity	176 (28.2)	150 (24.9)	0.841	0.97 (0.71, 1.32)				
Severe infection	216 (34.6)	257 (42.7)	0.084	1.28 (0.97, 1.69)				
Neonatal age at death Mean days (SE)	4.31 (0.24)	6.46 (0.28)	<0.001	1.07 (1.04, 1.09)				
Illness severity^±^								
Mild	162 (25.9)	105 (17.4)	--	1.0 (ref)				
Moderate	151 (24.2)	147 (24.4)	0.006	1.65 (1.16, 2.36)				
Severe	312 (49.9)	350 (58.1)	<0.001	2.22 (1.62, 3.03)				
Travel to usual facility Mean hours (SE)	1.40 (0.12)	0.97 (0.11)	0.085	0.96 (0.91, 1.01)				
Mother’s age Mean years (SE)	26.81 (0.30)	26.53 (0.23)	0.241	0.99 (0.97, 1.01)				
Mother’s education Mean years (SE)	7.03 (0.07)	7.95 (0.09)	<0.001	1.29 (1.18, 1.40)				

^Ω^Complete case analyses for Cameroon (missing 4.3% neonatal and 6.5% 1–11-months-old data points), Nigeria (missing 3.8% and 4.8%), Malawi (missing 2.9% and 6.0%), Niger (missing 7.7% and 8.9%), and Tanzania (missing 0% and 1.3%); Analyses with imputed means for travel time, mother’s age and/or mother’s schooling for Mozambique and Pakistan; Total Ns for neonates also exclude cases missing data for illness severity or formal careseeking (see Table C in S1 Appendix); ^£^Did not seek formal care at any time during the illness, and Sought formal care during the illness; ^±^Illness severity at onset (neonates) or on illness day-1 (1–11-month-olds); *Anova F-value for continuous variables, X2 for categorical variables; **Standard Error; ^β^Reference for each other level; ^€^IPRE: Intrapartum-related event (birth asphyxia or injury)

Moderate illness severity was associated with increased careseeking for neonates in Nigeria, Tanzania, Mozambique and Pakistan; but only in Pakistan was severe illness an apparent driver of careseeking. Older age was positively associated with careseeking in all seven countries, ranging from an increase of 6% in Niger to 11% in Cameroon for each additional day. For 1–11-month-olds, moderate illness severity doubled formal careseeking in Nigeria; while older age increased careseeking in three countries, by from 10% in Nigeria to 26% in Tanzania for each additional month, and nearly significantly so in Niger, by 12% (Fig 2b).

Cause of death played a significant role only in Nigeria and in Malawi, where neonates and 1–11-month-olds with an infectious cause were more than twice as likely to seek formal care. There was also a universal lack of increased careseeking for neonates with IPRE or prematurity, and nearly significant decrease in Niger. Mothers completing more years of school increased careseeking for neonates in four countries and for infants in three countries, by 8% to 59% for each additional year. Each additional hour of travel time to the usual facility used in an emergency decreased formal careseeking by about one-third for infants in two countries. Mother’s age significantly increased careseeking just for 1–11-month-olds in Nigeria, by 3% for each added year.

Going by the Table G in S1 Appendix findings would exclude Pakistan from the countries where careseeking was increased for older neonatal age, thus decreasing this effect from all seven countries to six, while adding Niger as a fourth country where older infant age boosted careseeking. This would also add Pakistan as a third country where careseeking was increased for infants’ severe infections and move Niger from a near decrease to a decrease in careseeking for IPRE or prematurity. Finally, using Table G’s (in S1 Appendix) findings would delete the positive effect of older maternal age on careseeking for infants in Nigeria.

## Discussion

We developed a simple 2-sign score capable of identifying mild, moderate, or severe neonatal illness based on feeding behavior and activity level, signs that have previously been shown to be both objective indicators of neonatal illness severity [7–9] and easily recognized motivators of careseeking for sick young children [5,6]. Caregivers have also been found to recognize inability to play, perhaps correlating with our ‘less active’ or ‘not moving’ signs, as indicators of severe fever in children under 5 years in Malawi [27]. Similarly, studies in India and Ghana have found lethargy to be one of the few signs of neonatal illness both easily recognized and understood by mothers to indicate the need for formal health care [28,29]. While the extreme levels of these signs are included among the IMCI illness signs requiring urgent referral, and poor feeding is an IMCI other illness sign, we found that the 2-sign scoring system was better able to differentiate mothers’ perception of mild, moderate, and severe neonatal illness at onset or on illness day-1 than were any one or more of 33 IMCI-VASA signs of moderate and severe illness. Our sensitivity test of the 2-sign method reinforced the validity of its assessments of illness severity of neonates by finding, as hypothesized, that a higher percentage of neonates who died on day-0 or day-1 of life were scored as being severely ill at onset. In contrast, one or more of 29 IMCI-VASA signs better distinguished mild, moderate, and severe illness of 1–11-month-olds than did the 2-sign method. This is perhaps to be expected given that illness in neonates is often characterized by less specific signs than in sick older children.

Verbal autopsy studies in general, as well as other studies, have assessed illness severity using individual signs for particular illnesses, for example, more than six loose stools in one day for severe diarrhea [24]. In addition to operating on only one illness at a time, this method also does not identify moderate illnesses more likely to be amenable to effective treatment. In contrast, our 2-sign and IMCI-VASA signs methods provide global measures of moderate and severe illness for all major causes of neonatal and infant mortality in low- and middle-income countries [30].

The 2-sign method identified a range of mild, moderate, and severe neonatal illnesses at onset or on illness day-1 in all seven study countries, demonstrating that caregivers were able to report various levels of these illness signs. This also enabled determining the association of these three levels of illness severity with formal careseeking. Only in Pakistan was there increased careseeking for both moderate and severe illness. The univariate negative association between careseeking and illness severity in two African countries and apparent negative association in three others was revealed by the multivariable analysis to in fact be a positive association between careseeking and moderate illness in three countries and no association between careseeking and the severity level in two countries.

Some authors have found that careseeking for young children is initiated only once illness reaches such a severe state that there is little chance of health care saving the child [12], and that this is due in part to lack of recognition or failing to act on signs of moderate illness [6]. Others have found decreased careseeking for severely ill neonates due in part to caregivers’ concern for their newborn’s fragility and the danger of taking such a young, sick child outside the home [31]. This explanation is also implied by our finding that, while neonatal age was uniformly inversely related to illness severity, in multivariable analyses careseeking was increased for older neonates in all seven countries but was increased for severe illness only in Pakistan. This suggests that caregivers in Pakistan were responding to their neonates’ severe illness, despite, or perhaps even more so because of, their young age; and warrants further study of the cultural or other factors driving this difference in careseeking behavior.

Increased careseeking for moderately ill neonates in Nigeria, Tanzania, and Mozambique, as well as in Pakistan, was perhaps encouraged by the relatively advanced age of the moderately ill as compared to the severely ill. A related possible explanation warranting further exploration is the perceived greater chance of survival of moderately ill neonates and a sense of fatalism overcoming the drive to seek care for younger, severely ill children. Mothers in a study of fatal childhood illnesses in Indonesia expressed this as “God’s will that the child would die” [32]. A counter argument put forth in response to this finding, that fatalism represents a post-hoc response to personally irremediable social constraints [33], leaves this explanation as wanting further analysis. Other possibilities include concern for mothers’ condition and cultural prohibitions against traveling soon after giving birth, and longer duration of moderately severe illness providing more time to seek care [32].

Contrary to the finding for neonates, there was no clear association between age and illness severity for 1–11-month-olds, though it appears that moderately ill infants were somewhat older than the mildly and severely ill. Yet, just as for neonates, in multivariable analyses age played the predominant role among 1–11-month-olds, with careseeking increased for older infants in three and nearly so in a fourth of five countries; while it was increased for moderate severity only in Nigeria and neither increased nor decreased for severe illness in all countries. These findings suggest that for infants beyond the neonatal period, another possible factor surpassing the degree of illness severity in the careseeking decision making process is the perceived greater chance of survival for an older child who has already traversed their most vulnerable period.

Our findings for both neonates and infants run counter to those of many studies in low resource settings that have found careseeking was increased for younger children, such as an examination of 258 national surveys datasets [34]. However, all these comparisons were of children 0–23 months versus 24–59 months old with a non-fatal illness and with minimal ability to examine the effect of illness severity. Other studies of careseeking for young children with a non-fatal illness have also found decreased careseeking for older children [35,36]; while a study of fatal illnesses of young children found, like our analysis, that careseeking increased in tandem with age increases [32]. A study of fatal childhood pneumonia deaths in Malawi also found that care was first sought at a health facility more often for children aged 12–59-months than for 1–11-month-olds and older children were more likely to die at a health center or hospital vs infants, who died more often at home [37]. In Taffa’s study of non-fatal illnesses [36] 95 percent of caretakers perceived their child’s morbidity as of mild to moderate severity, with no distinction between those who sought and did not seek care. The limited assessment of severity by the studies of children’s non-fatal illnesses, including secondary analyses of DHS data [38], makes it difficult to compare with studies of fatal illnesses. Our almost uniform findings across seven countries of increased careseeking for older neonates and infants, independent of illness severity, is strong evidence that should be examined for consistency in studies of fatal childhood illness in additional countries.

Regarding the impact of cause of death, similar to our finding of increased careseeking for infectious causes in Nigeria and Malawi, a study of 300 sick children in a slum in Bangladesh found the same in comparison to careseeking for malnourished children [39]. The universal lack of increased careseeking that we found for neonates with IPRE or prematurity, and nearly significant decrease in Niger, could be due to the often-rapid progression of these conditions to a severe state and very young age at which they result in death [40]. Very low levels of careseeking for preterm newborns have been documented in other settings [5,41].

The positive contribution of caregivers’ education to formal careseeking, as we found for both age groups in more than half the countries, was also found by two multi-country analyses of acute respiratory infection (ARI) [38] and diarrhea [42], although, again, these were both studies of non-fatal illnesses. Other multi-country studies of childhood diarrhea and ARI [24,43] have determined that health education about illness symptoms, rather than caregivers’ general education level, increased appropriate careseeking. While our study was not designed to examine place of death, the apparent association of no maternal education with home death of neonates and 1–11-month-olds seen in Tables 1a and 1b supports our finding of the positive association of maternal education with careseeking. Increased distance [35] and time to reach a health facility [39,44] have previously been found to negatively affect careseeking for young children. Positing that longer expected travel time might discourage careseeking, we included time to reach the usual provider in an emergency in our multivariable models and found this had a negative effect on careseeking only for infants and in just two countries. We could not include delay-1 (time to decide to seek formal health care, having recognized an illness) or delay-2 (time to prepare to travel and to reach formal care, having decided to seek care) in the model because these delays could be assessed only for children for whom care was sought.

### Limitations

Like all VASA studies of child mortality, the data collected and analyzed by this study were derived from interviews of deceased children’s caregivers. As such, we could not examine the performance of the severity scoring methods among children who survived an illness. Ideally, this would be done for children with a near-miss illness whose final severity level approximated that of the decedents. Being interview-based, with some interviews conducted beyond four years in five of the study countries, the findings are also subject to the possible influence of faulty recall and biased reporting of events. The severity scores developed by the study were based on caregivers’ perception of their children’s illness, without the ability to examine the children and objectively evaluate their condition. However, this concern is mitigated by the fact that the signs included in the 2-sign method have been shown to be recognized by and of concern to mothers and to correspond with actual illness severity. The consistent inverse relationship between perceived severity level and neonates’ age found by the study also suggests that the method accurately assesses illness severity, although theoretically one could argue that mothers might automatically perceive younger neonates as being more severely ill. The validity of the multiple sign method for 1–11-month-olds also depends on the accuracy of caregivers’ reports, so is similarly subject to possible inaccurate perception as well as interview and recall bias. However, the method is based on established indicators of illness severity and contributed to sensible findings, suggesting that it functioned well despite its possible limitations. From a statistical standpoint, power may be limited for some countries when examining the associations of illness severity and other factors with seeking formal health care. Finally, as described in the paper’s Methods section, it was not possible to do a comparative analysis of the 2-sign and IMCI-VASA methods’ performance in two of the seven study countries.

## Conclusions

The 2-sign and multiple-sign methods developed by this study for assessing caregivers’ perception, respectively, of their neonate’s and 1–11-month-old’s condition clearly differentiated mild, moderate, and severe illness at onset of a fatal illness. Due to the more specific signs associated with illnesses of older infants, the 2-sign method should be used only for neonates. Including the findings from these methods in multivariable analyses of careseeking showed that caregivers were more likely to seek care for moderately ill, older neonates and infants. Guidance on the illness signs for which care should be sought before a young child’s illness progresses to a severe state should be included in child survival messaging and is likely to contribute to decreases in child mortality. The 2-sign method can serve as a practical tool for this purpose for illnesses of neonates, while both the 2-sign and multiple sign methods can be included in mortality and morbidity studies to monitor caregivers’ response to their children’s illness condition and the impact of careseeking messages on child mortality. Qualitative studies are needed to better understand the reasons for reduced health careseeking for younger neonates and infants with a potentially fatal illness. However, it stands to reason that appropriate delivery and newborn care must be provided at community level to prevent and treat intrapartum-related events such as perinatal asphyxia and other early onset severe neonatal illnesses that progress rapidly and for which caregivers are unable to undertake effective careseeking.

## Supporting information

S1 Appendix **Table A in S1 Appendix**. Illness that started in the community. Table B in S1 Appendix. Kappa agreement with VASA 2-sign severity at illness onset. Table C in S1 Appendix. Among neonates whose illness started in the community, missing the following variables: Table D in S1 Appendix. Among neonates whose illness started in the community and not missing illness severity or sought formal care, but missing the following variables: Table E in S1 Appendix. Among 1–11-month-olds whose illness started in the community, missing the following variables: Table F in S1 Appendix. Among 1–11-month-olds whose illness started in the community and not missing illness severity or sought formal care, but missing the following variables: Table G in S1 Appendix. Logistic regression of factors associated with seeking formal health care for neonates and 1–11-month-olds with a fatal illness^Ω^. Table G in S1 Appendix legend. ^Ω^Analyses with imputed means for travel time, mother’s age and/or mother’s schooling for Cameroon, Nigeria, Malawi, Niger, and Tanzania. Complete case analyses for Mozambique (missing 34.7% cases) and Pakistan (missing 67.9%); Total Ns for neonates also exclude cases missing data for illness severity or formal careseeking (see Table C in S1 Appendix); *Standard Error; ^β^Reference for each other level; ^€^IPRE: Intrapartum-related event (birth asphyxia or birth injury).
(DOCX)

S1 Data Nigeria minimal neonatal dataset. (XLSX)

S2 Data Nigeria minimal 1–11-month-olds dataset. (XLSX)

S3 Data Malawi minimal neonatal dataset. (XLSX)

S4 Data Malawi minimal 1–11-month-olds dataset. (XLSX)

S5 Data Niger minimal neonatal dataset. (XLSX)

S6 Data Niger minimal 1–11-month-olds dataset. (XLSX)

S7 Data Tanzania minimal neonatal dataset. (XLSX)

S8 Data Tanzania minimal 1–11-month-olds dataset. (XLSX)

S9 Data Mozambique minimal neonatal dataset. (XLSX)

S1 Metadata Metadata for S1 Data-S9 Data. (DOCX)

S1 Checklist Inclusivity in global research. (DOCX)
