# Correction: Development of an effective illness severity measure and assessment of the impact of perceived illness severity on formal careseeking for fatal illnesses of neonates and infants in six sub-Saharan Africa countries and Pakistan

**DOI:** 10.1371/journal.pgph.0007239

**Published:** 2026-08-31

**Authors:** Henry D. Kalter, Jamie Perin, Zulfiqar A. Bhutta, Inuwa B. Jalingo, Paul R. Libite, Abdou Maina, Tiope Mleme, Mlemba A. Kamwe, Ivalda Macicame, Robert E. Black

There are a number of errors in Table 1. Please see the correct Table 1 here.

There are a number of errors in Panel 1, “2-sign method of classifying illness severity” Please see the complete, correct panel here.

**Table 1a:** Selected demographic characteristics of neonates in seven study countries. Key: *Household, **Wood/cement/tiles.

Characteristic	Cameroon N (%)	Nigeria N (%)	Malawi N (%)	Niger N (%)	Tanzania N (%)	Mozambique N (%)	Pakistan N (%)
**Child’s age (days)** **0-6** **7-27** **μ (SE) days**	116 (70.7) 48 (29.3) 4.6 (0.7)	535 (74.0) 188 (26.0) 4.5 (0.2)	224 (70.0) 96 (30.0) 5.1 (0.4)	311 (68.7) 142 (31.3) 6.1 (0.3)	195 (85.5) 33 (14.5) 3.1 (0.4)	302 (74.9) 101 (25.1) 4.5 (0.3)	1,629 (78.0) 459 (22.0) 4.3 (0.1)
**Sex** **Male** **Female** **Don’t know**	96 (58.5) 68 (41.5) 0 (0)	421 (58.2) 301 (41.6) 1 (0.1)	183 (57.2) 137 (42.8) 0 (0)	264 (58.3) 189 (41.7) 0 (0)	136 (59.6) 92 (40.4) 0 (0)	210 (54.8) 173 (45.2) 20 (5.0)	1282 (61.4) 806 (38.6) 0 (0)
**Birthplace** **Home** **Hospital** **Other facility** **Other** **Don’t know**	92 (56.1) 52 (31.7) 12 (7.3) 8 (4.9) 0 (0)	418 (57.8) 172 (23.8) 78 (10.8) 55 (7.6) 0 (0)	81 (25.3) 119 (37.2) 96 (30.0) 24 (7.5) 0 (0)	313 (69.1) 26 (5.7) 102 (22.5) 12 (2.6) 0 (0)	53 (23.2) 109 (47.8) 51 (22.4) 15 (6.6) 0 (0)	163 (40.4) 107 (26.6) 103 (25.6) 30 (7.4) 0 (0)	619 (29.6) 1,165 (55.8) 283 (13.6) 18 (0.9) 3 (0.1)
**Place of death** **Home** **Hospital** **Other facility** **Other** **Don’t know**	93 (56.7) 50 (30.5) 5 (3.0) 16 (9.8) 0 (0)	478 (66.1) 140 (19.4) 55 (7.6) 49 (6.8) 1 (0.1)	122 (38.1) 119 (37.2) 55 (17.2) 24 (7.5) 0 (0)	349 (77.0) 29 (6.4) 51 (11.3) 24 (5.3) 0 (0)	68 (29.8) 109 (47.8) 35 (15.4) 16 (7.0) 0 (0)	221 (54.8) 123 (30.5) 32 (7.9) 27 (6.7) 0 (0)	799 (38.3) 1,154 (55.3) 48 (2.3) 86 (4.1) 1 (0.05)
**Mother’s age (years)** **10-12** **13-19** **20-34** **35+** **Missing** **μ (SE) years**	6 (3.7) 61 (37.2) 75 (45.7) 17 (10.4) 5 (3.0) 22.7 (0.6)	0 (0) 150 (20.7) 464 (64.2) 106 (14.7) 3 (0.4) 26.5 (0.3)	0 (0) 83 (25.9) 196 (61.3) 34 (10.6) 7 (2.2) 24.5 (0.4)	0 (0) 96 (21.2) 275 (60.7) 63 (13.9) 19 (4.2) 25.5 (0.3)	0 (0) 52 (22.8) 133 (58.3) 41 (18.0) 2 (0.9) 26.2 (0.5)	0 (0) 127 (31.5) 228 (56.6) 45 (11.2) 3 (0.7) 24.7 (0.4)	0 (0) 276 (13.2) 1,537 (73.6) 246 (11.8) 29 (1.4) 26.5 (0.1)
**Mother’s education** **None** **Primary** **Secondary** **Higher** **Missing**	4 (2.4) 108 (65.9) 50 (30.5) 2 (1.2) 0 (0)	350 (48.4) 168 (23.2) 162 (22.4) 28 (3.9) 15 (2.1)	46 (14.4) 181 (56.6) 91 (28.4) 1 (0.3) 1 (0.3)	382 (84.3) 49 (10.8) 14 (3.1) 1 (0.2) 7 (1.5)	30 (13.2) 39 (17.1) 151 (66.2) 8 (3.5) 0 (0)	7 (1.7) 198 (49.1) 90 (22.3) 0 (0) 108 (26.8)	2 (0.1) 329 (15.8) 358 (17.1) 72 (3.4) 1,327 (63.6)
HH* electricity **Yes** **No**	42 (25.6) 122 (74.4)	315 (43.6) 408 (56.4)	11 (3.4) 309 (96.6)	39 (8.6) 414 (91.4)	49 (21.5) 179 (78.5)	82 (20.3) 321 (79.7)	1,983 (95.0) 105 (5.0)
HH* floor material **Mud/clay** W/C/T** **Other** **Don’t know**	132 (80.5) 32 (19.5) 0 (0) 0 (0)	246 (34.0) 442 (61.1) 35 (4.8) 0 (0)	290 (90.6) 29 (9.1) 1 (0.3) 0 (0)	416 (91.8) 36 (7.9) 1 (0.2) 0 (0)	134 (58.8) 94 (41.2) 0 (0) 0 (0)	320 (79.4) 83 (20.6) 0 (0) 0 (0)	898 (43.0) 1,159 (55.0) 30 (1.4) 1 (0.05)

**Table 1b:** Selected demographic characteristics of 1-11-month-olds in five study countries. Key: *Household, **Wood/cement/tiles.

Characteristic	Cameroon N (%)	Nigeria N (%)	Malawi N (%)	Niger N (%)	Tanzania N (%)
**Child’s age (months)** **1-2** **3-6** **7-11** **μ (SE) months**	33 (12.6) 96 (36.8) 132 (50.6) 6.5 (0.2)	159 (23.0) 228 (33.0) 304 (44.0) 5.8 (0.1)	69 (20.6) 115 (34.3) 151 (45.1) 5.8 (0.2)	60 (22.3) 91 (33.8) 118 (43.9) 5.6 (0.2)	41 (25.9) 50 (31.6) 67 (42.4) 5.5 (0.3)
**Sex** **Male** **Female**	136 (52.1) 125 (47.9)	350 (50.7) 341 (49.3)	166 (49.6) 169 (50.4)	137 (50.9) 132 (49.1)	81 (51.3) 77 (48.7)
**Birthplace** **Home** **Hospital** **Other facility** **Other**	180 (69.0) 45 (17.2) 33 (12.6) 3 (1.1)	431 (62.4) 156 (22.6) 79 (11.4) 25 (3.6)	54 (16.1) 153 (45.7) 106 (31.6) 22 (6.6)	195 (72.5) 7 (2.6) 62 (23.0) 5 (1.9)	50 (31.6) 58 (36.7) 44 (27.8) 6 (3.8)
**Place of death** **Home** **Hospital** **Other facility** **Other** **Don’t know**	146 (55.9) 51 (19.5) 19 (7.3) 45 (17.2) 0 (0)	484 (70.0) 127 (18.4) 30 (4.3) 49 (7.1) 1 (0.1)	155 (46.3) 119 (35.5) 28 (8.4) 33 (9.9) 0 (0)	215 (79.9) 18 (6.7) 20 (7.4) 15 (5.6) 1 (0.4)	65 (41.1) 53 (33.5) 13 (8.2) 27 (17.1) 0 (0)
**Mother’s age (years)** **10-12** **13-19** **20-34** **35+** **Missing** **μ (SE) years**	2 (0.8) 85 (32.6) 138 (52.9) 29 (11.2) 7 (2.7) 23.9 (0.5)	1 (0.1) 103 (14.9) 463 (67.0) 120 (17.4) 4 (0.6) 27.1 (0.3)	0 (0) 54 (16.1) 217 (64.8) 48 (14.3) 16 (4.8) 26.8 (0.4)	0 (0) 37 (13.8) 174 (64.7) 37 (13.8) 21 (7.8) 26.9 (0.5)	0 (0) 28 (17.7) 90 (57.0) 37 (23.4) 3 (1.9) 27.6 (0.6)
**Mother’s education** **None** **Primary** **Secondary** **Higher** **Missing**	4 (1.5) 190 (72.8) 63 (24.1) 0 (0) 4 (1.5)	337 (48.8) 164 (23.7) 129 (18.7) 40 (5.8) 21 (3.0)	76 (22.7) 184 (54.9) 72 (21.5) 2 (0.6) 1 (0.3)	233 (86.6) 23 (8.6) 7 (2.6) 0 (0) 6 (2.2)	30 (19.0) 24 (15.2) 104 (65.8) 0 (0) 0 (0)
HH* electricity **Yes** **No**	69 (26.4) 192 (73.6)	305 (44.1) 386 (55.9)	3 (0.9) 332 (99.1)	24 (8.9) 245 (91.1)	26 (16.5) 132 (83.5)
HH* floor material **Mud/clay** W/C/T** **Other**	218 (83.5) 43 (16.5) 0 (0)	241 (34.9) 422 (61.1) 28 (4.1)	295 (88.1) 40 (11.9) 0 (0)	244 (90.7) 25 (9.3) 0 (0)	108 (68.4) 50 (31.6) 0 (0)

**Panel 1:** 2-sign method of classifying illness severity

	Levels and assigned scores		
Individual parameters	Normal = 1	Medium = 2	Abnormal = 3
Feeding	Normally	Poorly	Not at all
Activity	Normal	Less active	Not moving
Combined scores and final analysis ranks			
Illness level	Signs	Score	Rank
Mild	2 normal or 1 normal and 1 medium	2 3	1 1
Moderate	1 normal and 1 abnormal or 2 medium	4 4	2 2
Severe	1 medium and 1 abnormal or 2 abnormal	5 6	3 3
