# Supplementary material for: Development of an effective illness severity measure and assessment of the impact of perceived illness severity on formal careseeking for fatal illnesses of neonates and infants in six sub-Saharan Africa countries and Pakistan
Source: PLOS Glob Public Health. 2026 May 22;6(5):e0006455. doi: 10.1371/journal.pgph.0006455 (PMC13197068; doi:10.1371/journal.pgph.0006455)
Supplement: S1 Appendix — Table A in S1 Appendix. Illness that started in the community. Table B in S1 Appendix. Kappa agreement with VASA 2-sign severity at illness onset. Table C in S1 Appendix. Among neonates whose illness started in the community, missing the following variables: Table D in S1 Appendix. Among neonates whose illness started in the community and not missing illness severity or sought formal care, but missing the following variables: Table E in S1 Appendix. Among 1–11-month-olds whose illness started in the community, missing the following variables: Table F in S1 Appendix. Among 1–11-month-olds whose illness started in the community and not missing illness severity or sought formal care, but missing the following variables: Table G in S1 Appendix. Logistic regression of factors associated with seeking formal health care for neonates and 1–11-month-olds with a fatal illnessΩ. Table G in S1 Appendix legend. ΩAnalyses with imputed means for travel time, mother’s age and/or mother’s schooling for Cameroon, Nigeria, Malawi, Niger, and Tanzania. Complete case analyses for Mozambique (missing 34.7% cases) and Pakistan (missing 67.9%); Total Ns for neonates also exclude cases missing data for illness severity or formal careseeking (see Table C in S1 Appendix); *Standard Error; βReference for each other level; €IPRE: Intrapartum-related event (birth asphyxia or birth injury). (DOCX) [file pgph.0006455.s001.docx]

**S1 Appendix**

**Table A. Illness that started in the community**

**Country Neonates 1-11-month-olds**

Cameroon: 142/164 (86.6%) 261 (100%)

Nigeria: 557/723 (77.0%) 691 (100%)

Malawi 205/320 (64.1%) 335 (100%)

Niger: 438/453 (96.7%) 269 (100%)

Tanzania: 115/228 (50.4%) 158 (100%)

Mozambique: 289/402 (71.9%) --

Pakistan: 1381/2088 (66.1%) --

| **Table B. Kappa agreement with VASA 2-sign severity at illness onset** | | |
| --- | --- | --- |
| **Country** | **IMC day-1 illness severity** | **IMCI-VASA day-1 illness signs** |
| **Neonates** |  |  |
| Cameroon | 0.22 | 0.01 |
| Nigeria | 0.31 | 0.25 |
| Malawi | 0.17 | 0.06 |
| Niger | 0.24 | 0.07 |
| Tanzania | 0.42 | 0.16 |
| **1-11 month olds** |  |  |
| Cameroon | 0.70 | 0.43 |
| Nigeria | 0.78 | 0.61 |
| Malawi | 0.69 | 0.48 |
| Niger | 0.71 | 0.47 |
| Tanzania | 0.29 | 0.37 |

**Table C. Among neonates whose illness started in the community, missing the following variables:**

**Country Illness severity Sought formal care Cause of death Age (days) Travel time Mother’s age Mother’s schooling**

Cameroon 3/142 (2.1%) 0/142 (0%) 0/142 (0%) 0/142 (0%) 2/142 (1.4%) 5/142 (3.5%) 0/142 (0%)

Nigeria 11/557 (1.9%) 0/557 (0%) 0/557 (0%) 0/557 (0%) 7/557 (1.2%) 3/557 (0.5%) 14/557 (2.5%)

Malawi 1/205 (0.5%) 0/205 (0%) 0/205 (0%) 0/205 (0%) 2/205 (0.8%) 5/205 (2.2%) 1/205 (0.3%)

Niger 18/438 (4.2%) 5/438 (1.0%) 0/438 (0%) 0/438 (0%) 20/438 (4.5%) 12/438 (2.8%) 7/438 (1.6%)

Tanzania 2/115 (2.0%) 0/115 (0%) 0/115 (0%) 0/115 (0%) 0/115 (0%) 0/115 (0%) 0/115 (0%)

Mozambique 22/289 (7.7%) 3/289 (1.1%) 0/289 (0%) 0/289 (0%) 9/289 (3.2%) 3/289 (1.2%) 92/289 (31.7%)

Pakistan 154/1381 (11.2%) 0/1381 (0%) 0/1381 (0%) 0/1381 (0%) 42/1381 (3.0%) 20/1381 (1.4%) 937/1381 (67.8%)

**Table D. Among neonates whose illness started in the community and not missing illness severity or sought formal care, but missing the following variables:**

**Country Cause of death Age (days) Travel time Mother’s age Mother’s schooling**

Cameroon 0/139 (0%) 0/139 (0%) 2/139 (1.4%) 5/139 (3.6%) 0/139 (0%)

Nigeria 0/546 (0%) 0/546 (0%) 7/546 (1.2%) 3/546 (0.5%) 14/546 (2.5%)

Malawi 0/204 (0%) 0/204 (0%) 2/204 (0.8%) 5/204 (2.2%) 1/204 (0.3%)

Niger 0/416 (0%) 0/416 (0%) 18/416 (4.4%) 12/416 (2.8%) 7/416 (1.7%)

Tanzania 0/112 (0%) 0/112 (0%) 0/112 (0%) 0/112 (0%) 0/112 (0%)

Mozambique 0/265 (0%) 0/265 (0%) 9/265 (3.5%) 3/265 (1.3%) 88/265 (33.1%)

Pakistan 0/1227 (0%) 0/1227 (0%) 32/1227 (2.6%) 11/1227 (0.9%) 827/1227 (67.4%)

**Table E. Among 1-11-month-olds whose illness started in the community, missing the following variables:**

**Country Illness severity Sought formal care Cause of death Age (months) Travel time Mother’s age Mother’s schooling**

Cameroon 0/261 (0%) 0/261 (0%) 0/261 (0%) 0/261 (0%) 6/261 (2.3%) 7/261 (2.7%) 4/261 (1.5%)

Nigeria 0/691 (0%) 0/691 (0%) 0/691 (0%) 0/691 (0%) 11/691 (1.6%) 4/691 (0.6%) 18/691 (2.6%)

Malawi 0/335 (0%) 0/335 (0%) 0/335 (0%) 0/335 (0%) 3/335 (0.8%) 17/335 (5.0%) 1/335 (0.3%)

Niger 0/269 (4.2%) 0/269 (0%) 0/269 (0%) 0/269 (0%) 6/269 (2.2%) 14/269 (5.1%) 6/269 (2.3%)

Tanzania 0/158 (0%) 0/158 (0%) 0/158 (0%) 0/158 (0%) 1/158 (0.3%) 3/158 (1.8%) 0/158 (0%)

**Table F. Among 1-11-month-olds whose illness started in the community and not missing illness severity or sought formal care, but missing the following variables:**

**Country Cause of death Age (months) Travel time Mother’s age Mother’s schooling**

Cameroon 0/261 (0%) 0/261 (0%) 6/261 (2.3%) 7/261 (2.7%) 4/261 (1.5%)

Nigeria 0/691 (0%) 0/691 (0%) 11/691 (1.6%) 4/691 (0.6%) 18/691 (2.6%)

Malawi 0/335 (0%) 0/335 (0%) 3/335 (0.8%) 17/335 (5.0%) 1/335 (0.3%)

Niger 0/269 (0%) 0/269 (0%) 6/269 (2.2%) 14/269 (5.1%) 6/269 (2.3%)

Tanzania 0/158 (0%) 0/158 (0%) 0/158 (0%) 3/158 (1.8%) 0/158 (0%)

**Table G. Logistic regression of factors associated with seeking formal health care for neonates and 1-11-month-olds with a fatal illness**^Ω^

| **Country**  **Explanatory factors** | **Neonates (0-27 days)** | | | | **Infants (1-11 months old)** | | | |
| --- | --- | --- | --- | --- | --- | --- | --- | --- |
|  | **Did not seek formal care^£^**  **N (%)** | **Sought**  **formal care^£^**  **N (%)** | **p-value*** | **OR (95% CI)** | **Did not seek formal care^£^**  **N (%)** | **Sought**  **formal care^£^**  **N (%)** | **p-value*** | **OR (95% CI)** |
| **Cameroon** | 82 (59.0) | 57 (41.0) |  |  | 59 (22.6) | 202 (77.4) |  |  |
| Neonatal cause of death  All other causes  IPRE^€^ or prematurity  Severe infection | 11 (13.4)  48 (58.5)  23 (28.0) | 5 (8.8)  23 (40.4)  29 (50.9) | --  0.586  0.111 | 1.0 (ref^β^)  1.44 (0.39, 5.30)  2.89 (0.78, 10.67) | --  --  -- | --  --  -- | --  --  -- | --  --  -- |
| Infant cause of death  All other causes  Severe febrile infection | --  -- | --  -- | --  -- | --  -- | 22 (37.3)  37 (62.7) | 73 (36.1)  129 (63.9) | --  0.791 | 1.0 (ref)  1.09 (0.59, 2.01) |
| Neonatal age at death  Mean days (SE**) | 2.96 (0.45) | 6.86 (0.86) | 0.003 | 1.14 (1.05, 1.24) | -- | -- | -- | -- |
| Infant age at death  Mean months (SE) | -- | -- | -- | -- | 5.76 (0.41) | 6.74 (0.21) | 0.046 | 1.11 (1.002, 1.22) |
| Illness severity^±^  Mild  Moderate  Severe | 6 (7.3)  19 (23.2)  57 (69.5) | 12 (21.1)  17 (29.8)  28 (49.1) | --  0.249  0.189 | 1.0 (ref)  0.47 (0.13, 1.70)  0.44 (0.13, 1.50) | 12 (20.3)  17 (28.8)  30 (50.9) | 39 (19.3)  88 (43.6)  75 (37.1) | --  0.364  0.492 | 1.0 (ref)  1.47 (0.64, 3.40)  0.76 (0.34, 1.67) |
| Travel to usual facility  Mean hours (SE) | 0.70 (0.09) | 0.70 (0.11) | 0.815 | 1.06 (0.66, 1.70) | 0.77 (0.09) | 0.81 (0.06) | 0.447 | 1.16 (0.80, 1.68) |
| Mother’s age  Mean years (SE) | 22.24 (0.76) | 22.09 (1.07) | 0.938 | 1.00 (0.95, 1.05) | 24.69 (0.97) | 23.72 (0,50) | 0.507 | 0.99 (0.95, 1.03) |
| Mother’s education  Mean years (SE) | 5.68 (0.24) | 5.86 (0.28) | 0.039 | 1.23 (1.01, 1.50) | 5.19 (0.25) | 5.41 (0.14) | 0.718 | 1.03 (0.88, 1.20) |
| **Nigeria** | 400 (73.2) | 146 (26.8) |  |  | 272 (39.4) | 419 (60.6) |  |  |
| Neonatal cause of death  All other causes  IPRE or prematurity  Severe infection | 105 (26.2)  112 (28.0)  183 (45.8) | 32 (22.0)  18 (12.4)  96 (65.6) | --  0.638  0.087 | 1.0 (ref)  0.82 (0.35, 1.92)  1.64 (0.93, 2.88) | --  --  -- | --  --  -- | --  --  -- | --  --  -- |
| Infant cause of death  All other causes  Severe febrile infection | --  -- | --  -- | --  -- | --  -- | 94 (34.5)  178 (65.5) | 122 (29.1)  297 (70.9) | --  0.009 | 1.0 (ref)  1.67 (1.14, 2.45) |
| Neonatal age at death  Mean days (SE) | 4.47 (0.29) | 7.70 (0.51) | 0.004 | 1.06 (1.02, 1.10) | -- | -- | -- | -- |
| Infant age at death  Mean months (SE) | -- | -- | -- | -- | 5.38 (0.19) | 5.96 (0.17) | 0.011 | 1.08 (1.02, 1.15) |
| Illness severity^±^  Mild  Moderate  Severe | 41 (10.3)  175 (43.8)  184 (45.9) | 7 (5.0)  114 (77.8)  25 (17.2) | --  0.005  0.663 | 1.0 (ref)  3.79 (1.49, 9.64)  1.27 (0.43, 3.72) | 37 (13.6)  157 (57.7)  78 (28.6) | 31 (7.4)  290 (69.2)  98 (23.5) | --  0.013  0.142 | 1.0 (ref)  2.27 (1.19, 4.32)  1.66 (0.84, 3.26) |
| Travel to usual facility  Mean hours (SE) | 0.90 (0.05) | 0.83 (0.07) | 0.560 | 0.90 (0.63, 1.29) | 0.94 (0.05) | 0.90 (0.16) | 0.736 | 0.98 (0.86, 1.11) |
| Mother’s age  Mean years (SE) | 25.83 (0.37) | 26.91 (0.66) | 0.251 | 1.02 (0.99, 1.05) | 26.00 (0.51) | 27.65 (0.38) | 0.073 | 1.02 (0.998, 1.05) |
| Mother’s education  Mean years (SE) | 2.48 (0.21) | 4.11 (0.38) | 0.001 | 1.09 (1.03, 1.14) | 2.16 (0.21) | 5.01 (0.26) | <0.001 | 1.16 (1.11, 1.21) |
| **Malawi** | 97 (47.6) | 107 (52.4) |  |  | 41 (12.2) | 294 (87.8) |  |  |
| Neonatal cause of death  All other causes  IPRE or prematurity  Severe infection | 31 (32.3)  31 (31.7)  35 (36.0) | 20 (18.5)  24 (22.7)  63 (58.8) | --  0.112  0.024 | 1.0 (ref)  2.13 (0.84, 5.41)  2.50 (1.13, 5.54) | --  --  -- | --  --  -- | --  --  -- | --  --  -- |
| Infant cause of death  All other causes  Severe febrile infection | --  -- | --  -- | --  -- | --  -- | 22 (53.9)  19 (46.1) | 100 (34.0)  194 (66.0) | --  0.009 | 1.0 (ref)  2.65 (1.28, 5.48) |
| Neonatal age at death  Mean days (SE) | 4.46 (0.63) | 9.46 (0.88) | 0.001 | 1.10 (1.04, 1.16) | -- | -- | -- | -- |
| Infant age at death  Mean months (SE) | -- | -- | -- | -- | 4.98 (0.54) | 5.94 (0.19) | 0.123 | 1.10 (0.97, 1.25) |
| Illness severity^±^  Mild  Moderate  Severe | 16 (16.6)  33 (34.4)  48 (49.1) | 27 (25.0)  46 (43.0)  34 (31.9) | --  0.503  0.625 | 1.0 (ref)  1.36 (0.55, 3.39)  0.80 (0.32, 2.00) | 5 (13.1)  13 (30.8)  23 (56.1) | 58 (19.8)  122 (41.4)  114 (38.9) | --  0.935  0.166 | 1.0 (ref)  0.95 (0.30, 3.07)  0.44 (0.14, 1.40) |
| Travel to usual facility  Mean hours (SE) | 1.96 (0.15) | 1.84 (0.14) | 0.817 | 1.03 (0.82, 1.29) | 2.21 (0.22) | 1.60 (0.07) | 0.001 | 0.65 (0.50, 0.84) |
| Mother’s age  Mean years (SE) | 24.99 (0.78) | 24.78 (0.69) | 0.862 | 1.00 (0.96, 1.06) | 25.67 (1.02) | 26.66 (0.44) | 0.373 | 1.02 (0.97, 1.07) |
| Mother’s education  Mean years (SE) | 3.78 (0.31) | 4.59 (0.32) | 0.066 | 1.10 (0.99, 1.22) | 2.83 (0.43) | 4.10 (0.22) | 0.025 | 1.15 (1.02, 1.30) |
| **Niger** | 262 (62.9) | 154 (37.1) |  |  | 77 (28.5) | 192 (71.5) |  |  |
| Neonatal cause of death  All other causes  IPRE or prematurity  Severe infection | 56 (21.2)  84 (32.1)  122 (46.6) | 38 (25.0)  23 (14.8)  93 (60.2) | --  0.043  0.766 | 1.0 (ref)  0.42 (0.18, 0.97)  0.92 (0.52, 1.63) | --  --  -- | --  --  -- | --  --  -- | --  --  -- |
| Infant cause of death  All other causes  Severe febrile infection | --  -- | --  -- | --  -- | --  -- | 24 (31.2)  53 (68.8) | 59 (30.5)  134 (69.5) | --  0.620 | 1.0 (ref)  1.16 (0.64, 2.12) |
| Neonatal age at death  Mean days (SE) | 5.79 (0.48) | 8.85 (0.79) | 0.005 | 1.05 (1.02, 1.09) | -- | -- | -- | -- |
| Infant age at death  Mean months (SE) | -- | -- | -- | -- | 4.71 (0.43) | 5.82 (0.28) | 0.027 | 1.12 (1.01, 1.24) |
| Illness severity^±^  Mild  Moderate  Severe | 41 (15.6)  67 (25.7)  154 (58.7) | 20 (13.2)  56 (36.2)  78 (50.6) | --  0.206  0.392 | 1.0 (ref)  1.59 (0.77, 3.29)  1.39 (0.65, 2.97) | 10 (12.6)  27 (35.6)  40 (51.8) | 13 (7.0)  90 (46.7)  89 (46.3) | --  0.282  0.503 | 1.0 (ref)  1.93 (0.58, 6.41)  1.40 (0.52, 3.79) |
| Travel to usual facility  Mean hours (SE) | 1.44 (0.11) | 1.02 (0.16) | 0.252 | 0.82 (0.57, 1.16) | 1.51 (0.18) | 0.90 (0.09) | 0.018 | 0.70 (0.52, 0.94) |
| Mother’s age  Mean years (SE) | 26.14 (0.48) | 25.45 (0.54) | 0.252 | 0.98 (0.95, 1.01) | 26.81 (0.71) | 27.26 (0.74) | 0.867 | 1.00 (0.97, 1.04) |
| Mother’s education  Mean years (SE) | 0.47 (0.10) | 0.68 (0.18) | 0.311 | 1.07 (0.94, 1.22) | 0.28 (0.14) | 0.90 (0.33) | 0.029 | 1.21 (1.02, 1.43) |
| **Tanzania** | 64 (57.4) | 48 (42.6) |  |  | 25 (15.5) | 133 (84.5) |  |  |
| Neonatal cause of death  All other causes  IPRE or prematurity  Severe infection | 22 (33.9)  23 (36.2)  19 (29.9) | 10 (21.9)  11 (22.7)  27 (55.4) | --  0.627  0.086 | 1.0 (ref)  1.51 (0.28, 8.33)  3.39 (0.84, 13.77) | --  --  -- | --  --  -- | --  --  -- | --  --  -- |
| Infant cause of death  All other causes  Severe febrile infection | --  -- | --  -- | --  -- | --  -- | 10 (42.1)  14 (57.9) | 43 (32.4)  90 (67.6) | --  0.294 | 1.0 (ref)  1.80 (0.60, 5.45) |
| Neonatal age at death  Mean days (SE) | 2.89 (0.67) | 5.54 (0.99) | 0.043 | 1.09 (1.003, 1.19) | -- | -- | -- | -- |
| Infant age at death  Mean months (SE) | -- | -- | -- | -- | 3.79 (0.67) | 6.01 (0.34) | 0.046 | 1.26 (1.01, 1.58) |
| Illness severity^±^  Mild  Moderate  Severe | 15 (23.2)  7 (10.5)  43 (66.4) | 11 (24.0)  15 (30.6)  22 (45.4) | --  0.042  0.595 | 1.0 (ref)  4.83 (1.06, 21.96)  1.45 (0.36, 5.87) | 3 (10.5)  5 (21.1)  17 (68.4) | 27 (20.4)  47 (35.5)  59 (44.1) | --  0.561  0.186 | 1.0 (ref)  0.66 (0.16, 2.76)  0.40 (0.10, 1.58) |
| Travel to usual facility  Mean hours (SE) | 0.84 (0.14) | 1.57 (0.79) | 0.077 | 1.15 (0.98, 1.35) | 0.82 (0.15) | 0.78 (0.07) | 0.895 | 0.95 (0.42, 2.13) |
| Mother’s age  Mean years (SE) | 27.12 (2.53) | 25.28 (1.14) | 0.632 | 0.99 (0.93, 1.05) | 25.92 (2.06) | 27.72 (0.86) | 0.563 | 1.03 (0.94, 1.12) |
| Mother’s education  Mean years (SE) | 5.74 (0.62) | 6.39 (0.43) | 0.101 | 1.14 (0.97, 1.33) | 4.52 (0.72) | 5.78 (0.28) | 0.284 | 1.07 (0.95, 1.20) |
| **Mozambique** | 141 (81.3) | 32 (18.7) |  |  |  | | | |
| Neonatal cause of death  All other causes  IPRE or prematurity  Severe infection | 42 (29.6)  48 (34.1)  51 (36.4) | 6 (18.1)  9 (26.5)  18 (55.5) | --  0.419  0.107 | 1.0 (ref)  1.71 (0.46, 6.29)  2.82 (0.80, 9.95) |  |  |  |  |
| Neonatal age at death  Mean days (SE) | 5.23 (0.67) | 10.06 (1.43) | 0.009 | 1.10 (1.02, 1.18) |  |  |  |  |
| Illness severity^±^  Mild  Moderate  Severe | 57 (40.3)  28 (20.0)  56 (39.7) | 12 (36.2)  16 (48.6)  5 (15.2) | --  0.012  0.522 | 1.0 (ref)  5.51 (1.47, 20.67)  0.63 (0.15, 2.65) |  |  |  |  |
| Travel to usual facility  Mean hours (SE) | 8.15 (2.32) | 2.95 (0.92) | 0.140 | 0.92 (0.83, 1.03) |  |  |  |  |
| Mother’s age  Mean years (SE) | 23.72 (0.86) | 22.37 (0.98) | 0.549 | 0.97 (0.89, 1.06) |  |  |  |  |
| Mother’s education  Mean years (SE) | 3.79 (0.31) | 5.66 (0.58) | <0.001 | 1.43 (1.18, 1.74) |  |  |  |  |
| **Pakistan** | 158 (40.1) | 236 (59.9) |  |  |  | | | |
| Neonatal cause of death  All other causes  IPRE or prematurity  Severe infection | 65 (41.1)  46 (29.1)  47 (29.7) | 75 (31.8)  68 (28.8)  93 (39.4) | --  0.532  0.042 | 1.0 (ref^β^)  1.17 (0.72, 1.90)  1.68 (1.02, 2.75) |  |  |  |  |
| Neonatal age at death  Mean days (SE) | 5.90 (0.56) | 6.25 (0.40) | 0.438 | 1.01 (0.98, 1.05) |  |  |  |  |
| Illness severity^±^  Mild  Moderate  Severe | 56 (35.4)  39 (24.7)  63 (39.9) | 46 (19.5)  63 (26.7)  127 (53.8) | --  0.010  <0.001 | 1.0 (ref^β^)  2.01 (1.18, 3.40)  2.75 (1.67, 4.51) |  |  |  |  |
| Travel to usual facility  Mean hours (SE) | 1.57 (0.44) | 0.62 (0.09) | 0.075 | 0.87 (0.75, 1.01) |  |  |  |  |
| Mother’s age  Mean years (SE) | 25.42 (0.41) | 25.61 (0.33) | 0.793 | 1.01 (0.96, 1.05) |  |  |  |  |
| Mother’s education  Mean years (SE) | 7.03 (0.24) | 8.00 (0.23) | 0.008 | 1.09 (1.02, 1.15) |  |  |  |  |

^Ω^Analyses with imputed means for travel time, mother’s age and/or mother’s schooling for Cameroon, Nigeria, Malawi, Niger, and Tanzania. Complete case analyses for Mozambique (missing 34.7% cases) and Pakistan (missing 67.9%); Total Ns for neonates also exclude cases missing data for illness severity or formal careseeking (see Table C in S1 Appendix); **^£^**Did not seek formal care at any time during the illness, and Sought formal care during the illness; ^±^Illness severity at onset (neonates) or on illness day-1 (1-11-month-olds); *Anova F-value for continuous variables, X^2^ for categorical variables; **Standard Error; ^β^Reference for each other level; ^€^IPRE: Intrapartum-related event (birth asphyxia or birth injury)
