# Supplementary material for: Development of an effective illness severity measure and assessment of the impact of perceived illness severity on formal careseeking for fatal illnesses of neonates and infants in six sub-Saharan Africa countries and Pakistan
Source: PLOS Glob Public Health. 2026 May 22;6(5):e0006455. doi: 10.1371/journal.pgph.0006455 (PMC13197068; doi:10.1371/journal.pgph.0006455)
Supplement: S1 Metadata — (DOCX) [file pgph.0006455.s011.docx]

**Neonatal deaths_metadata**

**All countries variable codes**

Country: 1=Cameroon, 2=Nigeria, 3=Malawi, 4=Niger, 5=Tanzania, 6=Mozambique, 7=Pakistan

Urbrur: 1=urban, 2=rural

Yes/No variables: 1=yes, 2=no, 9=DK, 8=refused

Variables not listed below are described by their data label and their levels are self-evident

**Mozambique and Pakistan variable codes**

Q1403 – respondent: 1=spouse, 2=mother, 3=father, 4=grandmother, 5=grandfather, 6=son, 7=daughter, 8=aunt, 9=uncle, 10=brother, 11=sister, 12=birth attendant, 13=other male, 14=other female

N2006 – birthplace: 1=mother’s home, 2=other home, 3=government hospital, 4=government clinic/health center, 5=government health post, 6=other public facility, 7=private hospital, 8=private clinic, 9=private maternity home, 10=other private medical facility, 11=on route to health provider/facility, 12=other, 99=DK, 88=refused

Q1604 – place of child’s death: 1=hospital, 2=other health provider/facility, 3=on route to a health provider/facility, 4=home, 5=other, 9=DK, 8=refused

Q1203 – child’s sex: 1=male, 2=female, 9=DK, 8=refused

Q1419a – floor material: 1=earth beaten (clay), 2=earth not beaten, 3=rudimentary wood, 4=adobe, 5=parquet/polished wood, 6=tiles, 7=cement, 8=other, 9=DK, 88=refused

N2122 – minutes after birth cried: (N)umber recorded, 98=never cried, 99=DK, 88=refused

N2186 – number of loose stools (on worst day): (N)umber recorded, 99=DK, 88=refused

Mothersage_at_childsdeath: (N)umber recorded, blank=missing

Motherschoolyears: (N)umber recorded, blank=missing

Hourstravel_to_usualfacility2: (N)umber recorded, blank=missing

N2211_1 – feeding at illness onset: 1=normally 2=poorly, 3=not at all, 9=DK

N2211_2 – activity at illness onset: 1=normal, 2=less active, 3=not moving, 9=DK

svrsyndrm_rank_illness_onset_2s: 1=no/mild illness, 2=moderate illness, 3=severe illness, blank=missing

formalcare – received any formal healthcare during illness: 1=yes, 2=no, blank=missing

illness started delivery facility: 1=yes (started in facility), 2=no (started at home or in community), blank=missing

MainCOD_cat – main cause of death: 0=all other causes, 1=IPRE* or prematurity, 2=severe infection, blank=missing

Svrsyndrm_rnk_illness_onset_2scat: 0=svrsyndrm_rank_illness_onset_2s=1, 1=svrsyndrm_rank_illness_onset_2s=2, 2=svrsyndrm_rank_illness_onset_2s=3, blank=svrsyndrm_rank_illness_onset_2s=missing

Variables following screening variables, e.g., if n2185 - loose/watery stools=2 (no), then n2186 - number of loose stools on worst day=blank=NA

*IPRE=intrapartum-related event (birth injury, intrapartum asphyxia)

**Five African countries variable codes**

Vagq4030 – respondent: 1=mother, 2=father, 3=grandmother, 4=grandfather, 5=aunt, 6=uncle, 7=brother, 8=sister, 9=birth attendant, 10=other male, 11=other female

Vacq1060 – birthplace (Cameroon, Nigeria, Malawi, Niger): 1=hospital, 2=other health provider/facility, 3=on route to a health provider/facility, 4=home, 5=other, 9=DK

Vacq1060 – birthplace (Tanzania): 1=hospital, 2=health center/dispensary, 3=private doctor/clinic, 4=home (with community nurse/midwife), 5=home (without community nurse/midwife), 6=on route to health provider/facility, 7=other, 9=DK

Vacq1220 – place of child’s death (Cameroon, Nigeria, Malawi, Niger): 1=hospital, 2=other health provider/facility, 3=on route to a health provider/facility, 4=home, 5=other, 9=DK

Vacq1220 – place of child’s death (Tanzania): 1=hospital, 2=health center/dispensary, 3=private doctor/clinic, 4=home (with community nurse/midwife), 5=home (without community nurse/midwife), 6=on route to health provider/facility, 7=other, 9=DK

Vacq1090 – child’s sex: 1=male, 2=female, 9=DK

Vagq4120 – floor material: 1=natural/mud, 2=cement, 3=wood, 4=tiles, 5=other, 9=DK

Vacq3080 – minutes after birth cried: 1=within 5 minutes, 2=within 6-30 minutes, 3=>30 minutes, 4=never, 9=DK

Vacq3450 – number of loose stools (on worst day): (N)umber recorded, 99=DK

Mothersage_at_childsdeath: (N)umber recorded, blank=missing

Motherschoolyears: (N)umber recorded, blank=missing

Hourstravel_to_usualfacility2: (N)umber recorded, blank=missing

Saq602a_01 – 1^st^ symptom to saq602a_14 – 14^th^ symptom: names and codes correspond to symptoms’ order of onset and their codes, e.g., ‘saq602a_03 – 3^rd^ symptom’ is the third symptom that occurred during the illness and the code ‘3330’ points to symptom ‘vacq3330 – unresponsive’ _

Saq602b_01 – illness day of 1^st^ symptom to saq602b_14 – illness day of 14^th^ symptom: names and codes correspond to the illness day on which symptoms were first noted, e.g., ‘saq602b_03 – illness day of 3^rd^ symptom’ is the illness day on which the third symptom that occurred during the illness was first noted

Saq6030_1 – feeding at illness onset: 1=normally 2=poorly, 3=not at all, 9=DK

Saq6030_3 – activity at illness onset: 1=normal, 2=less active, 3=not moving, 9=DK

svrsyndrm_rank_illness_onset_2s: 1=no/mild illness, 2=moderate illness, 3=severe illness, blank=missing

formalcare – received any formal healthcare during illness: 1=yes, 2=no, blank=missing

illness started delivery facility: 1=yes (started in facility), 2=no (started at home or in community), blank=missing

IMCI_urgent_day1count: (N)umber of IMCI urgent referral illness signs present on illness day-1, blank=missing

VASA_severe_day1count: (N)umber of IMCI and VASA severe illness signs present on illness day-1, blank=missing

IMCI_3levels_day1 - illness severity by IMCI signs on illness day-1: 1=mild, 2=moderate, 3=urgent referral, blank=missing

VASA_3levels_day1 - illness severity by IMCI and VASA signs on illness day-1: 1=mild, 2=moderate, 3=severe, blank=missing

MainCOD_cat – main cause of death: 0=all other causes, 1=IPRE* or prematurity, 2=severe infection, blank=missing

Svrsyndrm_rnk_illness_onset_2scat: 0=svrsyndrm_rank_illness_onset_2s=1, 1=svrsyndrm_rank_illness_onset_2s=2, 2=svrsyndrm_rank_illness_onset_2s=3, blank=svrsyndrm_rank_illness_onset_2s=missing

Variables following screening variables, e.g., if n2185 - loose/watery stools=2 (no), then n2186 - number of loose stools on worst day=blank=NA

*IPRE=intrapartum-related event (birth injury, intrapartum asphyxia)

**Table 5 and Annex table 7 logistic regressions**

proc surveylogistic data=NN_vasa_MZ_illness_severity; weight standardizedweight_NN_MZ; strata Province; cluster ape_code;

Class MainCOD_cat (ref='0') svrsyndrm_rnk_illnss_onset_2scat (ref='0') / param=ref; MODEL formalcare_log (event='1') = MainCOD_cat ageatdeath_onlydays svrsyndrm_rnk_illnss_onset_2scat hourstravel_to_usualfacility2 mothersage_at_childsdeath motherschoolyears;

**title 'Mozambique illness severity logistic model';**

proc surveylogistic data=illness_severity; strata district; cluster cluster;

Class MainCOD_cat (ref='0') svrsyndrm_rnk_illnss_onset_2scat (ref='0') / param=ref; MODEL formalcare_log (event='1') = MainCOD_cat ageatdeath_onlydays svrsyndrm_rnk_illnss_onset_2scat hourstravel_to_usualfacility2 mothersage_at_childsdeath motherschoolyears;

**title 'Pakistan illness severity logistic model';**

proc logistic data=NN_vasa_Cameroon_svr_at_onset;

Class MainCOD_cat (ref='0') svrsyndrm_rnk_illnss_onset_2scat (ref='0') / param=ref; MODEL formalcare_log (event='1') = MainCOD_cat ageatdeath_onlydays svrsyndrm_rnk_illnss_onset_2scat hourstravel_to_usualfacility2 mothersage_at_childsdeath motherschoolyears;

**title 'Cameroon illness severity logistic model';**

proc surveylogistic data=NN_vasa_Nigeria_svr_at_onset; weight standardizedweight723_NG; stratum StateRegion urbrur; cluster cluster;

Class MainCOD_cat (ref='0') svrsyndrm_rnk_illnss_onset_2scat (ref='0') / param=ref; MODEL formalcare_log (event='1') = MainCOD_cat ageatdeath_onlydays svrsyndrm_rnk_illnss_onset_2scat hourstravel_to_usualfacility2 mothersage_at_childsdeath motherschoolyears;

**title 'Nigeria illness severity logistic model';**

proc surveylogistic data=NN_vasa_Malawi_svr_at_onset; weight standardizedweight320_MW; cluster cluster;

Class MainCOD_cat (ref='0') svrsyndrm_rnk_illnss_onset_2scat (ref='0') / param=ref; MODEL formalcare_log (event='1') = MainCOD_cat ageatdeath_onlydays svrsyndrm_rnk_illnss_onset_2scat hourstravel_to_usualfacility2 mothersage_at_childsdeath motherschoolyears;

**title 'Malawi illness severity logistic model';**

proc surveylogistic data=NN_vasa_Niger_svr_at_onset; weight standardizedweight453_NI; stratum StateRegion urbrur; cluster cluster;

Class MainCOD_cat (ref='0') svrsyndrm_rnk_illnss_onset_2scat (ref='0') / param=ref; MODEL formalcare_log (event='1') = MainCOD_cat ageatdeath_onlydays svrsyndrm_rnk_illnss_onset_2scat hourstravel_to_usualfacility2 mothersage_at_childsdeath motherschoolyears;

**title 'Niger illness severity logistic model';**

proc surveylogistic data=NN_vasa_Tanzania_svr_at_onset; weight standardizedweight228_TZ; stratum StateRegion urbrur; cluster cluster;

Class MainCOD_cat (ref='0') svrsyndrm_rnk_illnss_onset_2scat (ref='0') / param=ref; MODEL formalcare_log (event='1') = MainCOD_cat ageatdeath_onlydays svrsyndrm_rnk_illnss_onset_2scat hourstravel_to_usualfacility2 mothersage_at_childsdeath motherschoolyears;

**title 'Tanzania illness severity logistic model';**

**SAS code to create neonatal IMCI- and VASA_3levels_day1 (only for African countries)**

* ------------------------------------------------------------------------------------------------;

* --------------------------------IMCI and IMCI-VASA illness severity-----------------------------;

* ------------------------------------------------------------------------------------------------;

/*

* IMCI_urgent_referral IMCI_urgent_referral_day1 VASA_severe_illness VASA_severe_illness_day1

IMCI_other_illness IMCI_other_illness_day1 VASA_other_illness VASA_other_illness_day1;

* Up to 2 months WHO IMCI severe illness requiring urgent referral;

* 'Not able to feed at all or feeding poorly' was newly added to the 2019 IMCI and grunting, bulging fontanelle

and umbilical redness extending to the skin were removed (I moved the last three to the IMCI-VASA severe illness)

* Possible serious bacterial infection (1 of the following) (URGENT REFERRAL):

* not suckling (vacq3110=2=not able to suckle normally first day of life or vacq3130=1=stopped being able to suckle normally)

* not able to feed at all (saq6030_1=3): the 2019 IMCI also includes 'not sucking effectively' as a sign of feeding problem (moderate illness), but this is described as ineffective positioning, attachment, etc., not a physiological problem (keep 'not able to feed' here and include 'feeding poorly' with possibly severe illness)

* convulsions (vacq3250=1)

* fast breathing in infants <7 days old (vacq3200=1 and ageatdeath=2)

* neonate's age at death:

if vacq125u=1 and vacq125n<7 and vacq125n^=99 and vacq125n^=. then ageatdeath=2; * 0-6 days old;

if vacq125u=1 and vacq125n>6 and vacq125n<28 then ageatdeath=3; * 7-27 days old;

* the age requirement was newly added to the 2019 IMCI

* severe chest indrawing (vacq3230=1)

* fever or hypothermia (vacq3260=1 or vacq3290=1)

* less than normal movement (saq6030_3 in (2,3))

* IMCI characterizes this as 'movement only when stimulated or no movement at all'

* severe jaundice (any of the below)

* any jaundice in infant <24 hours old (yellow skin or eyes: vacq3470=1 or vacq3480=1 and ageatdeath_days=0)

* yellow palms or soles at any age (VASA includes only 'yellow skin': vacq3470=1, so do not use this sign)

* severe dehydration (2 of the following) (URGENT REFERRAL for IV THERAPY)

* movement only when stimulated or no movement at all (saq6030_3 in (2,3))

* sunken eyes (not in VASA)

* skin pinch goes back very slowly (not in VASA)

* very low weight for age

* weight <2kg in infants <7 days old (not in VASA)

* feeding problem (1 of the following) (URGENT REFERRAL)

* not able to feed (saq6030_1=3)

* no attachment to breast (not in VASA)

*----------------------------------------------------------------------------------;

* Up to 2 months WHO IMCI non-severe illness;

* Pneumonia

* fast breathing in infants 7-27 days old (vacq3200=1 and ageatdeath=3)

* the age requirement was newly added to the 2019 IMCI

* Local bacterial infection

* red umbilicus or draining pus (vacq3350=1 or vacq3360=1)

* skin pustules (VASA: skin bumps with pus or a single large area with pus: vacq3380=1)

* Jaundice

* jaundice appearing after 24 hours of age AND palms or soles not yellow (not in VASA)

* Some dehydration (2 of the following)

* restless, irritable (not in VASA)

* sunken eyes (not in VASA)

* skin pinch goes back slowly (not in VASA)

* HIV infection

* positive virological test (not in VASA)

* mother is HIV positive AND infant breastfed less than 6 weeks prior has negative virological test (not in VASA)

* mother is HIV positive and infant not yet tested (vacq5180=1) (but this is not an illness sign, so do not include)

* Feeding problem or low weight

* not well attached to breast (not in VASA)

* not suckling effectively (VASA - feeding poorly: saq6030_1=2)

* the 2019 IMCI also includes 'not able to feed at all or not feeding well' as signs of very severe disease (keep 'not feeding well' here and include 'not able to feed' with very severe disease)

* less than 8 breastfeeds in 24 hours (not in VASA)

* receives other foods or drinks (for clinical assessment, not useful for BMGF analysis)

* low weight for age (not in VASA)

* thrush (not in VASA);

*/

* 1. WHO IMCI possible serious bacterial infection or feeding problem requiring URGENT REFERRAL (signs during the entire illness, except saq6030 is when illness was first noticed: did not include this at time decided to seek care because this would bias the analysis of illness signs associated with careseeking) (1 of the following signs);

if (vacq3250=1 or (vacq3200=1 and ageatdeath=2) or vacq3230=1 or vacq3240=1 or vacq3340=1 or vacq3370=1 or vacq3260=1 or vacq3290=1 or vacq3320=1 or vacq3330=1 or saq6030_3 in (2,3) or saq6030_1=3 or vacq3110=2 or vacq3130=1 or ((vacq3470=1 or vacq3480=1) and ageatdeath_days=0))

then IMCI_urgent_referral=1; else IMCI_urgent_referral=2;

* fast breathing and age at death = 0-6 days or 7-27 days);

if saq602a_01=3200 and ageatdeath=2 then saq602a_01=32002;

else if saq602a_01=3200 and ageatdeath=3 then saq602a_01=32003;

if saq602a_02=3200 and ageatdeath=2 then saq602a_02=32002;

else if saq602a_02=3200 and ageatdeath=3 then saq602a_02=32003;

if saq602a_03=3200 and ageatdeath=2 then saq602a_03=32002;

else if saq602a_03=3200 and ageatdeath=3 then saq602a_03=32003;

if saq602a_04=3200 and ageatdeath=2 then saq602a_04=32002;

else if saq602a_04=3200 and ageatdeath=3 then saq602a_04=32003;

if saq602a_05=3200 and ageatdeath=2 then saq602a_05=32002;

else if saq602a_05=3200 and ageatdeath=3 then saq602a_05=32003;

if saq602a_06=3200 and ageatdeath=2 then saq602a_06=32002;

else if saq602a_06=3200 and ageatdeath=3 then saq602a_06=32003;

if saq602a_07=3200 and ageatdeath=2 then saq602a_07=32002;

else if saq602a_07=3200 and ageatdeath=3 then saq602a_07=32003;

if saq602a_08=3200 and ageatdeath=2 then saq602a_08=32002;

else if saq602a_08=3200 and ageatdeath=3 then saq602a_08=32003;

if saq602a_09=3200 and ageatdeath=2 then saq602a_09=32002;

else if saq602a_09=3200 and ageatdeath=3 then saq602a_09=32003;

if saq602a_10=3200 and ageatdeath=2 then saq602a_10=32002;

else if saq602a_10=3200 and ageatdeath=3 then saq602a_10=32003;

if saq602a_11=3200 and ageatdeath=2 then saq602a_11=32002;

else if saq602a_11=3200 and ageatdeath=3 then saq602a_11=32003;

if saq602a_12=3200 and ageatdeath=2 then saq602a_12=32002;

else if saq602a_12=3200 and ageatdeath=3 then saq602a_12=32003;

if saq602a_13=3200 and ageatdeath=2 then saq602a_13=32002;

else if saq602a_13=3200 and ageatdeath=3 then saq602a_13=32003;

if saq602a_14=3200 and ageatdeath=2 then saq602a_14=32002;

else if saq602a_14=3200 and ageatdeath=3 then saq602a_14=32003;

* yellow skin or eyes and age at death = 0 days;

if (saq602a_01=3470 or saq602a_01=3480) and ageatdeath_days=0 then saq602a_01=347080;

else if (saq602a_01=3470 or saq602a_01=3480) and ageatdeath_days>0 then saq602a_01=.;

if (saq602a_02=3470 or saq602a_02=3480) and ageatdeath_days=0 then saq602a_02=347080;

else if (saq602a_02=3470 or saq602a_02=3480) and ageatdeath_days>0 then saq602a_02=.;

if (saq602a_03=3470 or saq602a_03=3480) and ageatdeath_days=0 then saq602a_03=347080;

else if (saq602a_03=3470 or saq602a_03=3480) and ageatdeath_days>0 then saq602a_03=.;

if (saq602a_04=3470 or saq602a_04=3480) and ageatdeath_days=0 then saq602a_04=347080;

else if (saq602a_04=3470 or saq602a_04=3480) and ageatdeath_days>0 then saq602a_04=.;

if (saq602a_05=3470 or saq602a_05=3480) and ageatdeath_days=0 then saq602a_05=347080;

else if (saq602a_05=3470 or saq602a_05=3480) and ageatdeath_days>0 then saq602a_05=.;

if (saq602a_06=3470 or saq602a_06=3480) and ageatdeath_days=0 then saq602a_06=347080;

else if (saq602a_06=3470 or saq602a_06=3480) and ageatdeath_days>0 then saq602a_06=.;

if (saq602a_07=3470 or saq602a_07=3480) and ageatdeath_days=0 then saq602a_07=347080;

else if (saq602a_07=3470 or saq602a_07=3480) and ageatdeath_days>0 then saq602a_07=.;

if (saq602a_08=3470 or saq602a_08=3480) and ageatdeath_days=0 then saq602a_08=347080;

else if (saq602a_08=3470 or saq602a_08=3480) and ageatdeath_days>0 then saq602a_08=.;

if (saq602a_09=3470 or saq602a_09=3480) and ageatdeath_days=0 then saq602a_09=347080;

else if (saq602a_09=3470 or saq602a_09=3480) and ageatdeath_days>0 then saq602a_09=.;

if (saq602a_10=3470 or saq602a_10=3480) and ageatdeath_days=0 then saq602a_10=347080;

else if (saq602a_10=3470 or saq602a_10=3480) and ageatdeath_days>0 then saq602a_10=.;

if (saq602a_11=3470 or saq602a_11=3480) and ageatdeath_days=0 then saq602a_11=347080;

else if (saq602a_11=3470 or saq602a_11=3480) and ageatdeath_days>0 then saq602a_11=.;

if (saq602a_12=3470 or saq602a_12=3480) and ageatdeath_days=0 then saq602a_12=347080;

else if (saq602a_12=3470 or saq602a_12=3480) and ageatdeath_days>0 then saq602a_12=.;

if (saq602a_13=3470 or saq602a_13=3480) and ageatdeath_days=0 then saq602a_13=347080;

else if (saq602a_13=3470 or saq602a_13=3480) and ageatdeath_days>0 then saq602a_13=.;

if (saq602a_14=3470 or saq602a_14=3480) and ageatdeath_days=0 then saq602a_14=347080;

else if (saq602a_14=3470 or saq602a_14=3480) and ageatdeath_days>0 then saq602a_14=.;

* 2. WHO IMCI possible serious_day1 bacterial infection or feeding problem requiring URGENT REFERRAL (signs reported on illness day 1) (1 of the following signs);

if ((saq602a_01 in (3250,32002,3230,3260,3290,3110,3130,347080) and saq602b_01=1) or

(saq602a_02 in (3250,32002,3230,3260,3290,3110,3130,347080) and saq602b_02=1) or

(saq602a_03 in (3250,32002,3230,3260,3290,3110,3130,347080) and saq602b_03=1) or

(saq602a_04 in (3250,32002,3230,3260,3290,3110,3130,347080) and saq602b_04=1) or

(saq602a_05 in (3250,32002,3230,3260,3290,3110,3130,347080) and saq602b_05=1) or

(saq602a_06 in (3250,32002,3230,3260,3290,3110,3130,347080) and saq602b_06=1) or

(saq602a_07 in (3250,32002,3230,3260,3290,3110,3130,347080) and saq602b_07=1) or

(saq602a_08 in (3250,32002,3230,3260,3290,3110,3130,347080) and saq602b_08=1) or

(saq602a_09 in (3250,32002,3230,3260,3290,3110,3130,347080) and saq602b_09=1) or

(saq602a_10 in (3250,32002,3230,3260,3290,3110,3130,347080) and saq602b_10=1) or

(saq602a_11 in (3250,32002,3230,3260,3290,3110,3130,347080) and saq602b_11=1) or

(saq602a_12 in (3250,32002,3230,3260,3290,3110,3130,347080) and saq602b_12=1) or

(saq602a_13 in (3250,32002,3230,3260,3290,3110,3130,347080) and saq602b_13=1) or

(saq602a_14 in (3250,32002,3230,3260,3290,3110,3130,347080) and saq602b_14=1) or

saq6030_3 in (2,3) or saq6030_1=3) then IMCI_urgent_referral_day1=1; else IMCI_urgent_referral_day1=2;

* Count of IMCI day-1 urgent referral illness signs;

if (saq602a_01 in (3250,32002,3230,3260,3290,3110,3130,347080) and saq602b_01=1) then IMCI_urgent_day1count1=1; else IMCI_urgent_day1count1=0;

if (saq602a_02 in (3250,32002,3230,3260,3290,3110,3130,347080) and saq602b_02=1) then IMCI_urgent_day1count2=1; else IMCI_urgent_day1count2=0;

if (saq602a_03 in (3250,32002,3230,3260,3290,3110,3130,347080) and saq602b_03=1) then IMCI_urgent_day1count3=1; else IMCI_urgent_day1count3=0;

if (saq602a_04 in (3250,32002,3230,3260,3290,3110,3130,347080) and saq602b_04=1) then IMCI_urgent_day1count4=1; else IMCI_urgent_day1count4=0;

if (saq602a_05 in (3250,32002,3230,3260,3290,3110,3130,347080) and saq602b_05=1) then IMCI_urgent_day1count5=1; else IMCI_urgent_day1count5=0;

if (saq602a_06 in (3250,32002,3230,3260,3290,3110,3130,347080) and saq602b_06=1) then IMCI_urgent_day1count6=1; else IMCI_urgent_day1count6=0;

if (saq602a_07 in (3250,32002,3230,3260,3290,3110,3130,347080) and saq602b_07=1) then IMCI_urgent_day1count7=1; else IMCI_urgent_day1count7=0;

if (saq602a_08 in (3250,32002,3230,3260,3290,3110,3130,347080) and saq602b_08=1) then IMCI_urgent_day1count8=1; else IMCI_urgent_day1count8=0;

if (saq602a_09 in (3250,32002,3230,3260,3290,3110,3130,347080) and saq602b_09=1) then IMCI_urgent_day1count9=1; else IMCI_urgent_day1count9=0;

if (saq602a_10 in (3250,32002,3230,3260,3290,3110,3130,347080) and saq602b_10=1) then IMCI_urgent_day1count10=1; else IMCI_urgent_day1count10=0;

if (saq602a_11 in (3250,32002,3230,3260,3290,3110,3130,347080) and saq602b_11=1) then IMCI_urgent_day1count11=1; else IMCI_urgent_day1count11=0;

if (saq602a_12 in (3250,32002,3230,3260,3290,3110,3130,347080) and saq602b_12=1) then IMCI_urgent_day1count12=1; else IMCI_urgent_day1count12=0;

if (saq602a_13 in (3250,32002,3230,3260,3290,3110,3130,347080) and saq602b_13=1) then IMCI_urgent_day1count13=1; else IMCI_urgent_day1count13=0;

if (saq602a_14 in (3250,32002,3230,3260,3290,3110,3130,347080) and saq602b_14=1) then IMCI_urgent_day1count14=1; else IMCI_urgent_day1count14=0;

if saq6030_3 in (2,3) then IMCI_urgent_day1count15=1; else IMCI_urgent_day1count15=0;

if saq6030_1=3 then IMCI_urgent_day1count16=1; else IMCI_urgent_day1count16=0;

IMCI_urgent_day1count=(IMCI_urgent_day1count1+IMCI_urgent_day1count2+IMCI_urgent_day1count3+IMCI_urgent_day1count4+IMCI_urgent_day1count5+IMCI_urgent_day1count6+IMCI_urgent_day1count7+IMCI_urgent_day1count8+IMCI_urgent_day1count9+IMCI_urgent_day1count10+IMCI_urgent_day1count11+IMCI_urgent_day1count12+IMCI_urgent_day1count13+IMCI_urgent_day1count14+IMCI_urgent_day1count15+IMCI_urgent_day1count16);

* 3. VASA severe illness signs (IMCI signs + other signs in the VASA, all during the entire illness);

* grunting, bulging fontanelle, umbilical redness extending to skin, lethargic and unconscious were formerly IMCI signs of very severe illness but they were removed from the 2019 IMCI and I've moved them to the VASA severe illness signs;

* vacq3040=not able to breathe immediately after birth, vacq3080 in (2,3,4)=first cried >30 minutes after birth or never, vacq3090=stopped being able to cry, vacq3160=not able to open mouth when stopped suckling, vacq3390=ulcers/pits, vacq3410=areas of skin turned black, vacq3420=bleeding from anywhere, grunting (vacq3240=1), bulging fontanelle (vacq3340=1), umbilical redness extending to skin (vacq3370=1), lethargic or unconscious (vacq3320=1 or vacq3330=1);

if (IMCI_urgent_referral=1 or vacq3040=1 or vacq3080 in (2,3,4) or vacq3090=1 or vacq3160=2 or vacq3390=1 or vacq3410=1 or vacq3420=1 or vacq3240=1 or vacq3340=1 or vacq3370=1 or vacq3320=1 or vacq3330=1) then VASA_severe_illness=1; else VASA_severe_illness=2;

* 4. VASA severe illness signs_day1 (IMCI signs + other signs in the VASA reported on illness day-1);

* vacq3040=not able to breathe immediately after birth, vacq3080 in (2,3,4)=first cried >5 minutes after birth or never, vacq3090=stopped being able to cry, vacq3160=not able to open mouth when stopped suckling, vacq3390=ulcers/pits, vacq3410=areas of skin turned black, vacq3420=bleeding from anywhere, vacq3460=vomit everything;

if (IMCI_urgent_referral_day1=1 or

(saq602a_01 in (3040,3080,3090,3160,3390,3410,3420,3460,3240,3340,3370,3320,3330) and saq602b_01=1) or

(saq602a_02 in (3040,3080,3090,3160,3390,3410,3420,3460,3240,3340,3370,3320,3330) and saq602b_02=1) or

(saq602a_03 in (3040,3080,3090,3160,3390,3410,3420,3460,3240,3340,3370,3320,3330) and saq602b_03=1) or

(saq602a_04 in (3040,3080,3090,3160,3390,3410,3420,3460,3240,3340,3370,3320,3330) and saq602b_04=1) or

(saq602a_05 in (3040,3080,3090,3160,3390,3410,3420,3460,3240,3340,3370,3320,3330) and saq602b_05=1) or

(saq602a_06 in (3040,3080,3090,3160,3390,3410,3420,3460,3240,3340,3370,3320,3330) and saq602b_06=1) or

(saq602a_07 in (3040,3080,3090,3160,3390,3410,3420,3460,3240,3340,3370,3320,3330) and saq602b_07=1) or

(saq602a_08 in (3040,3080,3090,3160,3390,3410,3420,3460,3240,3340,3370,3320,3330) and saq602b_08=1) or

(saq602a_09 in (3040,3080,3090,3160,3390,3410,3420,3460,3240,3340,3370,3320,3330) and saq602b_09=1) or

(saq602a_10 in (3040,3080,3090,3160,3390,3410,3420,3460,3240,3340,3370,3320,3330) and saq602b_10=1) or

(saq602a_11 in (3040,3080,3090,3160,3390,3410,3420,3460,3240,3340,3370,3320,3330) and saq602b_11=1) or

(saq602a_12 in (3040,3080,3090,3160,3390,3410,3420,3460,3240,3340,3370,3320,3330) and saq602b_12=1) or

(saq602a_13 in (3040,3080,3090,3160,3390,3410,3420,3460,3240,3340,3370,3320,3330) and saq602b_13=1) or

(saq602a_14 in (3040,3080,3090,3160,3390,3410,3420,3460,3240,3340,3370,3320,3330) and saq602b_14=1))

then VASA_severe_illness_day1=1; else VASA_severe_illness_day1=2;

* Count of IMCI day-1 urgent referral + VASA day-1 severe illness signs;

if (saq602a_01 in (3250,32002,3230,3260,3290,3110,3130,347080,3040,3080,3090,3160,3390,3410,3420,3460,3240,3340,3370,3320,3330) and saq602b_01=1) then VASA_severe_day1count1=1; else VASA_severe_day1count1=0;

if (saq602a_02 in (3250,32002,3230,3260,3290,3110,3130,347080,3040,3080,3090,3160,3390,3410,3420,3460,3240,3340,3370,3320,3330) and saq602b_02=1) then VASA_severe_day1count2=1; else VASA_severe_day1count2=0;

if (saq602a_03 in (3250,32002,3230,3260,3290,3110,3130,347080,3040,3080,3090,3160,3390,3410,3420,3460,3240,3340,3370,3320,3330) and saq602b_03=1) then VASA_severe_day1count3=1; else VASA_severe_day1count3=0;

if (saq602a_04 in (3250,32002,3230,3260,3290,3110,3130,347080,3040,3080,3090,3160,3390,3410,3420,3460,3240,3340,3370,3320,3330) and saq602b_04=1) then VASA_severe_day1count4=1; else VASA_severe_day1count4=0;

if (saq602a_05 in (3250,32002,3230,3260,3290,3110,3130,347080,3040,3080,3090,3160,3390,3410,3420,3460,3240,3340,3370,3320,3330) and saq602b_05=1) then VASA_severe_day1count5=1; else VASA_severe_day1count5=0;

if (saq602a_06 in (3250,32002,3230,3260,3290,3110,3130,347080,3040,3080,3090,3160,3390,3410,3420,3460,3240,3340,3370,3320,3330) and saq602b_06=1) then VASA_severe_day1count6=1; else VASA_severe_day1count6=0;

if (saq602a_07 in (3250,32002,3230,3260,3290,3110,3130,347080,3040,3080,3090,3160,3390,3410,3420,3460,3240,3340,3370,3320,3330) and saq602b_07=1) then VASA_severe_day1count7=1; else VASA_severe_day1count7=0;

if (saq602a_08 in (3250,32002,3230,3260,3290,3110,3130,347080,3040,3080,3090,3160,3390,3410,3420,3460,3240,3340,3370,3320,3330) and saq602b_08=1) then VASA_severe_day1count8=1; else VASA_severe_day1count8=0;

if (saq602a_09 in (3250,32002,3230,3260,3290,3110,3130,347080,3040,3080,3090,3160,3390,3410,3420,3460,3240,3340,3370,3320,3330) and saq602b_09=1) then VASA_severe_day1count9=1; else VASA_severe_day1count9=0;

if (saq602a_10 in (3250,32002,3230,3260,3290,3110,3130,347080,3040,3080,3090,3160,3390,3410,3420,3460,3240,3340,3370,3320,3330) and saq602b_10=1) then VASA_severe_day1count10=1; else VASA_severe_day1count10=0;

if (saq602a_11 in (3250,32002,3230,3260,3290,3110,3130,347080,3040,3080,3090,3160,3390,3410,3420,3460,3240,3340,3370,3320,3330) and saq602b_11=1) then VASA_severe_day1count11=1; else VASA_severe_day1count11=0;

if (saq602a_12 in (3250,32002,3230,3260,3290,3110,3130,347080,3040,3080,3090,3160,3390,3410,3420,3460,3240,3340,3370,3320,3330) and saq602b_12=1) then VASA_severe_day1count12=1; else VASA_severe_day1count12=0;

if (saq602a_13 in (3250,32002,3230,3260,3290,3110,3130,347080,3040,3080,3090,3160,3390,3410,3420,3460,3240,3340,3370,3320,3330) and saq602b_13=1) then VASA_severe_day1count13=1; else VASA_severe_day1count13=0;

if (saq602a_14 in (3250,32002,3230,3260,3290,3110,3130,347080,3040,3080,3090,3160,3390,3410,3420,3460,3240,3340,3370,3320,3330) and saq602b_14=1) then VASA_severe_day1count14=1; else VASA_severe_day1count14=0;

if saq6030_3 in (2,3) then VASA_severe_day1count15=1; else VASA_severe_day1count15=0;

if saq6030_1=3 then VASA_severe_day1count16=1; else VASA_severe_day1count16=0;

VASA_severe_day1count=(VASA_severe_day1count1+VASA_severe_day1count2+VASA_severe_day1count3+VASA_severe_day1count4+VASA_severe_day1count5+VASA_severe_day1count6+VASA_severe_day1count7+VASA_severe_day1count8+VASA_severe_day1count9+VASA_severe_day1count10+VASA_severe_day1count11+VASA_severe_day1count12+VASA_severe_day1count13+VASA_severe_day1count14+VASA_severe_day1count15+VASA_severe_day1count16);

* 5. WHO IMCI other illness (signs during the entire illness, except saq6030 is when illness was first noticed:

did not include this at time decided to seek care because this would bias the analysis of illness signs

associated with careseeking) (1 of the following signs [fast breathing in 7-27 day old added to updated IMCI]);

if (vacq3350=1 or vacq3360=1 or vacq3380=1 or (vacq3200=1 and ageatdeath=3) or saq6030_1=2)

then IMCI_other_illness=1; else IMCI_other_illness=2;

* 6. WHO IMCI other illness_day1 (signs on illness day 1):;

if ((saq602a_01 in (3350,3360,3380,32003) and saq602b_01=1) or

(saq602a_02 in (3350,3360,3380,32003) and saq602b_02=1) or

(saq602a_03 in (3350,3360,3380,32003) and saq602b_03=1) or

(saq602a_04 in (3350,3360,3380,32003) and saq602b_04=1) or

(saq602a_05 in (3350,3360,3380,32003) and saq602b_05=1) or

(saq602a_06 in (3350,3360,3380,32003) and saq602b_06=1) or

(saq602a_07 in (3350,3360,3380,32003) and saq602b_07=1) or

(saq602a_08 in (3350,3360,3380,32003) and saq602b_08=1) or

(saq602a_09 in (3350,3360,3380,32003) and saq602b_09=1) or

(saq602a_10 in (3350,3360,3380,32003) and saq602b_10=1) or

(saq602a_11 in (3350,3360,3380,32003) and saq602b_11=1) or

(saq602a_12 in (3350,3360,3380,32003) and saq602b_12=1) or

(saq602a_13 in (3350,3360,3380,32003) and saq602b_13=1) or

(saq602a_14 in (3350,3360,3380,32003) and saq602b_14=1) or saq6030_1=2)

then IMCI_other_illness_day1=1; else IMCI_other_illness_day1=2;

* 7. VASA other illness (IMCI signs + other signs in the VASA at any time during the illness);

* vacq3010=bruises/signs of injury at birth, vacq3050=difficult breathing at birth, vacq3080=1=first cried within 5 minutes after birth (did not cry immediately), vacq3450>=4=4 or more stools on worst day, vacq3170=difficult breathing;

if (IMCI_other_illness=1 or vacq3010=1 or vacq3050=1 or vacq3080=1 or (vacq3450>=4 and vacq3450<99) or

vacq3170=1) then VASA_other_illness=1; else VASA_other_illness=2;

if saq602a_01=3440 and vacq3450>=4 and vacq3450<99 then saq602a_01=3440;

else if saq602a_01=3440 and vacq3450 in (1,2,3,99) then saq602a_01=.;

if saq602a_02=3440 and vacq3450>=4 and vacq3450<99 then saq602a_02=3440;

else if saq602a_02=3440 and vacq3450 in (1,2,3,99) then saq602a_02=.;

if saq602a_03=3440 and vacq3450>=4 and vacq3450<99 then saq602a_03=3440;

else if saq602a_03=3440 and vacq3450 in (1,2,3,99) then saq602a_03=.;

if saq602a_04=3440 and vacq3450>=4 and vacq3450<99 then saq602a_04=3440;

else if saq602a_04=3440 and vacq3450 in (1,2,3,99) then saq602a_04=.;

if saq602a_05=3440 and vacq3450>=4 and vacq3450<99 then saq602a_05=3440;

else if saq602a_05=3440 and vacq3450 in (1,2,3,99) then saq602a_05=.;

if saq602a_06=3440 and vacq3450>=4 and vacq3450<99 then saq602a_06=3440;

else if saq602a_06=3440 and vacq3450 in (1,2,3,99) then saq602a_06=.;

if saq602a_07=3440 and vacq3450>=4 and vacq3450<99 then saq602a_07=3440;

else if saq602a_07=3440 and vacq3450 in (1,2,3,99) then saq602a_07=.;

if saq602a_08=3440 and vacq3450>=4 and vacq3450<99 then saq602a_08=3440;

else if saq602a_08=3440 and vacq3450 in (1,2,3,99) then saq602a_08=.;

if saq602a_09=3440 and vacq3450>=4 and vacq3450<99 then saq602a_09=3440;

else if saq602a_09=3440 and vacq3450 in (1,2,3,99) then saq602a_09=.;

if saq602a_10=3440 and vacq3450>=4 and vacq3450<99 then saq602a_10=3440;

else if saq602a_10=3440 and vacq3450 in (1,2,3,99) then saq602a_10=.;

if saq602a_11=3440 and vacq3450>=4 and vacq3450<99 then saq602a_11=3440;

else if saq602a_11=3440 and vacq3450 in (1,2,3,99) then saq602a_11=.;

if saq602a_12=3440 and vacq3450>=4 and vacq3450<99 then saq602a_12=3440;

else if saq602a_12=3440 and vacq3450 in (1,2,3,99) then saq602a_12=.;

if saq602a_13=3440 and vacq3450>=4 and vacq3450<99 then saq602a_13=3440;

else if saq602a_13=3440 and vacq3450 in (1,2,3,99) then saq602a_13=.;

if saq602a_14=3440 and vacq3450>=4 and vacq3450<99 then saq602a_14=3440;

else if saq602a_14=3440 and vacq3450 in (1,2,3,99) then saq602a_14=.;

* 8. VASA other illness_day1 (IMCI signs + other signs in the VASA on illness day-1);

* vacq3010=bruises/signs of injury at birth, vacq3050=difficult breathing at birth, vacq3080=1=first cried within 5 minutes after birth (did not cry immediately), vacq3450>=4=4 or more stools on worst day, vacq3170=difficult breathing;

if (IMCI_other_illness_day1=1 or vacq3080=1 or

(saq602a_01 in (3010,3050,3440,3170) and saq602b_01=1) or

(saq602a_02 in (3010,3050,3440,3170) and saq602b_02=1) or

(saq602a_03 in (3010,3050,3440,3170) and saq602b_03=1) or

(saq602a_04 in (3010,3050,3440,3170) and saq602b_04=1) or

(saq602a_05 in (3010,3050,3440,3170) and saq602b_05=1) or

(saq602a_06 in (3010,3050,3440,3170) and saq602b_06=1) or

(saq602a_07 in (3010,3050,3440,3170) and saq602b_07=1) or

(saq602a_08 in (3010,3050,3440,3170) and saq602b_08=1) or

(saq602a_09 in (3010,3050,3440,3170) and saq602b_09=1) or

(saq602a_10 in (3010,3050,3440,3170) and saq602b_10=1) or

(saq602a_11 in (3010,3050,3440,3170) and saq602b_11=1) or

(saq602a_12 in (3010,3050,3440,3170) and saq602b_12=1) or

(saq602a_13 in (3010,3050,3440,3170) and saq602b_13=1) or

(saq602a_14 in (3010,3050,3440,3170) and saq602b_14=1))

then VASA_other_illness_day1=1; else VASA_other_illness_day1=2;

* 3-level IMCI day-1 illness category (variable = IMCI_3levels_day1);

if IMCI_urgent_referral_day1=1 then IMCI_3levels_day1=3;

else if IMCI_other_illness_day1=1 and IMCI_urgent_referral_day1=2 then IMCI_3levels_day1=2;

else if IMCI_urgent_referral_day1=2 and IMCI_other_illness_day1=2 then IMCI_3levels_day1=1;

if IMCI_other_illness_day1=. and IMCI_urgent_referral_day1=. then IMCI_3levels_day1=.;

* 3-level IMCI-VASA day-1 illness category (variable = VASA_3levels_day1);

if VASA_severe_illness_day1=1 then VASA_3levels_day1=3;

else if VASA_other_illness_day1=1 and VASA_severe_illness_day1=2 then VASA_3levels_day1=2;

else if VASA_severe_illness_day1=2 and VASA_other_illness_day1=2 then VASA_3levels_day1=1;

if VASA_other_illness_day1=. and VASA_severe_illness_day1=. then VASA_3levels_day1=.;

* -------------------------------------------------------------------------------------------------;

* ---------------------------------IMCI and IMCI-VASA illness severity-----------------------------;

* -------------------------------------------------------------------------------------------------;

**1-11-months-old deaths_metadata**

**All countries variable codes**

Country: 1=Cameroon, 2=Nigeria, 3=Malawi, 4=Niger, 5=Tanzania

Urbrur: 1=urban, 2=rural

Yes/No variables: 1=yes, 2=no

Variables not listed below are described by their data label and their levels are self-evident

**Five African countries variable codes**

Vagq4030 – respondent: 1=mother, 2=father, 3=grandmother, 4=grandfather, 5=aunt, 6=uncle, 7=brother, 8=sister, 9=birth attendant, 10=other male, 11=other female

Vacq1060 – birthplace (Cameroon, Nigeria, Malawi, Niger): 1=hospital, 2=other health provider/facility, 3=on route to a health provider/facility, 4=home, 5=other, 9=DK

Vacq1060 – birthplace (Tanzania): 1=hospital, 2=health center/dispensary, 3=private doctor/clinic, 4=home (with community nurse/midwife), 5=home (without community nurse/midwife), 6=on route to health provider/facility, 7=other, 9=DK

Vacq1220 – place of child’s death (Cameroon, Nigeria, Malawi, Niger): 1=hospital, 2=other health provider/facility, 3=on route to a health provider/facility, 4=home, 5=other, 9=DK

Vacq1220 – place of child’s death (Tanzania): 1=hospital, 2=health center/dispensary, 3=private doctor/clinic, 4=home (with community nurse/midwife), 5=home (without community nurse/midwife), 6=on route to health provider/facility, 7=other, 9=DK

Vacq1090 – child’s sex: 1=male, 2=female, 9=DK

Vagq4120 – floor material: 1=natural/mud, 2=cement, 3=wood, 4=tiles, 5=other, 9=DK

Vacq4070 – number of loose stools (on worst day): (N)umber recorded, 99=DK

Vacq4130 – number of days cough lasted: (N)umber recorded, 0=<24 hours, 99=DK

Mothersage_at_childsdeath: (N)umber recorded, blank=missing

Motherschoolyears: (N)umber recorded, blank=missing

Hourstravel_to_usualfacility2: (N)umber recorded, blank=missing

Saq602a_01 – 1^st^ symptom to saq602a_18 – 18^th^ symptom: names and codes correspond to symptoms’ order of onset and their codes, e.g., ‘saq602a_03 – 3^rd^ symptom’ is the third symptom that occurred during the illness and the code ‘4060’ points to symptom ‘vacq4060 – loose/watery stools’ _

Saq602b_01 – illness day of 1^st^ symptom to saq602b_18 – illness day of 18^th^ symptom: names and codes correspond to the illness day on which symptoms were first noted, e.g., ‘saq602b_03 – illness day of 3^rd^ symptom’ is the illness day on which the third symptom that occurred during the illness was first noted

Saq6030_1 – feeding at illness onset: 1=normally 2=poorly, 3=not at all, 9=DK

Saq6030_3 – activity at illness onset: 1=normal, 2=less active, 3=not moving, 9=DK

svrsyndrm_rank_illness_onset_2s: 1=no/mild illness, 2=moderate illness, 3=severe illness, blank=missing

formalcare – received any formal healthcare during illness: 1=yes, 2=no, blank=missing

illness started delivery facility: 1=yes (started in facility), 2=no (started at home or in community), blank=missing

IMCI_urgent_day1count: (N)umber of IMCI urgent referral illness signs present on illness day-1, blank=missing

VASA_severe_day1count: (N)umber of IMCI and VASA severe illness signs present on illness day-1, blank=missing

IMCI_3levels_day1 - illness severity by IMCI signs on illness day-1: 1=mild, 2=moderate, 3=urgent referral, blank=missing

VASA_3levels_day1 - illness severity by IMCI and VASA signs on illness day-1: 1=mild, 2=moderate, 3=severe, blank=missing

MainCOD_infection – main cause of death: 0=all other causes, 1=severe febrile infection, blank=missing

VASA_3levels_day1cat: 0=VASA_3levels_day1=1, 1=VASA_3levels_day1=2, 2=VASA_3levels_day1=3, blank=VASA_3levels_day1=missing

Variables following screening variables, e.g., if n2185 - loose/watery stools=2 (no), then n2186 - number of loose stools on worst day=blank=NA

**Table 5 and Annex table 7 logistic regressions**

proc logistic data=Infant_vasa_CM_svr_at_onset; Class MainCOD_infection (ref='0') VASA_3levels_day1cat (ref='0') / param=ref; MODEL formalcare_log (event='1') = MainCOD_infection ageatdeath_allmonths VASA_3levels_day1cat hourstravel_to_usualfacility2 mothersage_at_childsdeath motherschoolyears;

**title 'Cameroon illness severity logistic model';**

proc surveylogistic data=Infant_vasa_Nigeria_svr_at_onset; weight standardizedweight691_NG; stratum StateRegion urbrur; cluster cluster; Class MainCOD_infection (ref='0') VASA_3levels_day1cat (ref='0') / param=ref; MODEL formalcare_log (event='1') = MainCOD_infection ageatdeath_allmonths VASA_3levels_day1cat hourstravel_to_usualfacility2 mothersage_at_childsdeath motherschoolyears;

**title 'Nigeria illness severity logistic model';**

proc surveylogistic data=Infant_vasa_Malawi_svr_at_onset; weight standardizedweight335_MW; cluster cluster;

Class MainCOD_infection (ref='0') VASA_3levels_day1cat (ref='0') / param=ref; MODEL formalcare_log (event='1') = MainCOD_infection ageatdeath_allmonths VASA_3levels_day1cat hourstravel_to_usualfacility2 mothersage_at_childsdeath motherschoolyears;

**title 'Malawi illness severity logistic model';**

proc surveylogistic data=Infant_vasa_Niger_svr_at_onset; weight standardizedweight269_NI; stratum StateRegion urbrur; cluster cluster; Class MainCOD_infection (ref='0') VASA_3levels_day1cat (ref='0') / param=ref; MODEL formalcare_log (event='1') = MainCOD_infection ageatdeath_allmonths VASA_3levels_day1cat hourstravel_to_usualfacility2 mothersage_at_childsdeath motherschoolyears;

**title 'Niger illness severity logistic model';**

proc surveylogistic data=Infant_vasa_TZ_svr_at_onset; weight standardizedweight158_TZ; stratum StateRegion urbrur; cluster cluster; Class MainCOD_infection (ref='0') VASA_3levels_day1cat (ref='0') / param=ref; MODEL formalcare_log (event='1') = MainCOD_infection ageatdeath_allmonths VASA_3levels_day1cat hourstravel_to_usualfacility2 mothersage_at_childsdeath motherschoolyears;

**title 'Tanzania illness severity logistic model';**

**SAS code to create 1-11-months-old IMCI- and VASA_3levels_day1 (only for African countries)**

* -------------------------------------------------------------------------------------------------;

* --------------------------------------Illness severity-------------------------------------------;

* -------------------------------------------------------------------------------------------------;

/*

* IMCI_urgent_referral IMCI_urgent_referral_day1 VASA_severe_illness VASA_severe_illness_day1

IMCI_other_illness IMCI_other_illness_day1 VASA_other_illness VASA_other_illness_day1;

* 2-59 months WHO IMCI Danger signs;

not able to drink/breastfeed (saq6030_1=3)

vomit everything (VASA has 'vomit' but not 'vomit everything')

convulsions (vacq4250)

lethargic (not in CHERG VASA; COMSA VASA = C3096)

unconscious (vacq4260)

* 2-59 months WHO IMCI severe illness requiring urgent referral;

* Severe pnuemonia or very severe disease (1 of the following) (URGENT REFERRAL):

* any of the above danger signs

* not able to drink/breastfeed (saq6030_1=3)

* vomit everything (COMSA VASA has 'vomit' but not 'vomit everything'; CHERG VASA has 'vomit after s/he coughed')

* convulsions (vacq4250)

* lethargic (not in CHERG VASA; COMSA VASA = C3096)

* unconscious (vacq4260)

* stridor in calm child (vacq4220)

* Diarrhea with severe dehydration (2 or more of the following) (URGENT REFERRAL for IV THERAPY)

* for CHERG analysis: include 'not able to drink or drinking poorly' only if also with diarrhea and

unconscious (vacq4060=1 and vacq4260=1 and saq6030_1 in (2,3));

* lethargic (not in CHERG VASA; COMSA VASA = C3096)

* unconscious (vacq4260)

* sunken eyes (not in VASA)

* not able to drink or drinking poorly (saq6030_1 in (2,3))

* skin pinch goes back very slowly (not in VASA)

* Severe persistent diarrhea (persistent diarrhea and dehydration: not included in analysis since no dehydration signs) (refer to hospital)

* 14 days or more (diardur>=14)

* dehydration present (signs not specified by IMCI)

* Very severe febrile disease (URGENT REFERRAL)

* any of the above danger signs

* stiff neck (vacq4280)

* Severe complicated measles (URGENT REFERRAL)

* any of the above danger signs

* clouding of the cornea (not in VASA)

* deep or extensive mouth ulcers (not in VASA)

* Mastoiditis (URGENT REFERRAL)

* tender swelling behind the ear (not in VASA)

* Severe malnutrition (URGENT REFERRAL)

* For severe malnutrition, the updated IMCI includes WFH/L or MUAC measurement, but we don't have this in the VASA so

continue use of visible severe wasting

* Updated IMCI also allows dx of severe malnutrition based on edema of both feet

* visible severe wasting (vacq4350)

* edema of both feet (VASA='swollen legs or feet': vacq4360)

* Severe anemia (URGENT REFERRAL)

* severe palmar pallor (not in VASA - include 'lack of blood or pallor' in below non-severe signs)

* ---------------------------------------------------------;

* 2-59 months WHO IMCI non-severe illness;

* Pneumonia (one or more of the following):

* chest indrawing (vacq4200)

* fast breathing (vacq4180)

* Diarrhea with some dehydration (two or more of the following):

* restless/irritable (not in VASA)

* sunken eyes (not in VASA)

* drinks eagerly/thirsty (not in VASA)

* skin pinch goes back slowly (not in VASA)

* diarrhea with 3+ stools on worst day (include in analysis even though VASA does not include dehydration signs: vacq4070>=3)

* Persistent diarrhea without dehydration (VASA: diardur 14+ days)

* Dysentery

* blood in the stool (vacq4110)

* Malaria

* fever in high malaria risk area (fever=vacq4010 (if saq3060 not=.)) (saq3060 is answered only in areas with malaria);

* new IMCI does not distinguish between high and low malaria risk areas, and requires positive malaria test for diagnosis;

* VASA does not include the data for the new IMCI criteria, so we will continue to use fever in a high malaria risk area as

* a moderate illness sign; IMCI now includes fever alone as a mild illness sign;

* Measles with eye or mouth complications

* pus drainage from the eye (not in VASA)

* mouth ulcers (not in VASA)

* Acute ear infection

* pus seen draining from the ear, reported as <14 days (not in VASA)

* ear pain (not in VASA)

* Chronic ear infection

* pus seen draining from the ear, reported as 14+ days (not in VASA)

* Uncomplicated severe acute malnutrition or moderate acute malnutrition

* The updated IMCI requires WFH/L or MUAC measurement, but we don't have this in the VASA

* The IMCI (old and updated) also advises to assess the malnourished child for a feeding problem and to follow up any problem in 7 days

* very low or low weight for height/length (not in VASA)

* poor feeding (saq6030_1=2)

* Anemia

* some palmar pallor (vacq4410 = 'lack of blood or pallor')

*/

* 3 or more loose stools on the day with the most loose stools;

if saq602a_01=4060 and vacq4070>=3 and vacq4070^=99 then saq602a_01=4060;

else if saq602a_01=4060 and vacq4070<3 then saq602a_01=.;

if saq602a_02=4060 and vacq4070>=3 and vacq4070^=99 then saq602a_02=4060;

else if saq602a_02=4060 and vacq4070<3 then saq602a_02=.;

if saq602a_03=4060 and vacq4070>=3 and vacq4070^=99 then saq602a_03=4060;

else if saq602a_03=4060 and vacq4070<3 then saq602a_03=.;

if saq602a_04=4060 and vacq4070>=3 and vacq4070^=99 then saq602a_04=4060;

else if saq602a_04=4060 and vacq4070<3 then saq602a_04=.;

if saq602a_05=4060 and vacq4070>=3 and vacq4070^=99 then saq602a_05=4060;

else if saq602a_05=4060 and vacq4070<3 then saq602a_05=.;

if saq602a_06=4060 and vacq4070>=3 and vacq4070^=99 then saq602a_06=4060;

else if saq602a_06=4060 and vacq4070<3 then saq602a_06=.;

if saq602a_07=4060 and vacq4070>=3 and vacq4070^=99 then saq602a_07=4060;

else if saq602a_07=4060 and vacq4070<3 then saq602a_07=.;

if saq602a_08=4060 and vacq4070>=3 and vacq4070^=99 then saq602a_08=4060;

else if saq602a_08=4060 and vacq4070<3 then saq602a_08=.;

if saq602a_09=4060 and vacq4070>=3 and vacq4070^=99 then saq602a_09=4060;

else if saq602a_09=4060 and vacq4070<3 then saq602a_09=.;

if saq602a_10=4060 and vacq4070>=3 and vacq4070^=99 then saq602a_10=4060;

else if saq602a_10=4060 and vacq4070<3 then saq602a_10=.;

if saq602a_11=4060 and vacq4070>=3 and vacq4070^=99 then saq602a_11=4060;

else if saq602a_11=4060 and vacq4070<3 then saq602a_11=.;

if saq602a_12=4060 and vacq4070>=3 and vacq4070^=99 then saq602a_12=4060;

else if saq602a_12=4060 and vacq4070<3 then saq602a_12=.;

if saq602a_13=4060 and vacq4070>=3 and vacq4070^=99 then saq602a_13=4060;

else if saq602a_13=4060 and vacq4070<3 then saq602a_13=.;

if saq602a_14=4060 and vacq4070>=3 and vacq4070^=99 then saq602a_14=4060;

else if saq602a_14=4060 and vacq4070<3 then saq602a_14=.;

if saq602a_15=4060 and vacq4070>=3 and vacq4070^=99 then saq602a_15=4060;

else if saq602a_15=4060 and vacq4070<3 then saq602a_15=.;

if saq602a_16=4060 and vacq4070>=3 and vacq4070^=99 then saq602a_16=4060;

else if saq602a_16=4060 and vacq4070<3 then saq602a_16=.;

if saq602a_17=4060 and vacq4070>=3 and vacq4070^=99 then saq602a_17=4060;

else if saq602a_17=4060 and vacq4070<3 then saq602a_17=.;

if saq602a_18=4060 and vacq4070>=3 and vacq4070^=99 then saq602a_18=4060;

else if saq602a_18=4060 and vacq4070<3 then saq602a_18=.;

* fever in an area with malaria;

if saq602a_01=4010 and saq3060 in (1,2,3,9) then saq602a_01=4010;

else if saq602a_01=4010 and saq3060=. then saq602a_01=.;

if saq602a_02=4010 and saq3060 in (1,2,3,9) then saq602a_02=4010;

else if saq602a_02=4010 and saq3060=. then saq602a_02=.;

if saq602a_03=4010 and saq3060 in (1,2,3,9) then saq602a_03=4010;

else if saq602a_03=4010 and saq3060=. then saq602a_03=.;

if saq602a_04=4010 and saq3060 in (1,2,3,9) then saq602a_04=4010;

else if saq602a_04=4010 and saq3060=. then saq602a_04=.;

if saq602a_05=4010 and saq3060 in (1,2,3,9) then saq602a_05=4010;

else if saq602a_05=4010 and saq3060=. then saq602a_05=.;

if saq602a_06=4010 and saq3060 in (1,2,3,9) then saq602a_06=4010;

else if saq602a_06=4010 and saq3060=. then saq602a_06=.;

if saq602a_07=4010 and saq3060 in (1,2,3,9) then saq602a_07=4010;

else if saq602a_07=4010 and saq3060=. then saq602a_07=.;

if saq602a_08=4010 and saq3060 in (1,2,3,9) then saq602a_08=4010;

else if saq602a_08=4010 and saq3060=. then saq602a_08=.;

if saq602a_09=4010 and saq3060 in (1,2,3,9) then saq602a_09=4010;

else if saq602a_09=4010 and saq3060=. then saq602a_09=.;

if saq602a_10=4010 and saq3060 in (1,2,3,9) then saq602a_10=4010;

else if saq602a_10=4010 and saq3060=. then saq602a_10=.;

if saq602a_11=4010 and saq3060 in (1,2,3,9) then saq602a_11=4010;

else if saq602a_11=4010 and saq3060=. then saq602a_11=.;

if saq602a_12=4010 and saq3060 in (1,2,3,9) then saq602a_12=4010;

else if saq602a_12=4010 and saq3060=. then saq602a_12=.;

if saq602a_13=4010 and saq3060 in (1,2,3,9) then saq602a_13=4010;

else if saq602a_13=4010 and saq3060=. then saq602a_13=.;

if saq602a_14=4010 and saq3060 in (1,2,3,9) then saq602a_14=4010;

else if saq602a_14=4010 and saq3060=. then saq602a_14=.;

if saq602a_15=4010 and saq3060 in (1,2,3,9) then saq602a_15=4010;

else if saq602a_15=4010 and saq3060=. then saq602a_15=.;

if saq602a_16=4010 and saq3060 in (1,2,3,9) then saq602a_16=4010;

else if saq602a_16=4010 and saq3060=. then saq602a_16=.;

if saq602a_17=4010 and saq3060 in (1,2,3,9) then saq602a_17=4010;

else if saq602a_17=4010 and saq3060=. then saq602a_17=.;

if saq602a_18=4010 and saq3060 in (1,2,3,9) then saq602a_18=4010;

else if saq602a_18=4010 and saq3060=. then saq602a_18=.;

if vacq4090=1 and vacq4080^=99 then diardur=vacq4080;

else if vacq4090=2 and vacq4080^=99 and vacq4100^=99 then diardur=vacq4080-vacq4100;

* 1. WHO IMCI danger sign or severe illness requiring URGENT REFERRAL (signs during the entire

illness, except saq6030 is when illness was first noticed: did not include this at time decided to seek care

because this would bias the analysis of illness signs associated with careseeking) (1 of the following signs);

if (vacq4250=1 or vacq4260=1 or saq6030_1=3 or vacq4200=1 or vacq4220=1 or vacq4280=1 or vacq4350=1 or vacq4360=1 or

(vacq4060=1 and vacq4260=1 and saq6030_1 in (2,3))) then IMCI_urgent_referral=1; else IMCI_urgent_referral=2;

* 2. WHO IMCI possible serious_day1 bacterial infection or feeding problem requiring URGENT REFERRAL (signs reported on

illness day 1) (1 of the following signs) (do not include 'diarrhea and unconscious and feeding poorly or not at all'

because this would require massive coding, i.e., all possible combinations of diarrhea in any of signs 1-18 and

unconscious in any of signs 1-18 and feeding poorly or not at all at illness onset);

if ((saq602a_01 in (4250,4260,4220,4280,4350,4360) and saq602b_01=1) or

(saq602a_02 in (4250,4260,4220,4280,4350,4360) and saq602b_02=1) or

(saq602a_03 in (4250,4260,4220,4280,4350,4360) and saq602b_03=1) or

(saq602a_04 in (4250,4260,4220,4280,4350,4360) and saq602b_04=1) or

(saq602a_05 in (4250,4260,4220,4280,4350,4360) and saq602b_05=1) or

(saq602a_06 in (4250,4260,4220,4280,4350,4360) and saq602b_06=1) or

(saq602a_07 in (4250,4260,4220,4280,4350,4360) and saq602b_07=1) or

(saq602a_08 in (4250,4260,4220,4280,4350,4360) and saq602b_08=1) or

(saq602a_09 in (4250,4260,4220,4280,4350,4360) and saq602b_09=1) or

(saq602a_10 in (4250,4260,4220,4280,4350,4360) and saq602b_10=1) or

(saq602a_11 in (4250,4260,4220,4280,4350,4360) and saq602b_11=1) or

(saq602a_12 in (4250,4260,4220,4280,4350,4360) and saq602b_12=1) or

(saq602a_13 in (4250,4260,4220,4280,4350,4360) and saq602b_13=1) or

(saq602a_14 in (4250,4260,4220,4280,4350,4360) and saq602b_14=1) or

(saq602a_15 in (4250,4260,4220,4280,4350,4360) and saq602b_15=1) or

(saq602a_16 in (4250,4260,4220,4280,4350,4360) and saq602b_16=1) or

(saq602a_17 in (4250,4260,4220,4280,4350,4360) and saq602b_17=1) or

(saq602a_18 in (4250,4260,4220,4280,4350,4360) and saq602b_18=1) or

saq6030_1=3) then IMCI_urgent_referral_day1=1; else IMCI_urgent_referral_day1=2;

* Count of IMCI urgent referral illness signs;

if (saq602a_01 in (4250,4260,4220,4280,4350,4360) and saq602b_01=1) then IMCI_urgent_day1count1=1; else IMCI_urgent_day1count1=0;

if (saq602a_02 in (4250,4260,4220,4280,4350,4360) and saq602b_02=1) then IMCI_urgent_day1count2=1; else IMCI_urgent_day1count2=0;

if (saq602a_03 in (4250,4260,4220,4280,4350,4360) and saq602b_03=1) then IMCI_urgent_day1count3=1; else IMCI_urgent_day1count3=0;

if (saq602a_04 in (4250,4260,4220,4280,4350,4360) and saq602b_04=1) then IMCI_urgent_day1count4=1; else IMCI_urgent_day1count4=0;

if (saq602a_05 in (4250,4260,4220,4280,4350,4360) and saq602b_05=1) then IMCI_urgent_day1count5=1; else IMCI_urgent_day1count5=0;

if (saq602a_06 in (4250,4260,4220,4280,4350,4360) and saq602b_06=1) then IMCI_urgent_day1count6=1; else IMCI_urgent_day1count6=0;

if (saq602a_07 in (4250,4260,4220,4280,4350,4360) and saq602b_07=1) then IMCI_urgent_day1count7=1; else IMCI_urgent_day1count7=0;

if (saq602a_08 in (4250,4260,4220,4280,4350,4360) and saq602b_08=1) then IMCI_urgent_day1count8=1; else IMCI_urgent_day1count8=0;

if (saq602a_09 in (4250,4260,4220,4280,4350,4360) and saq602b_09=1) then IMCI_urgent_day1count9=1; else IMCI_urgent_day1count9=0;

if (saq602a_10 in (4250,4260,4220,4280,4350,4360) and saq602b_10=1) then IMCI_urgent_day1count10=1; else IMCI_urgent_day1count10=0;

if (saq602a_11 in (4250,4260,4220,4280,4350,4360) and saq602b_11=1) then IMCI_urgent_day1count11=1; else IMCI_urgent_day1count11=0;

if (saq602a_12 in (4250,4260,4220,4280,4350,4360) and saq602b_12=1) then IMCI_urgent_day1count12=1; else IMCI_urgent_day1count12=0;

if (saq602a_13 in (4250,4260,4220,4280,4350,4360) and saq602b_13=1) then IMCI_urgent_day1count13=1; else IMCI_urgent_day1count13=0;

if (saq602a_14 in (4250,4260,4220,4280,4350,4360) and saq602b_14=1) then IMCI_urgent_day1count14=1; else IMCI_urgent_day1count14=0;

if (saq602a_15 in (4250,4260,4220,4280,4350,4360) and saq602b_15=1) then IMCI_urgent_day1count15=1; else IMCI_urgent_day1count15=0;

if (saq602a_16 in (4250,4260,4220,4280,4350,4360) and saq602b_16=1) then IMCI_urgent_day1count16=1; else IMCI_urgent_day1count16=0;

if (saq602a_17 in (4250,4260,4220,4280,4350,4360) and saq602b_17=1) then IMCI_urgent_day1count17=1; else IMCI_urgent_day1count17=0;

if (saq602a_18 in (4250,4260,4220,4280,4350,4360) and saq602b_18=1) then IMCI_urgent_day1count18=1; else IMCI_urgent_day1count18=0;

if saq6030_1=3 then IMCI_urgent_day1count19=1; else IMCI_urgent_day1count19=0;

IMCI_urgent_day1count=(IMCI_urgent_day1count1+IMCI_urgent_day1count2+IMCI_urgent_day1count3+IMCI_urgent_day1count4+IMCI_urgent_day1count5+

IMCI_urgent_day1count6+IMCI_urgent_day1count7+IMCI_urgent_day1count8+IMCI_urgent_day1count9+IMCI_urgent_day1count10+IMCI_urgent_day1count11+

IMCI_urgent_day1count12+IMCI_urgent_day1count13+IMCI_urgent_day1count14+IMCI_urgent_day1count15+IMCI_urgent_day1count16+

IMCI_urgent_day1count17+IMCI_urgent_day1count18+IMCI_urgent_day1count19);

/*

* Count of IMCI urgent referral illness signs (without the VASA severity scoring system illness sign);

if (saq602a_01 in (4250,4260,4220,4280,4350,4360) and saq602b_01=1) then IMCI_urgent_day1count1=1; else IMCI_urgent_day1count1=0;

if (saq602a_02 in (4250,4260,4220,4280,4350,4360) and saq602b_02=1) then IMCI_urgent_day1count2=1; else IMCI_urgent_day1count2=0;

if (saq602a_03 in (4250,4260,4220,4280,4350,4360) and saq602b_03=1) then IMCI_urgent_day1count3=1; else IMCI_urgent_day1count3=0;

if (saq602a_04 in (4250,4260,4220,4280,4350,4360) and saq602b_04=1) then IMCI_urgent_day1count4=1; else IMCI_urgent_day1count4=0;

if (saq602a_05 in (4250,4260,4220,4280,4350,4360) and saq602b_05=1) then IMCI_urgent_day1count5=1; else IMCI_urgent_day1count5=0;

if (saq602a_06 in (4250,4260,4220,4280,4350,4360) and saq602b_06=1) then IMCI_urgent_day1count6=1; else IMCI_urgent_day1count6=0;

if (saq602a_07 in (4250,4260,4220,4280,4350,4360) and saq602b_07=1) then IMCI_urgent_day1count7=1; else IMCI_urgent_day1count7=0;

if (saq602a_08 in (4250,4260,4220,4280,4350,4360) and saq602b_08=1) then IMCI_urgent_day1count8=1; else IMCI_urgent_day1count8=0;

if (saq602a_09 in (4250,4260,4220,4280,4350,4360) and saq602b_09=1) then IMCI_urgent_day1count9=1; else IMCI_urgent_day1count9=0;

if (saq602a_10 in (4250,4260,4220,4280,4350,4360) and saq602b_10=1) then IMCI_urgent_day1count10=1; else IMCI_urgent_day1count10=0;

if (saq602a_11 in (4250,4260,4220,4280,4350,4360) and saq602b_11=1) then IMCI_urgent_day1count11=1; else IMCI_urgent_day1count11=0;

if (saq602a_12 in (4250,4260,4220,4280,4350,4360) and saq602b_12=1) then IMCI_urgent_day1count12=1; else IMCI_urgent_day1count12=0;

if (saq602a_13 in (4250,4260,4220,4280,4350,4360) and saq602b_13=1) then IMCI_urgent_day1count13=1; else IMCI_urgent_day1count13=0;

if (saq602a_14 in (4250,4260,4220,4280,4350,4360) and saq602b_14=1) then IMCI_urgent_day1count14=1; else IMCI_urgent_day1count14=0;

if (saq602a_15 in (4250,4260,4220,4280,4350,4360) and saq602b_15=1) then IMCI_urgent_day1count15=1; else IMCI_urgent_day1count15=0;

if (saq602a_16 in (4250,4260,4220,4280,4350,4360) and saq602b_16=1) then IMCI_urgent_day1count16=1; else IMCI_urgent_day1count16=0;

if (saq602a_17 in (4250,4260,4220,4280,4350,4360) and saq602b_17=1) then IMCI_urgent_day1count17=1; else IMCI_urgent_day1count17=0;

if (saq602a_18 in (4250,4260,4220,4280,4350,4360) and saq602b_18=1) then IMCI_urgent_day1count18=1; else IMCI_urgent_day1count18=0;

IMCI_urgent_day1count=(IMCI_urgent_day1count1+IMCI_urgent_day1count2+IMCI_urgent_day1count3+IMCI_urgent_day1count4+IMCI_urgent_day1count5+

IMCI_urgent_day1count6+IMCI_urgent_day1count7+IMCI_urgent_day1count8+IMCI_urgent_day1count9+IMCI_urgent_day1count10+IMCI_urgent_day1count11+

IMCI_urgent_day1count12+IMCI_urgent_day1count13+IMCI_urgent_day1count14+IMCI_urgent_day1count15+IMCI_urgent_day1count16+

IMCI_urgent_day1count17+IMCI_urgent_day1count18);

*/

* 3. VASA severe illness signs (IMCI signs + other signs in the VASA, all during the entire illness);

* vacq4140=severe cough, vacq4150=vomit after coughing, vacq4230=grunting, vacq4240=wheezing, vacq4290=bulging fontanelle,

vacq4380=skin flaked off in patches, vacq4390=hair changed to reddish or yellowish color,

vacq4440=bleeding from anywhere, vacq4460=areas of skin turned black, vacq4471-78=injuries;

if (IMCI_urgent_referral=1 or vacq4140=1 or vacq4150=1 or vacq4230=1 or vacq4240 or vacq4290=1 or vacq4380=1 or vacq4390=1

or vacq4440=1 or vacq4460=1 or vacq4471=1 or vacq4472=1 or vacq4473=1 or vacq4474=1 or vacq4475=1

or vacq4476=1 or vacq4477=1 or vacq4478=1) then VASA_severe_illness=1; else VASA_severe_illness=2;

* 4. VASA severe illness signs_day1 (IMCI signs + other signs in the VASA reported on illness day-1);

* vacq4140=severe cough, vacq4150=vomit after coughing, vacq4230=grunting, vacq4240=wheezing, vacq4290=bulging fontanelle,

vacq4380=skin flaked off in patches, vacq4390=hair changed to reddish or yellowish color,

vacq4440=bleeding from anywhere, vacq4460=areas of skin turned black, vacq4471-78=injuries;

if (IMCI_urgent_referral_day1=1 or

(saq602a_01 in (4140,4150,4230,4240,4290,4380,4390,4440,4460,4471,4472,4473,4474,4475,4476,4477,4478) and saq602b_01=1) or

(saq602a_02 in (4140,4150,4230,4240,4290,4380,4390,4440,4460,4471,4472,4473,4474,4475,4476,4477,4478) and saq602b_02=1) or

(saq602a_03 in (4140,4150,4230,4240,4290,4380,4390,4440,4460,4471,4472,4473,4474,4475,4476,4477,4478) and saq602b_03=1) or

(saq602a_04 in (4140,4150,4230,4240,4290,4380,4390,4440,4460,4471,4472,4473,4474,4475,4476,4477,4478) and saq602b_04=1) or

(saq602a_05 in (4140,4150,4230,4240,4290,4380,4390,4440,4460,4471,4472,4473,4474,4475,4476,4477,4478) and saq602b_05=1) or

(saq602a_06 in (4140,4150,4230,4240,4290,4380,4390,4440,4460,4471,4472,4473,4474,4475,4476,4477,4478) and saq602b_06=1) or

(saq602a_07 in (4140,4150,4230,4240,4290,4380,4390,4440,4460,4471,4472,4473,4474,4475,4476,4477,4478) and saq602b_07=1) or

(saq602a_08 in (4140,4150,4230,4240,4290,4380,4390,4440,4460,4471,4472,4473,4474,4475,4476,4477,4478) and saq602b_08=1) or

(saq602a_09 in (4140,4150,4230,4240,4290,4380,4390,4440,4460,4471,4472,4473,4474,4475,4476,4477,4478) and saq602b_09=1) or

(saq602a_10 in (4140,4150,4230,4240,4290,4380,4390,4440,4460,4471,4472,4473,4474,4475,4476,4477,4478) and saq602b_10=1) or

(saq602a_11 in (4140,4150,4230,4240,4290,4380,4390,4440,4460,4471,4472,4473,4474,4475,4476,4477,4478) and saq602b_11=1) or

(saq602a_12 in (4140,4150,4230,4240,4290,4380,4390,4440,4460,4471,4472,4473,4474,4475,4476,4477,4478) and saq602b_12=1) or

(saq602a_13 in (4140,4150,4230,4240,4290,4380,4390,4440,4460,4471,4472,4473,4474,4475,4476,4477,4478) and saq602b_13=1) or

(saq602a_14 in (4140,4150,4230,4240,4290,4380,4390,4440,4460,4471,4472,4473,4474,4475,4476,4477,4478) and saq602b_14=1) or

(saq602a_15 in (4140,4150,4230,4240,4290,4380,4390,4440,4460,4471,4472,4473,4474,4475,4476,4477,4478) and saq602b_15=1) or

(saq602a_16 in (4140,4150,4230,4240,4290,4380,4390,4440,4460,4471,4472,4473,4474,4475,4476,4477,4478) and saq602b_16=1) or

(saq602a_17 in (4140,4150,4230,4240,4290,4380,4390,4440,4460,4471,4472,4473,4474,4475,4476,4477,4478) and saq602b_17=1) or

(saq602a_18 in (4140,4150,4230,4240,4290,4380,4390,4440,4460,4471,4472,4473,4474,4475,4476,4477,4478) and saq602b_18=1))

then VASA_severe_illness_day1=1; else VASA_severe_illness_day1=2;

* Count of IMCI day-1 urgent referral + VASA day-1 severe illness signs;

if (saq602a_01 in (4250,4260,4220,4280,4350,4360,4140,4150,4230,4240,4290,4380,4390,4440,4460,4471,4472,4473,4474,4475,4476,4477,4478) and saq602b_01=1) then VASA_severe_day1count1=1; else VASA_severe_day1count1=0;

if (saq602a_02 in (4250,4260,4220,4280,4350,4360,4140,4150,4230,4240,4290,4380,4390,4440,4460,4471,4472,4473,4474,4475,4476,4477,4478) and saq602b_02=1) then VASA_severe_day1count2=1; else VASA_severe_day1count2=0;

if (saq602a_03 in (4250,4260,4220,4280,4350,4360,4140,4150,4230,4240,4290,4380,4390,4440,4460,4471,4472,4473,4474,4475,4476,4477,4478) and saq602b_03=1) then VASA_severe_day1count3=1; else VASA_severe_day1count3=0;

if (saq602a_04 in (4250,4260,4220,4280,4350,4360,4140,4150,4230,4240,4290,4380,4390,4440,4460,4471,4472,4473,4474,4475,4476,4477,4478) and saq602b_04=1) then VASA_severe_day1count4=1; else VASA_severe_day1count4=0;

if (saq602a_05 in (4250,4260,4220,4280,4350,4360,4140,4150,4230,4240,4290,4380,4390,4440,4460,4471,4472,4473,4474,4475,4476,4477,4478) and saq602b_05=1) then VASA_severe_day1count5=1; else VASA_severe_day1count5=0;

if (saq602a_06 in (4250,4260,4220,4280,4350,4360,4140,4150,4230,4240,4290,4380,4390,4440,4460,4471,4472,4473,4474,4475,4476,4477,4478) and saq602b_06=1) then VASA_severe_day1count6=1; else VASA_severe_day1count6=0;

if (saq602a_07 in (4250,4260,4220,4280,4350,4360,4140,4150,4230,4240,4290,4380,4390,4440,4460,4471,4472,4473,4474,4475,4476,4477,4478) and saq602b_07=1) then VASA_severe_day1count7=1; else VASA_severe_day1count7=0;

if (saq602a_08 in (4250,4260,4220,4280,4350,4360,4140,4150,4230,4240,4290,4380,4390,4440,4460,4471,4472,4473,4474,4475,4476,4477,4478) and saq602b_08=1) then VASA_severe_day1count8=1; else VASA_severe_day1count8=0;

if (saq602a_09 in (4250,4260,4220,4280,4350,4360,4140,4150,4230,4240,4290,4380,4390,4440,4460,4471,4472,4473,4474,4475,4476,4477,4478) and saq602b_09=1) then VASA_severe_day1count9=1; else VASA_severe_day1count9=0;

if (saq602a_10 in (4250,4260,4220,4280,4350,4360,4140,4150,4230,4240,4290,4380,4390,4440,4460,4471,4472,4473,4474,4475,4476,4477,4478) and saq602b_10=1) then VASA_severe_day1count10=1; else VASA_severe_day1count10=0;

if (saq602a_11 in (4250,4260,4220,4280,4350,4360,4140,4150,4230,4240,4290,4380,4390,4440,4460,4471,4472,4473,4474,4475,4476,4477,4478) and saq602b_11=1) then VASA_severe_day1count11=1; else VASA_severe_day1count11=0;

if (saq602a_12 in (4250,4260,4220,4280,4350,4360,4140,4150,4230,4240,4290,4380,4390,4440,4460,4471,4472,4473,4474,4475,4476,4477,4478) and saq602b_12=1) then VASA_severe_day1count12=1; else VASA_severe_day1count12=0;

if (saq602a_13 in (4250,4260,4220,4280,4350,4360,4140,4150,4230,4240,4290,4380,4390,4440,4460,4471,4472,4473,4474,4475,4476,4477,4478) and saq602b_13=1) then VASA_severe_day1count13=1; else VASA_severe_day1count13=0;

if (saq602a_14 in (4250,4260,4220,4280,4350,4360,4140,4150,4230,4240,4290,4380,4390,4440,4460,4471,4472,4473,4474,4475,4476,4477,4478) and saq602b_14=1) then VASA_severe_day1count14=1; else VASA_severe_day1count14=0;

if (saq602a_15 in (4250,4260,4220,4280,4350,4360,4140,4150,4230,4240,4290,4380,4390,4440,4460,4471,4472,4473,4474,4475,4476,4477,4478) and saq602b_15=1) then VASA_severe_day1count11=1; else VASA_severe_day1count15=0;

if (saq602a_16 in (4250,4260,4220,4280,4350,4360,4140,4150,4230,4240,4290,4380,4390,4440,4460,4471,4472,4473,4474,4475,4476,4477,4478) and saq602b_16=1) then VASA_severe_day1count12=1; else VASA_severe_day1count16=0;

if (saq602a_17 in (4250,4260,4220,4280,4350,4360,4140,4150,4230,4240,4290,4380,4390,4440,4460,4471,4472,4473,4474,4475,4476,4477,4478) and saq602b_17=1) then VASA_severe_day1count13=1; else VASA_severe_day1count17=0;

if (saq602a_18 in (4250,4260,4220,4280,4350,4360,4140,4150,4230,4240,4290,4380,4390,4440,4460,4471,4472,4473,4474,4475,4476,4477,4478) and saq602b_18=1) then VASA_severe_day1count14=1; else VASA_severe_day1count18=0;

if saq6030_1=3 then VASA_severe_day1count19=1; else VASA_severe_day1count19=0;

VASA_severe_day1count=(VASA_severe_day1count1+VASA_severe_day1count2+VASA_severe_day1count3+VASA_severe_day1count4+VASA_severe_day1count5+

VASA_severe_day1count6+VASA_severe_day1count7+VASA_severe_day1count8+VASA_severe_day1count9+VASA_severe_day1count10+VASA_severe_day1count11+

VASA_severe_day1count12+VASA_severe_day1count13+VASA_severe_day1count14+VASA_severe_day1count15+VASA_severe_day1count16+

VASA_severe_day1count17+VASA_severe_day1count18+VASA_severe_day1count19);

/*

* Count of IMCI day-1 urgent referral + VASA day-1 severe illness signs (without the VASA severity scoring system illness sign);

if (saq602a_01 in (4250,4260,4220,4280,4350,4360,4140,4150,4230,4240,4290,4380,4390,4440,4460,4471,4472,4473,4474,4475,4476,4477,4478) and saq602b_01=1) then VASA_severe_day1count1=1; else VASA_severe_day1count1=0;

if (saq602a_02 in (4250,4260,4220,4280,4350,4360,4140,4150,4230,4240,4290,4380,4390,4440,4460,4471,4472,4473,4474,4475,4476,4477,4478) and saq602b_02=1) then VASA_severe_day1count2=1; else VASA_severe_day1count2=0;

if (saq602a_03 in (4250,4260,4220,4280,4350,4360,4140,4150,4230,4240,4290,4380,4390,4440,4460,4471,4472,4473,4474,4475,4476,4477,4478) and saq602b_03=1) then VASA_severe_day1count3=1; else VASA_severe_day1count3=0;

if (saq602a_04 in (4250,4260,4220,4280,4350,4360,4140,4150,4230,4240,4290,4380,4390,4440,4460,4471,4472,4473,4474,4475,4476,4477,4478) and saq602b_04=1) then VASA_severe_day1count4=1; else VASA_severe_day1count4=0;

if (saq602a_05 in (4250,4260,4220,4280,4350,4360,4140,4150,4230,4240,4290,4380,4390,4440,4460,4471,4472,4473,4474,4475,4476,4477,4478) and saq602b_05=1) then VASA_severe_day1count5=1; else VASA_severe_day1count5=0;

if (saq602a_06 in (4250,4260,4220,4280,4350,4360,4140,4150,4230,4240,4290,4380,4390,4440,4460,4471,4472,4473,4474,4475,4476,4477,4478) and saq602b_06=1) then VASA_severe_day1count6=1; else VASA_severe_day1count6=0;

if (saq602a_07 in (4250,4260,4220,4280,4350,4360,4140,4150,4230,4240,4290,4380,4390,4440,4460,4471,4472,4473,4474,4475,4476,4477,4478) and saq602b_07=1) then VASA_severe_day1count7=1; else VASA_severe_day1count7=0;

if (saq602a_08 in (4250,4260,4220,4280,4350,4360,4140,4150,4230,4240,4290,4380,4390,4440,4460,4471,4472,4473,4474,4475,4476,4477,4478) and saq602b_08=1) then VASA_severe_day1count8=1; else VASA_severe_day1count8=0;

if (saq602a_09 in (4250,4260,4220,4280,4350,4360,4140,4150,4230,4240,4290,4380,4390,4440,4460,4471,4472,4473,4474,4475,4476,4477,4478) and saq602b_09=1) then VASA_severe_day1count9=1; else VASA_severe_day1count9=0;

if (saq602a_10 in (4250,4260,4220,4280,4350,4360,4140,4150,4230,4240,4290,4380,4390,4440,4460,4471,4472,4473,4474,4475,4476,4477,4478) and saq602b_10=1) then VASA_severe_day1count10=1; else VASA_severe_day1count10=0;

if (saq602a_11 in (4250,4260,4220,4280,4350,4360,4140,4150,4230,4240,4290,4380,4390,4440,4460,4471,4472,4473,4474,4475,4476,4477,4478) and saq602b_11=1) then VASA_severe_day1count11=1; else VASA_severe_day1count11=0;

if (saq602a_12 in (4250,4260,4220,4280,4350,4360,4140,4150,4230,4240,4290,4380,4390,4440,4460,4471,4472,4473,4474,4475,4476,4477,4478) and saq602b_12=1) then VASA_severe_day1count12=1; else VASA_severe_day1count12=0;

if (saq602a_13 in (4250,4260,4220,4280,4350,4360,4140,4150,4230,4240,4290,4380,4390,4440,4460,4471,4472,4473,4474,4475,4476,4477,4478) and saq602b_13=1) then VASA_severe_day1count13=1; else VASA_severe_day1count13=0;

if (saq602a_14 in (4250,4260,4220,4280,4350,4360,4140,4150,4230,4240,4290,4380,4390,4440,4460,4471,4472,4473,4474,4475,4476,4477,4478) and saq602b_14=1) then VASA_severe_day1count14=1; else VASA_severe_day1count14=0;

if (saq602a_15 in (4250,4260,4220,4280,4350,4360,4140,4150,4230,4240,4290,4380,4390,4440,4460,4471,4472,4473,4474,4475,4476,4477,4478) and saq602b_15=1) then VASA_severe_day1count11=1; else VASA_severe_day1count15=0;

if (saq602a_16 in (4250,4260,4220,4280,4350,4360,4140,4150,4230,4240,4290,4380,4390,4440,4460,4471,4472,4473,4474,4475,4476,4477,4478) and saq602b_16=1) then VASA_severe_day1count12=1; else VASA_severe_day1count16=0;

if (saq602a_17 in (4250,4260,4220,4280,4350,4360,4140,4150,4230,4240,4290,4380,4390,4440,4460,4471,4472,4473,4474,4475,4476,4477,4478) and saq602b_17=1) then VASA_severe_day1count13=1; else VASA_severe_day1count17=0;

if (saq602a_18 in (4250,4260,4220,4280,4350,4360,4140,4150,4230,4240,4290,4380,4390,4440,4460,4471,4472,4473,4474,4475,4476,4477,4478) and saq602b_18=1) then VASA_severe_day1count14=1; else VASA_severe_day1count18=0;

VASA_severe_day1count=(VASA_severe_day1count1+VASA_severe_day1count2+VASA_severe_day1count3+VASA_severe_day1count4+VASA_severe_day1count5+

VASA_severe_day1count6+VASA_severe_day1count7+VASA_severe_day1count8+VASA_severe_day1count9+VASA_severe_day1count10+VASA_severe_day1count11+

VASA_severe_day1count12+VASA_severe_day1count13+VASA_severe_day1count14+VASA_severe_day1count15+VASA_severe_day1count16+

VASA_severe_day1count17+VASA_severe_day1count18);

*/

* fever in an area with malaria;

if vacq4010=1 and saq3060 in (1,2,3,9) then vacq4010=1;

else if saq3060=. then vacq4010=2;

* 5. WHO IMCI other illness (signs during the entire illness: one of the following signs: vacq4010=1: fever in an area with malaria,

diardur=diarrhea duration>=14 days, vacq4110=1: visible blood in the stool, vacq4200=1: chest indrawing, vacq4180=1: fast breathing,

vacq4410=1: lack of blood or pallor):;

if (vacq4010=1 or diardur>=14 or vacq4110=1 or vacq4200=1 or vacq4180=1 or vacq4410=1)

then IMCI_other_illness=1; else IMCI_other_illness=2;

* 6. WHO IMCI other illness_day1 (signs on illness day 1): Cannot include persistent diarrhea without dehydration on illness day-1.

Even if respondents mean that the illness "started" once diarrhea had lasted 14 days, there is no way to indicate this or that

there was no dehydation with the VASA variables, nor is it possible to code diarrhea with some dehydration since there are no

dehydration variables in the VASA questionnaire;

if ((saq602a_01 in (4010,4110,4200,4180,4410) and saq602b_01=1) or

(saq602a_02 in (4010,4110,4200,4180,4410) and saq602b_02=1) or

(saq602a_03 in (4010,4110,4200,4180,4410) and saq602b_03=1) or

(saq602a_04 in (4010,4110,4200,4180,4410) and saq602b_04=1) or

(saq602a_05 in (4010,4110,4200,4180,4410) and saq602b_05=1) or

(saq602a_06 in (4010,4110,4200,4180,4410) and saq602b_06=1) or

(saq602a_07 in (4010,4110,4200,4180,4410) and saq602b_07=1) or

(saq602a_08 in (4010,4110,4200,4180,4410) and saq602b_08=1) or

(saq602a_09 in (4010,4110,4200,4180,4410) and saq602b_09=1) or

(saq602a_10 in (4010,4110,4200,4180,4410) and saq602b_10=1) or

(saq602a_11 in (4010,4110,4200,4180,4410) and saq602b_11=1) or

(saq602a_12 in (4010,4110,4200,4180,4410) and saq602b_12=1) or

(saq602a_13 in (4010,4110,4200,4180,4410) and saq602b_13=1) or

(saq602a_14 in (4010,4110,4200,4180,4410) and saq602b_14=1) or

(saq602a_15 in (4010,4110,4200,4180,4410) and saq602b_15=1) or

(saq602a_16 in (4010,4110,4200,4180,4410) and saq602b_16=1) or

(saq602a_17 in (4010,4110,4200,4180,4410) and saq602b_17=1) or

(saq602a_18 in (4010,4110,4200,4180,4410) and saq602b_18=1)) or saq6030_1=2

then IMCI_other_illness_day1=1; else IMCI_other_illness_day1=2;

* 7. VASA other illness (IMCI signs + other signs in the VASA at any time during the illness);

* vacq4070>=3=3+ stools on the worst day, vacq4130>=7=cough for 7+ days, vacq4160=difficult breathing,

vacq4340=blisters with clear fluid, vacq4420=swelling in the armpits, vacq4430=whitish rash in the mouth/tongue;

if (IMCI_other_illness=1 or (vacq4070>=3 and vacq4070<99) or (vacq4130>=7 and vacq4130<99) or vacq4160=1 or

vacq4340=1 or vacq4420=1 or vacq4430=1) then VASA_other_illness=1; else VASA_other_illness=2;

* cough 7+ days;

if saq602a_01=4120 and vacq4130>=7 and vacq4130<99 then saq602a_01=4120;

else if saq602a_01=4120 and vacq4130 in (1,2,3,4,5,6,99) then saq602a_01=.;

if saq602a_02=4120 and vacq4130>=7 and vacq4130<99 then saq602a_02=4120;

else if saq602a_02=4120 and vacq4130 in (1,2,3,4,5,6,99) then saq602a_02=.;

if saq602a_03=4120 and vacq4130>=7 and vacq4130<99 then saq602a_03=4120;

else if saq602a_03=4120 and vacq4130 in (1,2,3,4,5,6,99) then saq602a_03=.;

if saq602a_04=4120 and vacq4130>=7 and vacq4130<99 then saq602a_04=4120;

else if saq602a_04=4120 and vacq4130 in (1,2,3,4,5,6,99) then saq602a_04=.;

if saq602a_05=4120 and vacq4130>=7 and vacq4130<99 then saq602a_05=4120;

else if saq602a_05=4120 and vacq4130 in (1,2,3,4,5,6,99) then saq602a_05=.;

if saq602a_06=4120 and vacq4130>=7 and vacq4130<99 then saq602a_06=4120;

else if saq602a_06=4120 and vacq4130 in (1,2,3,4,5,6,99) then saq602a_06=.;

if saq602a_07=4120 and vacq4130>=7 and vacq4130<99 then saq602a_07=4120;

else if saq602a_07=4120 and vacq4130 in (1,2,3,4,5,6,99) then saq602a_07=.;

if saq602a_08=4120 and vacq4130>=7 and vacq4130<99 then saq602a_08=4120;

else if saq602a_08=4120 and vacq4130 in (1,2,3,4,5,6,99) then saq602a_08=.;

if saq602a_09=4120 and vacq4130>=7 and vacq4130<99 then saq602a_09=4120;

else if saq602a_09=4120 and vacq4130 in (1,2,3,4,5,6,99) then saq602a_09=.;

if saq602a_10=4120 and vacq4130>=7 and vacq4130<99 then saq602a_10=4120;

else if saq602a_10=4120 and vacq4130 in (1,2,3,4,5,6,99) then saq602a_10=.;

if saq602a_11=4120 and vacq4130>=7 and vacq4130<99 then saq602a_11=4120;

else if saq602a_11=4120 and vacq4130 in (1,2,3,4,5,6,99) then saq602a_11=.;

if saq602a_12=4120 and vacq4130>=7 and vacq4130<99 then saq602a_12=4120;

else if saq602a_12=4120 and vacq4130 in (1,2,3,4,5,6,99) then saq602a_12=.;

if saq602a_13=4120 and vacq4130>=7 and vacq4130<99 then saq602a_13=4120;

else if saq602a_13=4120 and vacq4130 in (1,2,3,4,5,6,99) then saq602a_13=.;

if saq602a_14=4120 and vacq4130>=7 and vacq4130<99 then saq602a_14=4120;

else if saq602a_14=4120 and vacq4130 in (1,2,3,4,5,6,99) then saq602a_14=.;

if saq602a_15=4120 and vacq4130>=7 and vacq4130<99 then saq602a_15=4120;

else if saq602a_15=4120 and vacq4130 in (1,2,3,4,5,6,99) then saq602a_15=.;

if saq602a_16=4120 and vacq4130>=7 and vacq4130<99 then saq602a_16=4120;

else if saq602a_16=4120 and vacq4130 in (1,2,3,4,5,6,99) then saq602a_16=.;

if saq602a_17=4120 and vacq4130>=7 and vacq4130<99 then saq602a_17=4120;

else if saq602a_17=4120 and vacq4130 in (1,2,3,4,5,6,99) then saq602a_17=.;

if saq602a_18=4120 and vacq4130>=7 and vacq4130<99 then saq602a_18=4120;

else if saq602a_18=4120 and vacq4130 in (1,2,3,4,5,6,99) then saq602a_18=.;

* 3+ stools on the worst day;

if saq602a_01=4060 and vacq4070>=3 and vacq4070<99 then saq602a_01=4060;

else if saq602a_01=4060 and vacq4070 in (1,2,99) then saq602a_01=.;

if saq602a_02=4060 and vacq4070>=3 and vacq4070<99 then saq602a_02=4060;

else if saq602a_02=4060 and vacq4070 in (1,2,99) then saq602a_02=.;

if saq602a_03=4060 and vacq4070>=3 and vacq4070<99 then saq602a_03=4060;

else if saq602a_03=4060 and vacq4070 in (1,2,99) then saq602a_03=.;

if saq602a_04=4060 and vacq4070>=3 and vacq4070<99 then saq602a_04=4060;

else if saq602a_04=4060 and vacq4070 in (1,2,99) then saq602a_04=.;

if saq602a_05=4060 and vacq4070>=3 and vacq4070<99 then saq602a_05=4060;

else if saq602a_05=4060 and vacq4070 in (1,2,99) then saq602a_05=.;

if saq602a_06=4060 and vacq4070>=3 and vacq4070<99 then saq602a_06=4060;

else if saq602a_06=4060 and vacq4070 in (1,2,99) then saq602a_06=.;

if saq602a_07=4060 and vacq4070>=3 and vacq4070<99 then saq602a_07=4060;

else if saq602a_07=4060 and vacq4070 in (1,2,99) then saq602a_07=.;

if saq602a_08=4060 and vacq4070>=3 and vacq4070<99 then saq602a_08=4060;

else if saq602a_08=4060 and vacq4070 in (1,2,99) then saq602a_08=.;

if saq602a_09=4060 and vacq4070>=3 and vacq4070<99 then saq602a_09=4060;

else if saq602a_09=4060 and vacq4070 in (1,2,99) then saq602a_09=.;

if saq602a_10=4060 and vacq4070>=3 and vacq4070<99 then saq602a_10=4060;

else if saq602a_10=4060 and vacq4070 in (1,2,99) then saq602a_10=.;

if saq602a_11=4060 and vacq4070>=3 and vacq4070<99 then saq602a_11=4060;

else if saq602a_11=4060 and vacq4070 in (1,2,99) then saq602a_11=.;

if saq602a_12=4060 and vacq4070>=3 and vacq4070<99 then saq602a_12=4060;

else if saq602a_12=4060 and vacq4070 in (1,2,99) then saq602a_12=.;

if saq602a_13=4060 and vacq4070>=3 and vacq4070<99 then saq602a_13=4060;

else if saq602a_13=4060 and vacq4070 in (1,2,99) then saq602a_13=.;

if saq602a_14=4060 and vacq4070>=3 and vacq4070<99 then saq602a_14=4060;

else if saq602a_14=4060 and vacq4070 in (1,2,99) then saq602a_14=.;

if saq602a_15=4060 and vacq4070>=3 and vacq4070<99 then saq602a_15=4060;

else if saq602a_15=4060 and vacq4070 in (1,2,99) then saq602a_15=.;

if saq602a_16=4060 and vacq4070>=3 and vacq4070<99 then saq602a_16=4060;

else if saq602a_16=4060 and vacq4070 in (1,2,99) then saq602a_16=.;

if saq602a_17=4060 and vacq4070>=3 and vacq4070<99 then saq602a_17=4060;

else if saq602a_17=4060 and vacq4070 in (1,2,99) then saq602a_17=.;

if saq602a_18=4060 and vacq4070>=3 and vacq4070<99 then saq602a_18=4060;

else if saq602a_18=4060 and vacq4070 in (1,2,99) then saq602a_18=.;

* 8a. VASA other illness_day1 (IMCI signs + other signs in the VASA on illness day-1);

* 4060=vacq4070>=3=3+ stools on the worst day, 4120=vacq4130>=7=cough for 7+ days, vacq4160=difficult breathing,

vacq4340=blisters with clear fluid, vacq4420=swelling in the armpits, vacq4430=whitish rash in the mouth/tongue;

if (IMCI_other_illness_day1=1 or

(saq602a_01 in (4060,4120,4160,4340,4420,4430) and saq602b_01=1) or

(saq602a_02 in (4060,4120,4160,4340,4420,4430) and saq602b_02=1) or

(saq602a_03 in (4060,4120,4160,4340,4420,4430) and saq602b_03=1) or

(saq602a_04 in (4060,4120,4160,4340,4420,4430) and saq602b_04=1) or

(saq602a_05 in (4060,4120,4160,4340,4420,4430) and saq602b_05=1) or

(saq602a_06 in (4060,4120,4160,4340,4420,4430) and saq602b_06=1) or

(saq602a_07 in (4060,4120,4160,4340,4420,4430) and saq602b_07=1) or

(saq602a_08 in (4060,4120,4160,4340,4420,4430) and saq602b_08=1) or

(saq602a_09 in (4060,4120,4160,4340,4420,4430) and saq602b_09=1) or

(saq602a_10 in (4060,4120,4160,4340,4420,4430) and saq602b_10=1) or

(saq602a_11 in (4060,4120,4160,4340,4420,4430) and saq602b_11=1) or

(saq602a_12 in (4060,4120,4160,4340,4420,4430) and saq602b_12=1) or

(saq602a_13 in (4060,4120,4160,4340,4420,4430) and saq602b_13=1) or

(saq602a_14 in (4060,4120,4160,4340,4420,4430) and saq602b_14=1) or

(saq602a_15 in (4060,4120,4160,4340,4420,4430) and saq602b_15=1) or

(saq602a_16 in (4060,4120,4160,4340,4420,4430) and saq602b_16=1) or

(saq602a_17 in (4060,4120,4160,4340,4420,4430) and saq602b_17=1) or

(saq602a_18 in (4060,4120,4160,4340,4420,4430) and saq602b_18=1))

then VASA_other_illness_day1=1; else VASA_other_illness_day1=2;

* 8b. VASA other illness_day1 without cough>=7 days (IMCI signs + other signs in the VASA on illness day-1);

* 4060=vacq4070>=3=3+ stools on the worst day, vacq4160=difficult breathing,

vacq4340=blisters with clear fluid, vacq4420=swelling in the armpits, vacq4430=whitish rash in the mouth/tongue;

if (IMCI_other_illness_day1=1 or

(saq602a_01 in (4060,4160,4340,4420,4430) and saq602b_01=1) or

(saq602a_02 in (4060,4160,4340,4420,4430) and saq602b_02=1) or

(saq602a_03 in (4060,4160,4340,4420,4430) and saq602b_03=1) or

(saq602a_04 in (4060,4160,4340,4420,4430) and saq602b_04=1) or

(saq602a_05 in (4060,4160,4340,4420,4430) and saq602b_05=1) or

(saq602a_06 in (4060,4160,4340,4420,4430) and saq602b_06=1) or

(saq602a_07 in (4060,4160,4340,4420,4430) and saq602b_07=1) or

(saq602a_08 in (4060,4160,4340,4420,4430) and saq602b_08=1) or

(saq602a_09 in (4060,4160,4340,4420,4430) and saq602b_09=1) or

(saq602a_10 in (4060,4160,4340,4420,4430) and saq602b_10=1) or

(saq602a_11 in (4060,4160,4340,4420,4430) and saq602b_11=1) or

(saq602a_12 in (4060,4160,4340,4420,4430) and saq602b_12=1) or

(saq602a_13 in (4060,4160,4340,4420,4430) and saq602b_13=1) or

(saq602a_14 in (4060,4160,4340,4420,4430) and saq602b_14=1) or

(saq602a_15 in (4060,4160,4340,4420,4430) and saq602b_15=1) or

(saq602a_16 in (4060,4160,4340,4420,4430) and saq602b_16=1) or

(saq602a_17 in (4060,4160,4340,4420,4430) and saq602b_17=1) or

(saq602a_18 in (4060,4160,4340,4420,4430) and saq602b_18=1))

then VASA_other_illness_day1_2=1; else VASA_other_illness_day1_2=2;

* 3-level IMCI day-1 illness category (IMCI signs in the VASA on illness day-1);

if IMCI_urgent_referral_day1=1 then IMCI_3levels_day1=3;

else if IMCI_other_illness_day1=1 and IMCI_urgent_referral_day1=2 then IMCI_3levels_day1=2;

else if IMCI_urgent_referral_day1=2 and IMCI_other_illness_day1=2 then IMCI_3levels_day1=1;

if IMCI_other_illness_day1=. and IMCI_urgent_referral_day1=. then IMCI_3levels_day1=.;

* 3-level VASA day-1 illness category (IMCI signs + other signs in the VASA on illness day-1);

if VASA_severe_illness_day1=1 then VASA_3levels_day1=3;

else if VASA_other_illness_day1=1 and VASA_severe_illness_day1=2 then VASA_3levels_day1=2;

else if VASA_severe_illness_day1=2 and VASA_other_illness_day1=2 then VASA_3levels_day1=1;

if VASA_other_illness_day1=. and VASA_severe_illness_day1=. then VASA_3levels_day1=.;

* VASA_3levels_day1 category for logistic;

if VASA_3levels_day1=1 then VASA_3levels_day1cat=0;

if VASA_3levels_day1=2 then VASA_3levels_day1cat=1;

if VASA_3levels_day1=3 then VASA_3levels_day1cat=2;

if VASA_3levels_day1=. then VASA_3levels_day1cat=.;

* -------------------------------------------------------------------------------------------------;

* --------------------------------------Illness severity-------------------------------------------;

*--------------------------------------------------------------------------------------------------;
