# Supplementary material for: Development of an effective illness severity measure and assessment of the impact of perceived illness severity on formal careseeking for fatal illnesses of neonates and infants in six sub-Saharan Africa countries and Pakistan
Source: PLOS Glob Public Health. 2026 May 22;6(5):e0006455. doi: 10.1371/journal.pgph.0006455 (PMC13197068; doi:10.1371/journal.pgph.0006455)
Supplement: S1 Checklist — (DOCX) [file pgph.0006455.s012.docx]

Inclusivity in global research

PLOS’ policy on inclusivity in global research aims to improve transparency in the reporting of research performed outside of researchers’ own country or community and ensures that PLOS publications reporting global research adhere to high standards for research ethics and authorship. Authors of relevant research articles may be asked to complete the questionnaire below, which outlines ethical, cultural, and scientific considerations specific to inclusivity in global research. This questionnaire may be requested when researchers have travelled to a different country to conduct research, if research uses samples collected in another country, research with Indigenous populations or their lands, or if research is on cultural artefacts. Researchers travelling to another country solely to use laboratory equipment will not normally be required to complete the questionnaire. However, the questionnaire can be requested at the journal’s discretion for any submission – if you have been requested to complete this questionnaire by the PLOS journal you submitted to, please do so.

Please complete the questionnaire below and include this as a Supporting Information file with your manuscript. Note that if your paper is accepted for publication, this checklist will be published with your article in the supporting information files. Please ensure that you reference the checklist in the main body of your manuscript. We suggest adding a subsection ‘Inclusivity in global research’ to your Methods section and adding the following sentence: “Additional information regarding the ethical, cultural, and scientific considerations specific to inclusivity in global research is included in the Supporting Information (SX Checklist)”

The questions have been designed to be applicable to a wide range of study types, and there are subsections for both human subjects research and non-human subjects research. If any of the questions are not relevant to your research please mark them as “N/A” as appropriate.

**Ethical considerations, permits and authorship**

*This section is applicable to all research types.*

Provide details as to who granted permissions and/or consent for the study to take place in the Methods section of your manuscript. This should include the names of **all** ethics boards, governmental organizations, community leaders or other bodies that provided approval for the study. If individuals provided approval refer to these people by their role or title but do not list their name(s).

Reported on page number: 10 of the revised manuscript

If there were any deviations from the study protocol after approval was obtained please provide details of these changes in the Methods section of your manuscript.
Did this study involve local collaborators that are residents of the country where the research was conducted or members of the community studied? If you do not have any authors from said communities, please provide an explanation for this below.

Reported on page number: Not applicable (no changes were made to the seven VASA study protocols whose data were utilized for the current paper’s analyses)

We worked with a local collaborator from the national statistics office, national research institute, or a local university in each of the seven study countries. The local collaborator from each country is a co-author of the manuscript. All authors meet PLOS criteria for authorship.

Everyone listed as an author should meet PLOS’ criteria for authorship and all individuals who meet these criteria should be included in the author byline, rather than the acknowledgements. For further information please see the journal’s Authorship Policy.

**Human subjects research (e.g. health research, medical research, cross-cultural psychology)** Did you obtain written informed consent from a representative of the local community or region before the research took place? How did you establish who speaks for the community? Details of written informed consent obtained from study participants should be reported separately in the Methods section of your manuscript.

The current study conducted a secondary analysis of data obtained from a prior verbal and social autopsy (VASA) study conducted in each of seven countries. Two of the VASA studies were conducted at multiple district level and the other five were all at national level, with interviews conducted of families that had a child death identified by a district- or nation-wide survey such as a Demographic and Health Survey that included mortality assessment. Following is a description provided by the local collaborator in each of the seven VASA study countries of the process followed to inform and receive consent for the study from a community representative in their country:

Cameroon: The study was conducted in collaboration with the INS (National Institute of Statistics), which is responsible for producing official statistics. All data collection operations are conducted in strict compliance with statutory missions and the quality standards outlined in the statistical law. Field visits are preceded by preparatory activities (preparation of technical documents, training of agents and field supervisors), as well as informing and raising awareness among administrative and traditional authorities according to their respective ranks and positions. Closed contacts are carried out by supervisors and team leaders.

Nigeria: In Nigeria at the first instance community approval is sought from the gatekeepers for the team entry and then households consent are obtained during the DHS for a revisit of the VASA team.

Malawi: Malawi has an established administrative and traditional hierarchy. For the administrative roles the district commissioner and local police serve as the community representatives. In the traditional structure the Traditional Authority and the chiefs and sub-chiefs that serve under the Traditional Authorities are the established community representative. There are no additional steps required to establish a person to speak for a community as this is established in both the administrative and traditional structures. NSO (National Statistics Office) conducted official introductions on the study that was being conducted with both groups before the study commenced.

Niger: Before the start of any research, the INS (National Institute of Statistics), as the national body responsible for this mandate, sends a letter to the Ministry of the Interior to inform all community leaders about the research process and request their cooperation. At the community level, the research teams introduce themselves to the traditional chief, presenting a copy of the letter sent to the Ministry of the Interior and their identification badges, in order to explain the purpose of their visit and identify the appropriate respondents.

Tanzania: We obtained a verbal consent from the regions and local leaders who received instructions from the ministry of local government to allow researchers to go to the households.

Mozambique: The process of selecting the areas for the study followed all political and administrative procedures, and all required authorizations were obtained. Whenever VASA data collectors go to the field, they report to the community authorities and present a credential that must be stamped before starting the interviews.

Pakistan: No written consent was taken from the community representatives. However, before conducting any activities in a locality, community elders and influencers were engaged and briefed about the project.

How did members of the local community provide input on the aims of the research investigation, its methodology, and its anticipated outcome(s)?

The local collaborator in the national statistics office, national research institute or local university provided input on the aims of the research investigation, its methodology and its anticipated outcomes.

When engaging with the local community, how did you ensure that the informed consent documents and other materials could be understood by local stakeholders?

All informed consent documents and questionnaires utilized by the VASA studies (that provided the secondary data utilized by the current study) were translated by local collaborators/investigators to one or more local language(s) (e.g. four in Nigeria and six in Cameroon) as required to ensure understanding by all respondents. The translated questionnaires were back-translated to English and compared to the original English versions to ensure the accuracy of the translations. In addition, in each country questionnaires were reviewed by a local anthropologist or other social scientist to ensure that all included illness signs and symptoms were expressed in local terminology understandable by the local lay population. Where needed, multiple such terms were included in the questionnaire to provide a choice during the interview in case the first option was not well-understood by the respondent. In some countries, where the anthropologist or social scientist found this to be necessary, they conducted focus group discussions with mothers of young children to ensure that the translations were understandable and to make any needed revisions. As part of the informed consent process and the VASA interview respondents were encouraged to ask questions if they required clarification, respectively, of any part of the consent document before giving their consent or any questionnaire item before responding. Furthermore, the VASA interview in each country was conducted as a CAPI (Computer Aided Personal Interview) that detected inconsistencies in responses to individual items and allowed the data collector to return to inconsistently-responded items to discuss with the respondent and resolve any inconsistencies before recording the final response to each question.

Will the findings of the research be made available in an understandable format to stakeholders in the community where the study was conducted (e.g. via a presentation, summary report, copies of publications, etc.)? Please provide details of how this will be achieved.

A data analysis meeting was held in several of the VASA study (that provided the secondary data utilized by the current study) countries together with the Johns Hopkins researchers and colleagues from the national statistics office and ministry of health. The local collaborators produced an in-depth report of the study for the local public health community to document the study aims, methods, materials, results, conclusions and recommendations for strengthening of current interventions and potential development of new interventions to decrease neonatal and child mortality based on the study findings. A dissemination meeting was held with national and international stakeholders in each of the African VASA study countries to share the study results and discuss implications for the strengthening of ongoing interventions and possible need for new interventions. Findings of the VASA study conducted in each country were published in peer-reviewed international public health journals. The findings of the current study will be disseminated in each country as determined by the the local collaborating institution.

**Non-human subjects research using specimens/ animals collected as part of the study, or those housed in archival collections. Examples include archaeology, paleontology, botany and zoology.**

Did the permission you obtained from a local authority to perform the study include an agreement on access to outputs and benefit sharing? This may include procedures to enable fair distribution of the benefits and resources arising from the research performed. Please include any details of Prior Informed Consent and Benefit Sharing Agreements obtained. These may be required by field-specific regulations, for example the Convention on Biological Diversity (CBD) and the associated Nagoya Protocol.

Not applicable – the study did not include non-human subjects.

If the material used in your study was imported, please A) provide the year it was imported and B) indicate whether permits were obtained to import/export the materials used, C) provide details of any permits obtained. If this information is not available, please indicate this.

Not applicable

If you used archival specimens, please state how the material used in your study was acquired by the institute it is held in and provide details of any permits obtained for the original excavations/ sample collection. If this information is not available, please indicate this.

Not applicable

How was the potential cultural significance of the materials collected in your study to local communities considered in your research design? Were Indigenous peoples and/or local researchers and institutions involved with archaeological excavations / collection of specimens? If so, please provide a description of their involvement.

Not applicable

If your manuscript includes photographs of human remains please indicate whether authors obtained permission from descendants or affiliated cultural communities to do so.

Not applicable
