# Supplementary material for: Development of an effective illness severity measure and assessment of the impact of perceived illness severity on formal careseeking for fatal illnesses of neonates and infants in six sub-Saharan Africa countries and Pakistan
Source: PLOS Glob Public Health. 2026 May 22;6(5):e0006455. doi: 10.1371/journal.pgph.0006455 (PMC13197068; doi:10.1371/journal.pgph.0006455)
Supplement: Attachment — Submitted filename: Response to reviewers.docx [file pgph.0006455.s013.docx]

**Journal requirements:**

1. Please include a complete copy of PLOS’ questionnaire on inclusivity in global research in your revised manuscript.

**Response**: The completed questionnaire has been uploaded to editorialmanager.com as part of the revised manuscript submission.

2. Please upload all main figures as separate Figure files in .tif or .eps format only.

**Response**: The manuscript’s two figure files are being uploaded as separate Figure files in .tif format and meet all the NAAS site requirements.

Reviewers' comments:

Reviewer's Responses to Questions

**Comments to the Author**

1. Does this manuscript meet PLOS Global Public Health’s [publication criteria](https://nam02.safelinks.protection.outlook.com/?url=https%3A%2F%2Ftrack.editorialmanager.com%2FCL0%2Fhttps%3A%252F%252Fjournals.plos.org%252Fglobalpublichealth%252Fs%252Fcriteria-for-publication%2F1%2F010f01992cdf9432-b3978883-2cfb-478d-b772-6e31d89a7e09-000000%2Febazr3q57xE13rpy1pIIAWk1obERUFTKhFVOvArFjeA%3D227&data=05%7C02%7Chkalter1%40jhu.edu%7Cd2b3a116125944158b1608ddef5ed5e1%7C9fa4f438b1e6473b803f86f8aedf0dec%7C0%7C0%7C638929912782040265%7CUnknown%7CTWFpbGZsb3d8eyJFbXB0eU1hcGkiOnRydWUsIlYiOiIwLjAuMDAwMCIsIlAiOiJXaW4zMiIsIkFOIjoiTWFpbCIsIldUIjoyfQ%3D%3D%7C0%7C%7C%7C&sdata=Ssv%2Bgi%2F5mY3YdlbSIhesNQB7r%2FEEasi85j6kp8swkiU%3D&reserved=0)? Is the manuscript technically sound, and do the data support the conclusions? The manuscript must describe methodologically and ethically rigorous research with conclusions that are appropriately drawn based on the data presented.

**Reviewer #1: Yes**

2. Has the statistical analysis been performed appropriately and rigorously?

**Reviewer #1: Yes**

3. Have the authors made all data underlying the findings in their manuscript fully available (please refer to the Data Availability Statement at the start of the manuscript PDF file)?

The [PLOS Data policy](https://nam02.safelinks.protection.outlook.com/?url=https%3A%2F%2Ftrack.editorialmanager.com%2FCL0%2Fhttps%3A%252F%252Fjournals.plos.org%252Fglobalpublichealth%252Fs%252Fdata-availability%2F1%2F010f01992cdf9432-b3978883-2cfb-478d-b772-6e31d89a7e09-000000%2FzSgCu4IpMcCoWo9KS0BLs_BfMi0yVdagg34n52gb04I%3D227&data=05%7C02%7Chkalter1%40jhu.edu%7Cd2b3a116125944158b1608ddef5ed5e1%7C9fa4f438b1e6473b803f86f8aedf0dec%7C0%7C0%7C638929912782091918%7CUnknown%7CTWFpbGZsb3d8eyJFbXB0eU1hcGkiOnRydWUsIlYiOiIwLjAuMDAwMCIsIlAiOiJXaW4zMiIsIkFOIjoiTWFpbCIsIldUIjoyfQ%3D%3D%7C0%7C%7C%7C&sdata=VI6CS22kY48y%2BU16aB1BopflNnOY7dgrgcAk97H%2FHDE%3D&reserved=0) requires authors to make all data underlying the findings described in their manuscript fully available without restriction, with rare exception. The data should be provided as part of the manuscript or its supporting information, or deposited to a public repository. For example, in addition to summary statistics, the data points behind means, medians and variance measures should be available. If there are restrictions on publicly sharing data—e.g. participant privacy or use of data from a third party—those must be specified.

**Reviewer #1 comment: No**

**Response**: With this submission, all data underlying the findings described in the revised manuscript will be uploaded as supplemental files to editorialmanager.com. I understand that PLOS Global Public Health will upload data files that can be made publicly accessible to figshare if the manuscript is accepted for publication. Five of the seven countries whose data were utilized for the paper’s analyses allow their data to be made open access, while two of the countries require that data requestors contact a country representative to request their data. Details, including how these data may be requested, are provided in the Data Accessibility Statement.

4. Is the manuscript presented in an intelligible fashion and written in standard English?

PLOS Global Public Health does not copyedit accepted manuscripts, so the language in submitted articles must be clear, correct, and unambiguous. Any typographical or grammatical errors should be corrected at revision, so please note any specific errors here.

**Reviewer #1: Yes**

5. Review Comments to the Author

Please use the space provided to explain your answers to the questions above. You may also include additional comments for the author, including concerns about dual publication, research ethics, or publication ethics. (Please upload your review as an attachment if it exceeds 20,000 characters)

Reviewer #1: The authors report what appears to be a retrospective cross-sectional study to develop scoring systems to identify mild, moderate, and severe illness among young children who died using data from VASAs conducted in several sub-Saharan African and Asian countries. The strengths of this study are the multi-country nature, the extensive analyses, and the addressing of an important question (i.e., how can we best support families’ decision to seek care at an appropriate time in the course of a child’s illness).

Despite the article’s strengths, there are several weaknesses that I think should be addressed. Some of these major weaknesses include the

**Reviewer comment 1**: need to clearly define the outcomes of mild, moderate, and severe illness. As written, it seems somewhat circular as caregivers seem to report these based on their understanding.

**Response** (*italics* added here and elsewhere for emphasis and/or to indicate manuscript revisions): First of all, it should be noted that the entire analysis examines the association of illness severity (mild, moderate, severe) *at the onset of a fatal illness* (which is clearly stated throughout the manuscript and is further addressed below [response to reviewer comment 4]) with formal careseeking *at any time during the illness*. Clarification of this point is added at several places in the manuscript, for example:

- Abstract: “We then examined the relationship of caregivers’ perception of their child’s condition at onset of a fatal illness to the child’s age and seeking formal health care *at any time during the illness*.”
- Tables 3, 4 (now 4 and 5) and Annex Table 7: Footnotes have been added for the careseeking column headings to clarify that these mean careseeking during the entire illness.

Regarding the final outcome (survival vs death), several locations in the manuscript make clear that all the children died from the examined illness, including:

- Paper’s title: “…formal careseeking for *fatal illnesses* of neonates and infants…”
- First sentence of the last paragraph of the paper’s Introduction: “The current paper develops sign-based methods of identifying mild, moderate, and severe conditions of the most vulnerable children, including neonates and 1-11-month-old infants, *at the onset of a fatal illness*.”
- First sentence of the third paragraph of the Methods section: “We developed three methods using VA-identified signs and symptoms to classify illness severity *during various stages of neonates’ and infants’ fatal illnesses*;…”
- And many other locations throughout the manuscript.

Regarding the association of illness severity level with careseeking, this is made clear at several locations in the manuscript, including additions to the Abstract and revised language in response to reviewer comment 10:

- Abstract (see abstract for newly added statistics): “…formal careseeking was almost uniformly decreased for severely ill neonates compared to those who were mildly or moderately ill...” “Multivariate analysis revealed that older age…and moderate illness…were positively associated with careseeking, while severe illness no longer played a significant role.” “Moderately ill infants… …were somewhat older…and, just as for neonates, formal careseeking was driven primarily by older age...”
  - Here we see that in the multivariate analysis careseeking depended more on age than on the degree of illness severity.
- Page 17: “Table 3 (now 4) shows a significant negative association between illness severity and formal careseeking for neonates with a fatal illness in two of the six African countries, and an apparent negative or near-negative association in three other countries that is punctuated by greater careseeking for moderate than either mild or severe illness (figure 1a).”
  - This makes clear that in five of the six African countries there was a negative univariate association of the illness severity level with formal careseeking, i.e., careseeking was greater for mild than severe illness, but that in three of these countries careseeking was greatest for moderate illness.
- Page 17 : “There was also a negative association between age and illness severity in these five countries. In Pakistan, the opposite relationship was found between formal careseeking and illness severity—there was a significant positive association; while, just as in the African countries, illness severity increased with decreasing age at death (table 3 [now 4] and figure 2a).”
  - These findings make clear that in Pakistan formal careseeking was greater for severe illness than for moderate illness and was greater for moderate than for mild illness; and presage the multivariate analyses findings that, in general, age was a stronger predictor of careseeking than illness severity and explain why careseeking was often least for the most severely ill (youngest) neonates.
- Page 19: “The association of illness severity with careseeking was less definitive for 1-11-month-olds than for neonates. Only in Tanzania was there a (nearly) significant negative association between illness severity and formal careseeking; while in Nigeria, like the finding for neonates, careseeking was greater for moderately ill than either mildly or severely ill children. This was also true in Cameroon and Niger but not significantly so (tables 3 [now 4] and figure 1b).”
  - The newly added sentence (“The association of illness severity…”) at the start of this paragraph emphasizes that the association of illness severity with careseeking was less definitive for 1-11-month-olds than for neonates.
- Table 4 (now 5) and Annex Table 7: A footnote has been added to the illness severity row for each country indicating that this means the severity level at onset of the illness (neonates) or on illness day-1 (1-11-month-olds).

Regarding the reviewer’s concern that the outcomes of mild, moderate and severe illness seem somewhat circular as caregivers seem to report these based on their understanding:

- Nowhere does the paper state that caregivers were asked whether their child had a mild, moderate or severe illness because they never were asked this. Rather, the paper clearly describes that caregivers were asked about several illness signs and symptoms and that the reported signs and symptoms were utilized to form the 2-sign and multiple-sign scores of illness severity, as detailed by Panels 1 and 2. This methodology should obviate the reviewer’s concern of circularity since caregivers did not know how their responses were to be used to develop the illness severity scoring system.

**Reviewer comment 2**: There needs to be a clear description of when these interviews were conducted relative to the child’s death and the events they reported about. Recall bias could potentially be huge for such details on specific days of illness if these interviews were conducted as a census planned for several years apart, which obviously wouldn’t coincide with the death of a child.

**Response**: The following has been added to the end of the first paragraph of the Materials and Methods section: “The years of the deaths and conduct of the VASA interviews in each country were: Cameroon 2006-10: 2012, Nigeria 2008-13: 2014, Malawi 2008-12: 2013, Niger 2007-10: 2012, Tanzania 2010-15: 2017, Mozambique 2018-20: 2018-20, Pakistan 2015-19: 2018-19.” The Limitations sub-section of the Discussion already included the following sentence: “Being interview-based, the findings are also subject to the possible influence of faulty recall and biased reporting of events.” This has been modified to read: “Being interview-based, for the most part conducted from one to five years after the death, the findings are also subject to the possible influence of faulty recall and biased reporting of events.”

**Reviewer comment 3**: better description of how the 2-sign scoring system was developed needs to be clarified. How were these features selected?

**Response**: We believe that both the biological and behavioral basis of the 2-sign scoring method are well-described in the text of the paper and supported by relevant references:

- Abstract: “From seven VASA studies conducted in Africa and Asia we developed a 2-sign method based on activity level and feeding behavior…”:
- Page 4 (second paragraph of Introduction) – Behavioral evidence: “A scale across severity levels, especially one based on signs and symptoms easily recognized by and of concern to child caregivers [refs 5,6]…”
- Page 4 (second paragraph of Introduction)– Biological evidence: “…shown to have objective validity, such as activity level and feeding behavior [refs 7,8,9]…”
- Page 26 (first paragraph of Discussion) – Biological and behavioral evidence: “We developed a simple 2-sign score capable of identifying mild, moderate, or severe neonatal illness based on feeding behavior and activity level, signs that have previously been shown to be both objective indicators of neonatal illness severity [refs 7,8,9] and easily recognized motivators of careseeking for sick young children [refs 5,6]. Caregivers have also been found to recognize inability to play, perhaps correlating with our ‘less active’ or ‘not moving’ signs, as indicators of severe fever in children under 5 years in Malawi [ref 27]. Similarly, studies in India and Ghana have found lethargy to be one of the few signs of neonatal illness both easily recognized and understood by mothers to indicate the need for formal health care [refs 28,29].”

**Reviewer comment 4**: as all children included here died, I’m not sure how helpful “mild and moderate” illness descriptions would be as all children actually had severe illness if they died.

**Response**: As described throughout the paper, the analysis assessed the children’s level of illness severity *at the onset of the illness*, hopefully before the child was severely ill and when it would be possible to take effective action to prevent progression to a severe illness and death. This is the main point of the paper, as stated in the Conclusions sub-section of the Discussion: “Guidance on the illness signs for which care should be sought before a young child’s illness progresses to a severe state should be included in child survival messaging and is likely to contribute to decreases in child mortality. The 2-sign method can serve as a practical tool for this purpose for illnesses of neonates, while both the 2-sign and multiple sign methods can be included in mortality and morbidity studies to monitor caregivers’ response to their children’s illness condition and the impact of careseeking messages on child mortality.”

Unfortunately, many young children, especially the youngest neonates, are severely ill at the onset of a fatal illness, and the Conclusions sub-section also addresses this issue: “Qualitative studies are needed to better understand the reasons for reduced health careseeking for younger neonates and infants with a potentially fatal illness. However, it stands to reason that appropriate delivery and newborn care must be provided at community level to prevent and treat intrapartum-related events such as perinatal asphyxia and other early onset severe neonatal illnesses that progress rapidly and for which caregivers are unable to undertake effective careseeking.”

**Reviewer comment 5:** I appreciate the thoughtful representation of authors from each study site. That said, it struck me as odd that in this multi-national study that included primary data collected in Tanzania did not have a Tanzanian co-author. Might the authors explain why?

**Response**: The potential co-author from Tanzania could not review the manuscript in time for its original submission, so was not able to meet all the authorship requirements. He has now reviewed the paper and provided his comments so, happily, is included as a co-author of the revised manuscript.

--------------------------------------------------------------------------------------

More specific comments by section are below. I hope the authors find these helpful as they strengthen the reporting of their important work.

Data Availability:

**Reviewer comment 6**: It does not look like “All data underlying the findings described in the paper are in the main body of the paper and in the Supporting Information file.” I defer to the journal, but I believe they are interested in having access to raw data should replication of analyses be done in the future.

**Response**: This was discussed with the journal editor, who advised as commented by the reviewer. With this submission, all data underlying the findings described in the revised manuscript will be uploaded as supplemental files to editorialmanager.com. I understand that PLOS Global Public Health will upload data files that can be made publicly accessible to figshare if the manuscript is accepted for publication. Five of the seven countries whose data were utilized for the paper’s analyses allow their data to be made open access, while two of the countries require that data requestors contact a country representative to request their data. Details, including how these data may be requested, are provided in the Data Accessibility Statement.

Abstract:

**Reviewer comment 7**: The authors state, “VA and VASA…ask about severe symptoms such as severe cough or inability to suckle and possibly mild or moderate symptoms…without examining progression of the child’s condition” and then state they used the very data source (i.e., VASA) for their analyses that examine “the relationship of caregivers’ perception of their child’s condition at illness onset to the child’s age and seeking formal health care”, which implies some temporality. The authors then conclude “Child mortality studies should assess severity level and caregivers’ response at various illness stages”, but how can this be a conclusion if the VASA does not assess temporality as the authors state earlier in the Abstract. Can the authors please clarify? The Introduction states that the VASA includes temporality, but the Abstract reads otherwise.

**Response**: We thank the reviewer for pointing out this error in the abstract. We have revised the abstract to read as follows: “Child mortality studies, including verbal autopsy (VA) studies, which focus on the biological cause of death, typically ask about severe symptoms such a cough or inability to suckle…” “Verbal and social autopsy (VASA) is a newer method that builds on VA by also examining household, community and health system factors related to the death. From seven VASA studies conducted in Africa and Asia we developed a 2-sign method… We then examined the relationship of caregivers’ perception of their child’s condition at onset of a fatal illness to the child’s age and seeking formal health care at any time during the illness.”

The VASA’s examination of the temporality of illness severity is described in the paragraph preceding Panel 1: “The first method consists of a simple 2-sign severity score based on reported activity level and feeding behavior (Panel 1). Caregivers were asked about these signs when they first noted the child was ill (defined as at onset), when it was decided to seek formal health care for the illness, and when the child left the first health provider alive.” The paper states in multiple locations that the analyses were conducted of the association of careseeking with illness severity *at onset of the illness (neonates) or on illness day 1 (1-11-month-olds), i.e., only at the first stage of illness temporality*, and that the reason for this is that effective careseeking must occur prior to the illness condition becoming severe, e.g., in the Abstract: “Behavior change messages encouraging health careseeking for signs of moderate illness of neonates and infants should be developed.” And the more extensive description of this reason in the Conclusions sub-section of the Discussion (also provided above in response to reviewer comment 4).

**Reviewer comment 8**: It would be immensely helpful to describe the outcome of “mild, moderate and severe illness” better in the Abstract. This description appears to be all encompassing making it hard to understand the utility of a 2-sign method; and

**Reviewer comment 9**: I would suggest including more objective data in the Abstract to delineate the discriminatory value of these tools.

**Response**: We have combined our responses to reviewer comments 8 and 9 by better describing the outcomes of mild, moderate and severe illness in the Abstract and adding report of the objective data and statistical findings for these outcomes. The original sentences in question read: “Formal careseeking was almost uniformly decreased for severely ill neonates, but this was due to their being younger than non-critically ill neonates. Moderately ill infants were somewhat older than the mildly and severely ill and, just as for neonates, formal careseeking was driven primarily by older age.”

These sentences have been modified to read: “By univariate analysis, formal careseeking was almost uniformly decreased for severely ill neonates compared to those who were mildly or moderately ill (8.4%-41.8% vs, respectively, 15.0%-66.7% and 30.5%-68.5%, p=0.122 to <0.001), but multivariate analysis revealed that older age in all six African countries (OR 1.11 [95% CI 1.02, 1.21], p=0.017 to OR 1.10 [95% CI 1.04, 1.16], p<0.001) and moderate illness in three (OR 4.83 [95% CI 1.06, 21.96], p=0.042 to OR 4.35 [95% CI 1.59, 11.93], p=0.005) were positively associated with careseeking, while severe illness no longer played a significant role. Moderately ill infants in three of five countries were more likely to seek care than the mildly and severely ill, though not significantly so (64.9%-83.8% vs, respectively, 45.4%-76.5% and 55.8%-71.4%, p=0.27 to 0.099), and in all five countries were somewhat older (μ months=5.9-6.7 vs, respectively, 5.2-6.5 and 5.0-6.4, p=0.75 to 0.11) and, just as for neonates, formal careseeking was driven primarily by older age (OR 1.11 [95% CI 0.97, 1.27], p=0.128 to OR 1.10 [1.03, 1.16], p=0.003; significant in two countries, p=0.054 in a third country).”

Note that this greatly increased the length of the abstract, which may not be acceptable to the journal’s editor.

**Reviewer comment 10**: Line 38: I would suggest avoidance of any causative language such as “this was due to” in this type of study.

**Response**: We have deleted “this was due to” from both sentences. Line 38 (now 43) now reads: “…multivariate analysis revealed that older age… and moderate illness…were positively associated with careseeking…”. This was also relevant for line 382 (now 387), which has been reworded as: “was revealed by the multivariable analysis to in fact be *a positive association between careseeking and moderate illness* in three countries and no association between careseeking and the severity level in two countries.”

Methods:

**Reviewer comment 11a**: Please include a study design. I appreciate the description of how the VASA was conducted differently in the different countries. That said, if some of these data were collected in a census, might the authors comment on when the interviews were conducted in relation to the death? This seems critically important as recall bias would certainly be an issue, especially when talking about nuanced temporal details about an exceptionally traumatic event.

**Response**: This comment was largely addressed by our addition of the years of the study deaths and VASA interviews to the first paragraph of the Materials and Methods section in response to reviewer comment 2. To further clarify the study design, we have described the study as a secondary analysis of VASA data and added the words “cross-sectional” to the description of the retrospective studies and “prospective” to the description of the Mozambique sample registration system. The full sentence now reads as: “The Mozambique data were collected as part of a prospective national sample registration system with VASA, while the data for all other countries are from cross-sectional retrospective studies of deaths identified by a household survey (Niger, Tanzania, Nigeria, Malawi, Pakistan) or census (Cameroon).” The years of the deaths identified by the Cameroon census and of the VASA interviews of those deaths also make clear that those interviews were conducted retrospectively. The potential for recall bias related to the study design has also been addressed by our response to reviewer comment 2.

**Reviewer comment 11b**: As above, I am not sure I entirely understand how illness severity could be classified given that the data source is one that exclusively included cases of mortality, which by default was preceded by or associated with severe illness.

**Response**: A brief response to this comment is that the paper’s analysis is of caregivers’ response to illness severity *at the onset of a fatal illness*, which in many cases was of mild or moderate severity. Detailed responses are provided above to reviewer comments 1, 4 and 7.

-Minor comment, but

**Reviewer comment 12**: lines 152 and 153 say [reference] instead of including the reference.

**Response**: “Reference” in these instances (now lines 174 and 175) indicate that the value for all other causes of death is the reference for neonatal IPRE or prematurity and for 1-11-month-old severe febrile infection in the multivariable analyses. For clarity-sake, “reference” has been modified to read “reference value.”

**Reviewer comment 13**: Again, it’s not clear to me how the 2 signs were selected as the most optimal to identify illness severity. Please include some description of this approach.

**Response**: This concern of the reviewer was addressed above in response to comment 3.

Results:

**Reviewer comment 14**: Please include a paragraph explaining the demographics of the included cases. I don’t see basic statistics like the number of cases, where they came from, sex, age., etc.

**Response**: Five of the seven VASA studies were conducted nationally. The names of the districts in the 2- and 3-district studies (Cameroon and Malawi) have been added to the first paragraph of the Materials and Methods section, i.e., where the cases came from: “The data for this analysis come from five national VASA studies conducted in Niger [2], Tanzania [3], Nigeria [14], Mozambique [15] and Pakistan, and three- and two-district VASA studies, respectively, in Cameroon (Doume, Nguelemendouka and Abong-Mbang districts) [16] and Malawi (Balaka and Salima districts) [17].” In addition, new sub-section ‘Study population’ including new tables 1a and 1b of demographic characteristics and accompanying text have been added to the start of the paper’s Results section.

**Reviewer comment 15**: What kind of care was sought as the outcome?

**Response**: Throughout the paper, the health care seeking outcome is seeking formal health care. We have added the definition of formal health care, which coincides with the VASA’s question wording and possible responses, as “care provided by a health professional (doctor, nurse, trained health worker) in a hospital, health center, health post or private office, or by a trained community health worker, nurse or midwife.” This definition now appears as part of the first sentence of the Illness recognition and careseeking sub-section of the paper’s Materials and Methods section.

**Reviewer comment 16**: I appreciate the inclusion of Tables 1 and 2 that shows site-specific categorization, but isn’t it important to also show overall performance of these approaches by pooling data all sites?

**Response**: We believe that the reviewer means tables 3 and 4 (now 4 and 5). (Tables 1 and 2 [now 2 and 3] provide the comparison of the 2-sign and multiple sign methods of assessing illness severity, while tables 3 and 4 [now 4 and 5] examine univariate and multivariate associations with careseeking.) Regarding the reviewer’s comment, the illness severity measures developed by the study are meant to be used at the individual site level and we expected there to be substantial heterogeneity between sites, in which case the overall performance would be less informative than the site specific. Pooling the data would likely strengthen the statistical significance of the findings, but to what end? We expect that those considering use of the illness severity score would consider site-specific performance to be more relevant than overall performance.

**Reviewer comment 17**: Line 238: Consider rewording this. As is, it seems that caregivers were less likely to seek care for more severe illness. Is that what is meant?

Response: The line referenced by the reviewer (was 228, is now 289) is discussing table 3 (now 4), which indeed shows a (univariate) “negative association between illness severity and formal careseeking for neonates in two of the six African countries, and an apparent negative or near-negative association in three other countries that is punctuated by greater careseeking for moderate than either mild or severe illness (figure 1a).” Therefore, the answer to “Is that what is meant”? is most often ‘Yes.’ The study analysis found that caregivers in most countries were less likely to seek formal health care for neonates who were severely ill, compared to those who were mildly or moderately ill, at the onset of a fatal illness. However, severely ill neonates were younger than the mildly and moderately ill and as shown by the multivariate analysis, once age was considered illness severity was no longer a significant factor in determining health care seeking. This was a major finding of the study and was discussed extensively.

**Reviewer comment 18**: Table 4 is helpful, but I suspect the authors are not powered for this type of analysis for some countries (e.g., Tanzania and Cameroon). Might the authors defend their sample size and power the this regression analysis in the Methods? (Again, I’m not sure why the authors chose not to conduct pooled analyses across the sites.

**Response**: We agree that the sample size may be small for some countries when examining factors relating to having sought formal care.  We have acknowledged this as a limitation in the discussion, stating, ”From a statistical standpoint, power may be limited for some countries when examining the associations of illness severity and other factors with seeking formal health care.” However, as discussed above (comment 16), we believe that a pooled analysis would be contrary to the aims of this study, which sought to examine the utility of the developed methods for determining the relationship of illness severity to careseeking at the site-specific level. Also, please note that despite the limited sample sizes in Tanzania and Cameroon, the overall findings for those countries were similar to those of other countries with larger samples, i.e., severe illness by univariate analysis was significantly or nearly significantly associated with decreased careseeking for neonates but in the multivariate analyses age, and not illness severity, was significantly associated with careseeking for both neonates and 1-11-month-olds.

Discussion:

**Reviewer comment 19**: In general, this is well-written, but I think it would benefit from more of an explanation of how the authors think these scoring systems could be practically used, especially since all cases died here so they all eventually had severe illness.

**Response**: As the reviewer stated, all the children included in our study eventually had severe illness and died. That is why we focused the study on the children’s condition at the onset of their illness, when many might have been mildly or moderately ill and effective action might have been taken to prevent progression to a severe condition and death. As stated by the paper’s Conclusion, “Guidance on the illness signs for which care should be sought before a young child’s illness progresses to a severe state should be included in child survival messaging and is likely to contribute to decreases in child mortality. The 2-sign method can serve as a practical tool for this purpose for illnesses of neonates, while both the 2-sign and multiple sign methods can be included in mortality and morbidity studies to monitor caregivers’ response to their children’s illness condition and the impact of careseeking messages on child mortality.” We believe that this statement is sufficient explanation of how the scoring systems can be practically used.
