# Supplementary material for: Development of an effective illness severity measure and assessment of the impact of perceived illness severity on formal careseeking for fatal illnesses of neonates and infants in six sub-Saharan Africa countries and Pakistan
Source: PLOS Glob Public Health. 2026 May 22;6(5):e0006455. doi: 10.1371/journal.pgph.0006455 (PMC13197068; doi:10.1371/journal.pgph.0006455)
Supplement: Attachment — Submitted filename: Response to Reviewers_03272026.docx [file pgph.0006455.s014.docx]

**Journal requirements:**

Please include the following items when submitting your revised manuscript:

1. A letter that responds to each point raised by the editor and reviewer(s). You should upload this letter as a separate file labeled 'Response to Reviewers'.

**Response**: A file labeled ‘Response to Reviewers_03272026’ has been uploaded to editorialmanager.com as part of the second revised manuscript submission.

2. A marked-up copy of your manuscript that highlights changes made to the original version. You should upload this as a separate file labeled 'Revised Manuscript with Track Changes'.

**Response**: A marked-up copy of our manuscript labeled 'Revised Manuscript_ tracked_03272026' has been uploaded to editorialmanager.com. As requested, this file includes highlighted changes made to the original version of the manuscript. It also includes highlighted changes made to the first revised version of the manuscript based on the reviewer’s current comments on the first revised version.

3. An unmarked version of your revised paper without tracked changes. You should upload this as a separate file labeled 'Manuscript'.

**Response**: An unmarked version of our revised paper labeled ‘Revised manuscript_clean_03272026’ without tracked changes has been uploaded as a separate file to editorialmanager.com.

Reviewers' comments:

Reviewer's Responses to Questions

**Comments to the Author**

1. If the authors have adequately addressed your comments raised in a previous round of review and you feel that this manuscript is now acceptable for publication, you may indicate that here to bypass the “Comments to the Author” section, enter your conflict of interest statement in the “Confidential to Editor” section, and submit your "Accept" recommendation.

Reviewer #1: (No Response)

2. Does this manuscript meet PLOS Global Public Health’s [publication criteria](https://nam02.safelinks.protection.outlook.com/?url=https%3A%2F%2Ftrack.editorialmanager.com%2FCL0%2Fhttps%3A%252F%252Fjournals.plos.org%252Fglobalpublichealth%252Fs%252Fcriteria-for-publication%2F1%2F010f019d1d5523ca-2edd8e6a-b948-4efa-845a-825836cac519-000000%2FnSq6g3Qh8xIAMxafNOAbal4df9NzXDRLikIpoOEZutM%3D255&data=05%7C02%7Chkalter1%40jhu.edu%7C64e98d44ccd94f4e3f9108de893fd5db%7C9fa4f438b1e6473b803f86f8aedf0dec%7C0%7C0%7C639099104367821246%7CUnknown%7CTWFpbGZsb3d8eyJFbXB0eU1hcGkiOnRydWUsIlYiOiIwLjAuMDAwMCIsIlAiOiJXaW4zMiIsIkFOIjoiTWFpbCIsIldUIjoyfQ%3D%3D%7C0%7C%7C%7C&sdata=onRjTL6kR5VLWca27563ve0R4Z0LZheB7R05%2FxA96Zw%3D&reserved=0)? Is the manuscript technically sound, and do the data support the conclusions? The manuscript must describe methodologically and ethically rigorous research with conclusions that are appropriately drawn based on the data presented.

Reviewer #1: Yes

3. Has the statistical analysis been performed appropriately and rigorously?

Reviewer #1: Yes

4. Have the authors made all data underlying the findings in their manuscript fully available (please refer to the Data Availability Statement at the start of the manuscript PDF file)?

The [PLOS Data policy](https://nam02.safelinks.protection.outlook.com/?url=https%3A%2F%2Ftrack.editorialmanager.com%2FCL0%2Fhttps%3A%252F%252Fjournals.plos.org%252Fglobalpublichealth%252Fs%252Fdata-availability%2F1%2F010f019d1d5523ca-2edd8e6a-b948-4efa-845a-825836cac519-000000%2FF2BQe1hMjNdTPRj41ZJnZwzCeuMb-ZmyUVvEyq3gGl4%3D255&data=05%7C02%7Chkalter1%40jhu.edu%7C64e98d44ccd94f4e3f9108de893fd5db%7C9fa4f438b1e6473b803f86f8aedf0dec%7C0%7C0%7C639099104367845405%7CUnknown%7CTWFpbGZsb3d8eyJFbXB0eU1hcGkiOnRydWUsIlYiOiIwLjAuMDAwMCIsIlAiOiJXaW4zMiIsIkFOIjoiTWFpbCIsIldUIjoyfQ%3D%3D%7C0%7C%7C%7C&sdata=WD1NSLEMWDPz3LLa%2BO7Ny2B6e%2FP95xndIbZZMLT4h5E%3D&reserved=0) requires authors to make all data underlying the findings described in their manuscript fully available without restriction, with rare exception. The data should be provided as part of the manuscript or its supporting information, or deposited to a public repository. For example, in addition to summary statistics, the data points behind means, medians and variance measures should be available. If there are restrictions on publicly sharing data—e.g. participant privacy or use of data from a third party—those must be specified.

Reviewer #1: Yes

5. Is the manuscript presented in an intelligible fashion and written in standard English?

PLOS Global Public Health does not copyedit accepted manuscripts, so the language in submitted articles must be clear, correct, and unambiguous. Any typographical or grammatical errors should be corrected at revision, so please note any specific errors here.

Reviewer #1: Yes

6. Review Comments to the Author

Please use the space provided to explain your answers to the questions above. You may also include additional comments for the author, including concerns about dual publication, research ethics, or publication ethics. (Please upload your review as an attachment if it exceeds 20,000 characters)

Reviewer #1: I appreciate the authors’ responsiveness to the peer review process. I think the manuscript reads much better at this point but I do have one concern that remains.
I’m not certain that the authors have adequately acknowledged the potential for recall bias. I had pointed out previously a concern about a need for “a clear description of when these interviews were conducted relative to the child’s death and the events they reported about.” The authors largely replied by including the years that the surveys were conducted in each country and simply added the following to the Limitations, “Being interview-based, for the most part conducted from one to five years after the death, the findings are also subject to the possible influence of faulty recall and biased reporting of events.” Surely the authors agree that an interview conducted as much as five years after a death may have some reliability concerns when asking caregivers about the “activity level and feeding behavior” “at the onset of the fatal illness”. I have two suggestions to be more explicit about this. Could the authors include some data on the timing of the interview in relation to the time of the death? Might they acknowledge this timeframe between death and the interview a bit more explicitly in the Methods?

**Response**: Please note that the addition of the years when the surveys were conducted in each country that the reviewer commented on included both the years when the platform surveys that identified the child deaths were conducted as well as the years when the follow-up VASA interviews were conducted. This allows a reasonable approximation of the recall period from death to VASA interview. To further clarify this, we have added the mean and median years of the VASA interview recall period and additional specific data to the text, as follows:

“The years of the deaths / conduct of VASA interviews (mean, median years interview recall period) in each country were: Cameroon 2006-10 / 2012 (3.2, 3), Nigeria 2008-13 / 2014 (3.7, 4), Malawi 2008-12 / 2013 (3.4, 3), Niger 2007-10 / 2012 (3.3, 3), Tanzania 2010-15 / 2017 (4.2, 4), Mozambique 2018-20 / 2018-20 (0.2, 0), and Pakistan 2015-19 / 2018-19 (1.5, 1). In five countries 13% to 42% of interviews were conducted more than four years after the death, while in Pakistan and Mozambique, respectively, 97% were conducted in less than four years and 100% in less than two years.”

In accord with this additional data, the related sentence in the paper’s Limitations sub-section has been revised as follows:

“Being interview-based, with some interviews conducted beyond four years in five of the study countries, the findings are also subject to the possible influence of faulty recall and biased reporting of events.”

Further details on the interview recall period (summarized in the revised paper and provided here for the reviewer’s information but too lengthy to include in the paper) follow:

Cameroon: 2=0.34, 3=0.26, 4=0.27, 5=0.07, 6=0.06

- <4 years=60%, <5 years=87%, >4 years=13%

Nigeria: 1=0.06, 2=0.21, 3=0.15, 4=0.20, 5=0.21, 6=0.17

- <4 years=42%, <5 years=62%, >4 years=38%

Malawi: 1=0.02, 2=0.25, 3=0.27, 4=0.24, 5=0.22

- <4 years=54%, <5 years=78%, >4 years=22%

Niger: 2=0.29, 3=0.33, 4=0.23, 5=0.16

- <4 years=62%, <5 years=85%, >4 years=16%

Tanzania: 1=0.01, 2=0.16, 3=0.21, 4=0.19, 5=0.18, 6=0.19, 7=0.05, 8=0.003

- <4 years=38%, <5 years=57%, >4 years=42%

Mozambique: 0=0.77, 1=0.23

- <2 years=100%

Pakistan: 0=0.17, 1=0.36, 2=0.26, 3=0.18, 4=0.02, 5=0.003, 6=0.0005

- <4 years=97%, <5 years=99%

Figure Resubmissions:

While revising your submission, we strongly recommend that you use PLOS’s NAAS tool (<https://ngplosjournals.pagemajik.ai/artanalysis>) to test your figure files. NAAS can convert your figure files to the TIFF file type and meet basic requirements (such as print size, resolution), or provide you with a report on issues that do not meet our requirements and that NAAS cannot fix.

**Response**: The figure files submitted with the first revision were checked with the NAAS tool and found to meet all requirements.
